# Advancing morphological profiling with self-supervised learning through comparative insights into representation learning of cellular perturbations

**DOI:** 10.1038/s41592-026-03270-z

**Published:** 2026-10-29

**Authors:** Walid M. Abdelmoula, Tomé Albuquerque, Steffen Jaensch, Pushpak Pati, Michiel Van Dyck, Samantha J. Allen, Litao Zhang, Tommaso Mansi, Rui Liao

**Affiliations:** 1 Data Science and AI, Johnson & Johnson, Cambridge, MA USA; 2 Data Science and AI, Johnson & Johnson, Zug, Switzerland; 3 Discovery Technologies and Molecular Pharmacology, Johnson & Johnson, Beerse, Belgium; 4 Cognitive Automation and Technology, Johnson & Johnson, Beerse, Belgium; 5 Discovery Technologies and Molecular Pharmacology, Johnson & Johnson, Spring House, PA USA; 6 Data Science and AI, Johnson & Johnson, Titusville, NJ USA

**Keywords:** Machine learning, Image processing

## Abstract

Self-supervised learning (SSL) enables scalable, label-free foundation models (FMs) that learn task-agnostic representations directly from images, bypassing cell segmentation and feature engineering inherent to classical morphological profiling. We present a large-scale evaluation of SSL FMs for image-based phenotypic profiling, covering contrastive (DINO), masked-based generative (MAE, iTPN) and hybrid (DINOv2) learning mechanisms, pretrained on all unannotated chemical perturbations from the Joint Undertaking for Morphological Profiling–Cell Painting (JUMP–CP) dataset (119,876 compounds, ~1.77 million images, 7 laboratories). Here we show that top-performing SSL models outperformed CellProfiler in compound-replicate retrieval on the held-out JUMP–Target2 set (302 annotated compounds, *P* ≤ 0.001). DINO and DINOv2 generalized across diverse perturbations and cell types, outperforming CellProfiler with 31–37% gains in site-averaged mean average precision for mechanism-of-action retrieval on EU-OPENSCREEN Bioactives (2,464 compounds), and consistently surpassing masked-based models across all benchmarks. This study provides a systematic assessment of SSL approaches for advancing morphological profiling and offers pretrained FMs to accelerate drug discovery.

## Abstract (web summary)

This Article presents a large-scale analysis of self-supervised learning methods for high-content morphological profiling.

## Main

Recent advances in high-throughput imaging and morphological profiling have shown promise for impacting drug discovery through insights derived from image-based signatures of chemically or genetically perturbed cells^1,2^. Cell painting is a high-content imaging (HCI) assay that provides a powerful approach for profiling cellular morphology^3^. This promising technique enables the acquisition of rich biological information by staining cells with a set of fluorescent dyes targeting different subcellular structures, such as the nucleus, cytoplasm, mitochondria and more^3,4^. Typically, cells are arrayed in multi-well plates, with each well receiving a certain treatment, followed by automated microscopy. This approach has shown utility from hit identification^5^ to mechanism of action (MoA) determination^6^, and prediction of molecular activity and toxicity^7–9^.

The Joint Undertaking for Morphological Profiling–Cell Painting (JUMP–CP) consortium represents a notable collaborative effort to standardize and expand the use of cell painting assays^6^. This large-scale open-source initiative has generated over 100 TB of data, encompassing millions of high-content images from more than 120,000 chemical and genetic perturbations acquired across 12 pharmaceutical companies and academic institutions (that is, sources). The sheer volume and diversity of these data offer unprecedented opportunities for accelerating drug discovery by fostering innovative computational methods using advanced machine learning techniques.

Machine learning and computer vision techniques have been central in cell painting data analysis^5,7–9^. Computational methods can be grouped into classical and deep learning-based approaches. CellProfiler^10^, among the most widely used tools for classical morphological profiling, relies on cell segmentation followed by extraction of handcrafted morphological features to enable downstream analysis such as bioactivity predictions^11^. These extracted features typically undergo a further processing step to reduce feature redundancy by filtering out highly correlated and low-variance features^12^. While successful in many applications, this dependence on segmentation and predefined features can introduce inefficiencies and biases that constrain the scalability and full potential of cell painting data^5,13^.

Deep learning methods have shown promise as alternatives to classical approaches for handling the complexity of HCI data, including massive multi-channel datasets with sparse compound annotations^14–18^. Several studies have shown that supervised and weakly supervised models can outperform CellProfiler-based approaches in predicting biological activity^14,19–21^, offering end-to-end learning directly from raw images without cell segmentation. However, these models depend heavily on the availability of annotated compound activities, which are scarce and sparse in the cell painting field, limiting their ability to generalize to unseen compounds^22^.

Foundation models (FMs) based on self-supervised learning (SSL) have emerged as a transformative alternative, addressing this annotation bottleneck by learning robust feature representations from large-scale unlabeled data^23–28^. SSL provides task-agnostic representations that can be effectively used for diverse downstream analyses with limited or no labels^29–31^, making these methods particularly well suited for HCI and drug discovery applications^27,32,33^. Vision transformers (ViTs) represent a robust backbone architecture for vision FMs, modeling images as sequences of patches and leveraging self-attention to capture both global image-level and local patch-level representations^34,35^.

The learning mechanisms underlying SSL can be broadly classified into contrastive, generative and hybrid methods^36^. Generally, contrastive-based methods learn by comparing augmented views of the data, whereas generative-based methods learn by reconstructing masked regions or the entire input data. Early contrastive learning approaches relied on discriminating between positive and negative image views to learn representations (for example, simple framework for contrastive learning of visual representations (SimCLR)^37^ or momentum contrast (MoCo)^38^). More recently, methods such as bootstrap your own latent (BYOL)^39^ and DINO^34^ learn representations from positive views without negative sampling, relying instead on self-distillation between student and teacher networks. Unlike contrastive learning, masked-based models (for example, image BERT pre-training with online tokenizer (iBOT)^40^ and masked autoencoders (MAE)^41^) offer a competitive alternative by learning from masked inputs without relying on data augmentation or negative sampling.

Recent progress in adopting SSL for HCI analysis has shown strong potential compared with classical and supervised or weakly supervised learning approaches^27,32,33^. Contrastive-based methods (SimCLR^32,42,43^, BYOL^44^ and DINO^32,33,43–46^) and masked-based generative models (MAE^27,47^) have been successfully applied to cell painting data. While transfer learning of FMs pretrained on public datasets (for example, ImageNet) showed competitive performance in some applications compared with CellProfiler-like approaches^14,17^, it underperformed when compared with models pretrained specifically on the HCI domain^32,33^. As contrastive and generative approaches use fundamentally different learning mechanisms, they may yield representations of distinct power for various downstream tasks^48^. However, a systematic large-scale benchmarking comparing major learning mechanisms underlying SSL methods—contrastive, generative and hybrid—remains lacking for HCI data. In a previous study^32^ initial steps were taken in this direction, comparing SimCLR, DINO and MAE on a subset of JUMP–CP data (models pretrained on approximately 10,000 compounds).

In this Article, we extend this line of research with a comprehensive, large-scale evaluation covering all three primary categories of SSL learning mechanisms: contrastive-based (Distillation with No Labels (DINO)^34^), masked-based generative (MAE^41^ and integrally pretrained transformer pyramid network (iTPN)^49^) and hybrid (DINOv2^29^) models. Notably, DINOv2 integrates the complementary learning mechanisms of DINO and iBOT, seamlessly combining contrastive-based and masked-based approaches. Additionally, we adopted iTPN, a recent advance in masked-based generative modeling equipped with a hierarchical vision transformer (HiViT) that enables concurrent optimization of multi-scale feature learning. We pretrained these models on all unannotated chemical perturbations from JUMP–CP, totaling 119,876 compounds (~1.77 million images) from 7 pharmaceutical laboratories across 87 experimental batches—a substantial scale-up compared with previous work^32^. We conducted extensive evaluation on the held-out JUMP–Target2–compound set (302 compounds, 160 gene targets, 8 sources), incorporating all available sources and experimental batches to ensure a comprehensive assessment. Moreover, we assessed generalizability on three independent public datasets (BBBC021 (refs. ^50,51^), RxRx1 (ref. ^52^) and EU-OPENSCREEN Bioactives^53^) spanning four cell types (for example, MCF-7, HUVEC, RPE and HepG2), different fluorescent markers and diverse chemical and genetic perturbations. We further evaluated the impact of training strategies and batch-correction methods on representation quality. Our results show the robustness and biological relevance of the SSL-derived features, which outperformed its CellProfiler counterpart without requiring any cell segmentation or feature engineering. Furthermore, we show that the DINO and DINOv2 models learn more robust representations compared with those learned by masked-based models. This study provides a large-scale systematic benchmark across major SSL learning mechanisms for morphological profiling, offering practical guidance on selecting SSL approaches and pretrained FMs to advance high-content image-based phenotypic profiling in drug discovery.

## Results

### Proposed pipeline for HCI-based representation learning

**Fig. 1:**
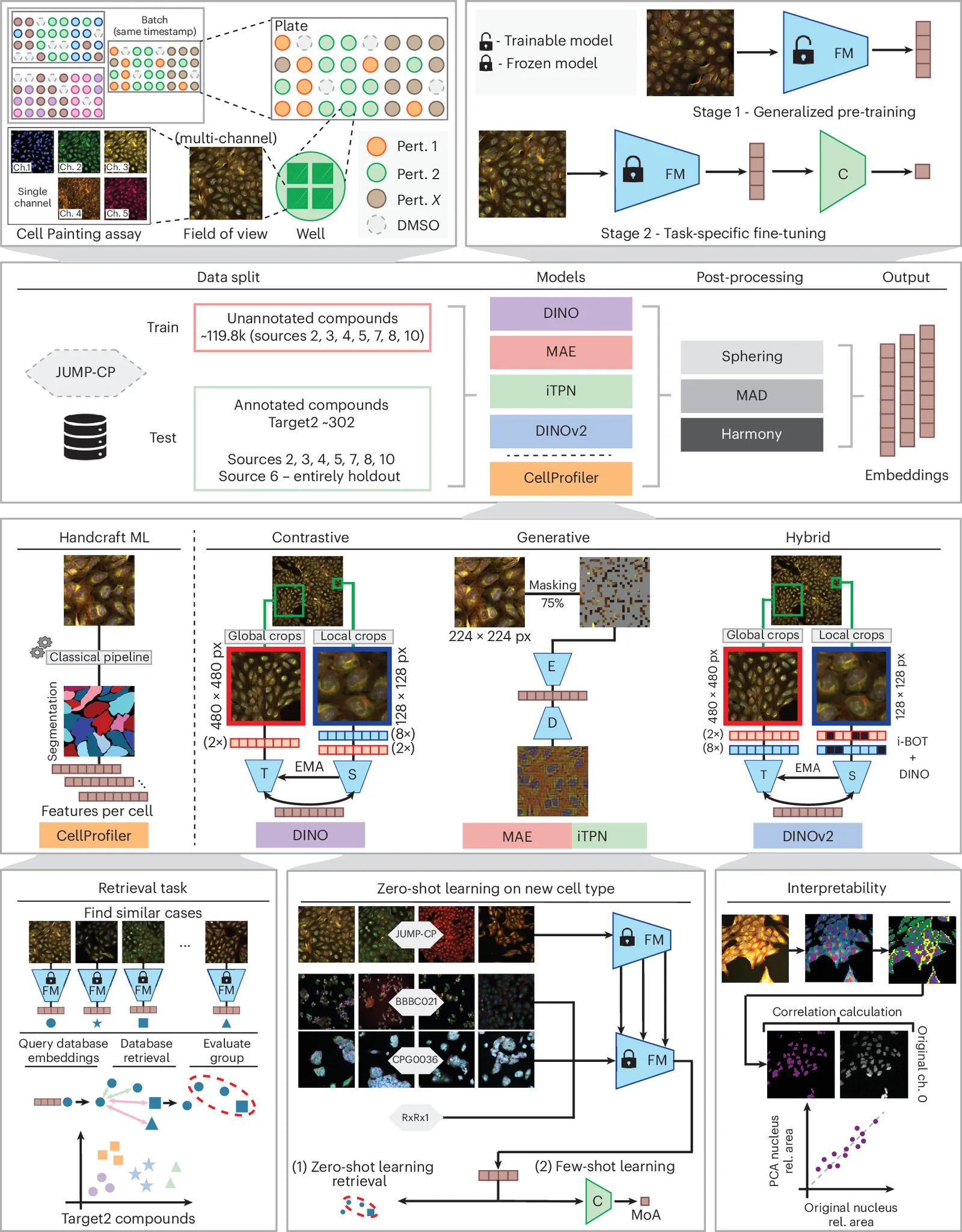
Framework for comparative assessment of self-supervised representation learning in cell painting. Overview of the proposed framework for comparative assessment of representation learning in cell painting using four SSL FMs, encompassing three major learning mechanisms: contrastive-based models (DINO), masked-based generative models (MAE and iTPN) and a hybrid model (DINOv2). The FMs were trained on all unannotated JUMP–CP data and evaluated on a held-out annotated subset. In the downstream analysis, the pretrained FMs were locked and used as feature extractors. The downstream analyses cover various tasks, including zero-shot learning for compound retrieval tasks and few-shot learning to predict MoAs from a different cell type. An interpretability module provides visual insights into the SSL local representations learned through knowledge distillation. C, classifier; ch., single channel; Pert., perturbation; rel., relative; E, encoder; D, decoder; S, student; T, teacher; EMA, exponential moving average.

We pretrained and benchmarked FMs for representation learning from multi-channel high-content images using the workflow summarized in Fig. 1. The models cover four SSL methods spanning three major learning mechanisms: masked-based generative (MAE, iTPN), contrastive-based (DINO) and hybrid (DINOv2). Unlike CellProfiler, our pipeline operates directly at the image level without requiring cell segmentation or feature engineering.

The FMs were pretrained and benchmarked using a two-stage approach (Fig. 1). In the pretraining phase, all unannotated chemical perturbations from the JUMP–CP dataset were used for modeling optimization to learn general feature representations. In the evaluation phase, pretrained weights were frozen and used solely as feature extractors for two downstream tasks: zero-shot compound retrieval, where embeddings were directly used to assess reproducibility and biological relevance, and few-shot MoA classification, where a *k*-nearest neighbor (*k*-NN) classifier was trained on the extracted embeddings. Postprocessing strategies (sphering, plate-wise median absolute deviation (MAD) normalization and Harmony) were evaluated to mitigate batch effects and improve downstream performance (Methods).

### Postprocessing strategies for batch effect correction and improved SSL feature representation

**Fig. 2:**
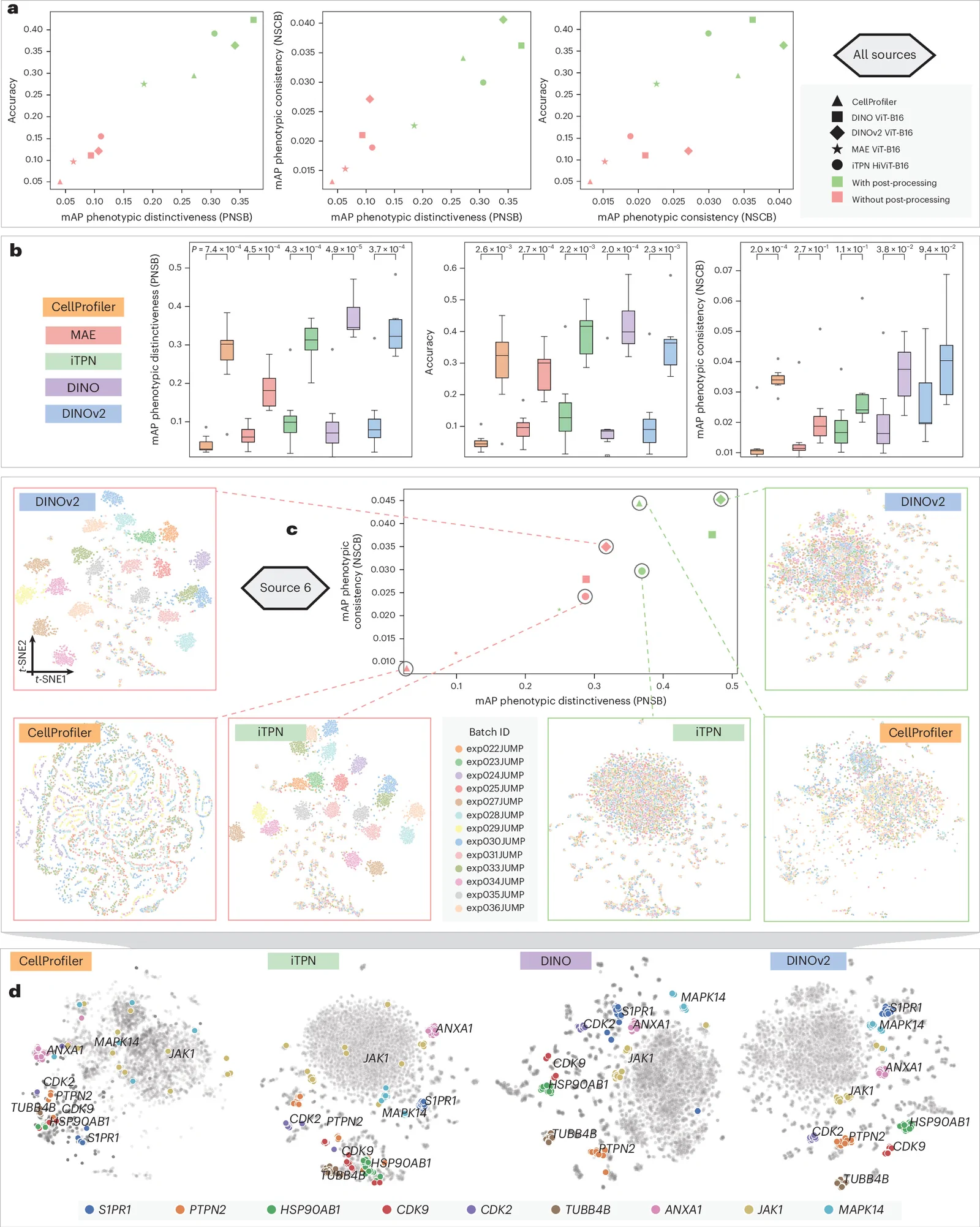
Postprocessing of the learned representation for batch effect mitigation shown on JUMP–Target2–compounds. **a**, Distribution of the overall retrieval performance across all sources and different methods, before and after postprocessing, on downstream tasks of phenotypic distinctiveness (PNSB) for reproducibility and phenotypic consistency (NSCB) for biological relevance. The overall performance was evaluated using mAP and accuracy metrices, where accuracy reflects the portion of correctly retrieved data points out of the total data points. **b**, Postprocessing significantly improved the reproducibility task for all models, whereas the biological relevance task was significantly improved only for CellProfiler and DINO-based approaches. Box plots show the median (center line), interquartile range (IQR; box bounds, 25th–75th percentiles) and whiskers extending to 1.5 × IQR; outliers are shown as individual points. Each box plot has 7 data points per method (each data point represents the source-level mAP). Exact *P*values, from two-sided Welch’s *t*-tests, comparing raw against postprocessed features for each method are shown above the box plots (**b**). **c**, Example from source 6 (entirely held out during the pretraining phase) showing the detailed distribution of the learned representations (average aggregated at the well level) before and after postprocessing, revealing improved representation after postprocessing, where data points form biologically relevant clusters (**d**) rather than clustering based on experimental batches (**c**). **d**, Distribution of nine retrieved targets based on the NSCB task (mAP ≥ 0.25), where each target is represented by two compounds. While six of these targets were retrieved by the different methods at the retrieval threshold (mAP ≥ 0.25), DINOv2 showed greater robustness by retrieving three additional targets (*MAPK14*, *JAK1*and *CDK9*), forming more compact clusters compared with those learned by other methods, specifically CellProfiler.

We conducted a detailed ablation of three postprocessing methods—sphering, MAD normalization and Harmony—examining their effects individually and in combination (Supplementary Fig. 1 and Methods). For SSL-derived features, postprocessing steps were sequentially applied in the order: sphering, MAD, then Harmony, with various combinations tested. The ordered sequential combination of all three methods provided the best results for SSL-derived features. For CellProfiler, we followed standard practice^12,54^, normalizing features first using MAD and then applying feature selection before applying sphering and/or Harmony, which yielded only marginal additional gains, primarily for the reproducibility task (Supplementary Fig. 1). After postprocessing, all SSL methods except MAE consistently outperformed CellProfiler on the JUMP–Target2–compounds retrieval tasks (Fig. 2a). For the reproducibility task of phenotypic distinctiveness (perturbation-not-same-batch (PNSB)), DINO achieved the highest mean average precision (mAP), whereas DINOv2 outperformed all other methods on the biological relevance task of phenotypic consistency (not-same-compound-and-batch (NSCB)). This agrees with other studies, from various domains^26,32,48^, which showed contrastive-based and hybrid-based SSL approaches are strong learners compared with masked-based approaches. Among masked-based models, iTPN achieved better scores than MAE. Postprocessing significantly improved the reproducibility task for all models (Fig. 2b); however, for biological relevance, significant improvements were observed only for CellProfiler and DINO-based approaches. A detailed source-wise analysis of gene target retrieval across all methods is provided in Supplementary Table 1.

As source 6 was entirely held out during pretraining, we used it to demonstrate the quality of the learned representations and their biological relevance (Fig. 2c,d). The *t*-distributed stochastic neighbor embedding (*t*-SNE) maps illustrate that postprocessing effectively mitigated severe batch effects present in the raw features (Fig. 2c). Evaluation of the biological relevance task on source 6 across all methods (mAP ≥ 0.25) revealed retrieval of nine gene targets (Supplementary Table 1), where each target is represented by replicates from two different perturbations (Supplementary Fig. 2). While six targets were well retrieved by CellProfiler, iTPN, DINO and DINOv2 with comparable performance, MAE severely underperformed, retrieving only one target. DINOv2 showed robustness by additionally retrieving three targets (*MAPK14*, *JAK1* and *CDK9*), forming more compact clusters compared with other methods (Fig. 2d and Supplementary Table 2).

Source 6 also provides an opportunity to evaluate the impact of postprocessing for batch effect correction on a larger scale beyond Target2–compounds. Therefore, we assessed the phenotypic distinctiveness (PNSB) task across all perturbations in source 6, comprising 59,145 compounds across 246 plates and 13 experimental batches. With the exception of the JUMP–Target2–compounds, compounds in source 6 overlapped with those used during pretraining, and therefore, the evaluation primarily reflects image-level rather than compound-level holdout. A total of 982 compounds contributed positive pairs for the phenotypic distinctiveness task (that is, appeared in ≥2 distinct batches), representing 1.66% (982/59,145) of the source 6 compounds. The remaining 58,163 compounds (98.34%) were singletons after batch-level aggregation (that is, present in only one batch) and were included as negatives in retrieval evaluation. Retrieval performance (Table 1) was stratified by replicate availability, with compound counts of 669 (2 replicates), 6 (3–5 replicates) and 307 (≥6 replicates). As shown in Table 1, postprocessing consistently resulted in substantial performance improvements, reflecting enhanced batch effect correction and phenotypic discrimination. Across all models, DINO and DINOv2 achieved the highest performance for both the percentage of retrieved classes (corrected *P* value < 0.05) and mAP (Table 1). Despite the effectiveness of within-source batch correction, aligning data acquired from different sources proved to be more challenging (Supplementary Fig. 3), highlighting the need for advanced methods capable of handling complex inter-laboratory variations.

**Table 1:** Evaluation of batch effect correction on the retrieval task of the phenotypic distinctiveness (PNSB) across different models

Model	Without		With	
	Postprocessing		Postprocessing	
	Retrieved (%)	Mean mAP (%)	Retrieved (%)	Mean mAP (%)
CellProfiler	3.891, 6.67, 23.13	0.07, 4.20, 0.82	42.37, 66.67, 96.74	8.67, 15.31, 30.17
MAE ViT-B8	14.95, 16.67, 40.72	2.13, 2.34, 5.02	26.31, 83.33, 99.67	4.27, 11.44, 16.00
MAE ViT-B16	13.00, 33.33, 51.79	2.16, 6.97, 6.02	31.54, 50.00, 100.00	3.05, 16.42, 16.40
iTPN HiViT-B8	14.80, 50.00, 41.04	3.44, 11.26, 9.42	38.86, 83.33, 100.00	7.80, 16.75, 26.58
iTPN HiViT-B16	19.73, 66.67, 48.21	4.33, 12.68, 14.38	41.85, 83.33, 100.00	8.71, 18.71, 29.78
DINO ViT-B16	9.42, 16.67, 31.92	2.74, 3.73, 10.92	54.56, 83.33, 100.00	13.93, 32.30, 41.63
DINOv2 ViT-B16	11.36, 16.67, 36.16	3.03, 7.71, 12.82	61.14, 83.33, 100.00	13.72, 36.49, 41.95
DINOv2 ViT-B16 cross-batch (no aug)	13.45, 16.67, 42.35	4.24, 3.22, 14.58	53.06, 66.67, 100.00	11.76, 25.15, 37.17
DINOv2 ViT-B16 cross-batch	13.15, 16.67, 44.63	4.61, 2.74, 18.02	60.69, 83.33, 100.00	15.39, 30.37, 44.25
DINOv2 ViT-B16 one source	10.91, 16.67, 31.92	2.77, 9.70, 8.67	54.86, 83.33, 100.00	11.71, 33.93, 35.70
DINOv2 ViT-B16 (short sequence)	9.72, 16.67, 34.85	2.71, 12.09, 11.31	57.10, 83.33, 100.00	14.26, 25.97, 41.38
DINOv2 ViT-B8	9.42, 16.67, 35.18	2.32, 8.27, 10.34	59.64, 83.33, 100.00	13.41, 23.72, 39.81
DINOv2 ViT-B8 (short sequence)	10.46, 16.67, 34.53	3.32, 8.96, 11.65	59.49, 83.33, 100.00	16.33, 29.83, 42.14
DINOv2 ViT-S16	10.16, 16.67, 34.85	2.61, 1.18, 11.73	42.75, 83.33, 100.00	8.56, 13.48, 32.03

Analyses were performed on the held-out source 6 images (59,145 compounds, 246 plates, 13 experimental batches). Performance metrics are stratified by per-compound replicate count: 2 replicates (*n* = 669 compounds), 3–5 replicates (*n* = 6) and ≥6 replicates (*n* = 307). Postprocessing consistently yields substantial performance improvements, reflecting enhanced batch effect correction and phenotypic discrimination. Retrieval values in the table are listed in the following order: 2-replicate, 3–5-replicate and ≥6-replicate groups.

### Comparative performance in compound retrieval: reproducibility and robustness of DINO and DINOv2

**Fig. 3:**
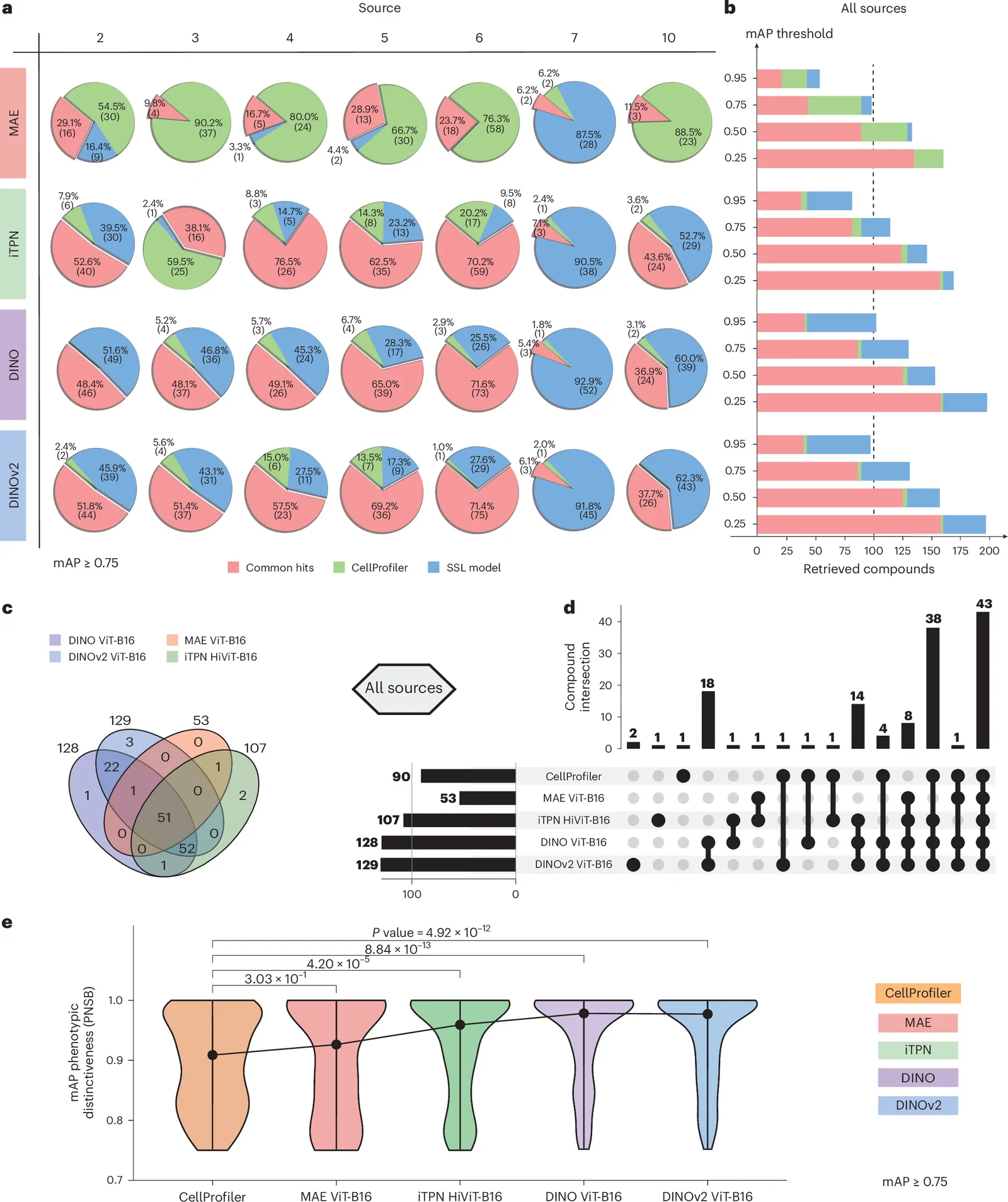
Comparative performance on the downstream reproducibility task of phenotypic distinctiveness (PNSB) between pretrained SSL models and CellProfiler. **a**, Source-wise distribution of retrieved Target2–compounds by different SSL methods compared with CellProfiler according to the PNSB criteria with mAP ≥ 0.75. **b**, Overall comparative performance of all methods for the reproducibility task (PNSB retrieval) at different mAP thresholds, showing the stability and robustness of the top-performing SSL models. **c**, Overlap of compounds retrieved by different SSL models. **d**, Upset diagram showing intersection of PSNB compounds retrieved by different SSL and CellProfiler approaches. **e**, Violin plots showing the distribution of PNSB retrieval performance for each SSL method compared with CellProfiler, from all seven sources. The black dot indicates the median, the thick bar represents the IQR (25th–75th percentiles) and the thin line extends to 1.5 × IQR. Sample sizes per method are as follows: CellProfiler: *n* = 90 compounds (265 data points across batches and sources); MAE ViT-B16: *n* = 53 compounds (101 data points); iTPN HiViT-B16: *n* = 107 compounds (327 data points); DINO ViT-B16: *n* = 128 compounds (491 data points); DINOv2 ViT-B16: *n* = 129 compounds (451 data points). Exact *P*values from two-sided Welch’s *t*-tests are shown above the violin plots (**e**).

We performed a source-wise analysis to assess the comparative performance of the pretrained SSL models on phenotypic distinctiveness (PNSB) and phenotypic consistency (NSCB) tasks (Methods), benchmarking them against CellProfiler. This analysis establishes a practical scenario in which a pretrained model is applied to unseen data generated at different sites using different microscopic systems. A comprehensive source-wise evaluation across all models is provided in Fig. 3 and Supplementary Tables 3 and 4, showing the notable impact of postprocessing on both mAP and the percentage of retrieved treatments. The results reveal overall stability in model performance across sources; however, source 7 showed higher variability, while iTPN, DINO and DINOv2 showed greater robustness. Notably, despite applying the same standard Pycytominer pipeline for CellProfiler feature selection across all sources^12,54^, it yielded suboptimal performance on source 8, where SSL methods showed consistent robustness without additional feature selection (Supplementary Note 1 and Supplementary Fig. 4).

For phenotypic distinctiveness, the pie charts in Fig. 3a show the distribution of Target2–compounds retrieved by different methods (mAP ≥ 0.75), enabling comparison of SSL methods against CellProfiler within each source. Unlike MAE, the top-performing SSL models (DINO, DINOv2 and iTPN) show a high degree of agreement with CellProfiler. These models significantly retrieved more compounds (*P* ≤ 0.001), exceeding the capabilities of CellProfiler (Fig. 3e). DINO and DINOv2 showed the most robust performance across all sources, whereas iTPN showed a weaker performance for source 3 and source 6. The overall comparative performance at different mAP thresholds (Fig. 3b) further reflects the stability of the top-performing SSL models. The underperformance of MAE could be attributed to the scale of pretraining data, which may benefit from substantially larger datasets^27^.

The overlap of compounds retrieved by different SSL models is shown in Fig. 3c,d. The total retrieved Target2–compounds (mAP ≥ 0.75) ranked as follows: DINOv2 (129 compounds, 42.7% of Target2–compounds), DINO (128 compounds, 42.4%), iTPN (107 compounds, 35.4%) and MAE (53 compounds, 17.5%). About 96% (51 of 53) of MAE-retrieved compounds were also retrieved by the other three SSL methods, with no unique MAE retrievals. DINO and DINOv2 overlapped with 96% (103 of 107) of iTPN-retrieved compounds and additionally retrieved 22 compounds missed by masked-based models. When benchmarked against CellProfiler (Fig. 3d; 90 compounds, 29.8%), DINO and DINOv2 covered 96.6% (87 of 90) of CellProfiler retrievals, while iTPN covered 91% (Supplementary Fig. 5). The DINO and DINOv2 approaches show significantly broader coverage of retrieved replicates compared with CellProfiler and masked-based models (*P* ≤ 0.001; Supplementary Fig. 5b,c). The consistency between DINO and DINOv2 indicates that DINOv2 preserved the core strengths of DINO while improving biological relevance, as evidenced by the retrieval of additional gene targets such as *JAK1* and *MAPK14* (Fig. 2d) and reproducibility performance (Supplementary Table 3).

We further validated these findings on JUMP–Target2 positive controls, which include three categories^6^: compounds that correlate strongly with overexpression genes (poscon_orf, 6 compounds), selective compounds with highly confident gene target annotations (poscon_cp, 26 compounds) and pairs of compounds that are strongly correlated with each other but weakly correlated with other poscon_diverse compounds (poscon_diverse, 14 compounds). The top-performing SSL models (iTPN, DINO and DINOv2) consistently outperformed CellProfiler on the phenotypic distinctiveness (PNSB) task across all categories (Supplementary Fig. 6a), with a significant difference observed for poscon_diverse between CellProfiler and DINO/DINOv2 (*P* ≤ 0.01; Supplementary Fig. 6b). As the PNSB criterion is sensitive to well-position effects (Methods), we additionally evaluated the more stringent perturbation-not-same-batch-and-well (PNSBW) criterion on the 14 poscon_diverse compounds, which controls for both experimental-batch and well-position confounders, further confirming the robustness of DINO and DINOv2 (Supplementary Table 5).

### Effectiveness of DINO and DINOv2 in capturing compound signatures across experimental batches

**Extended Data Fig. 1:**
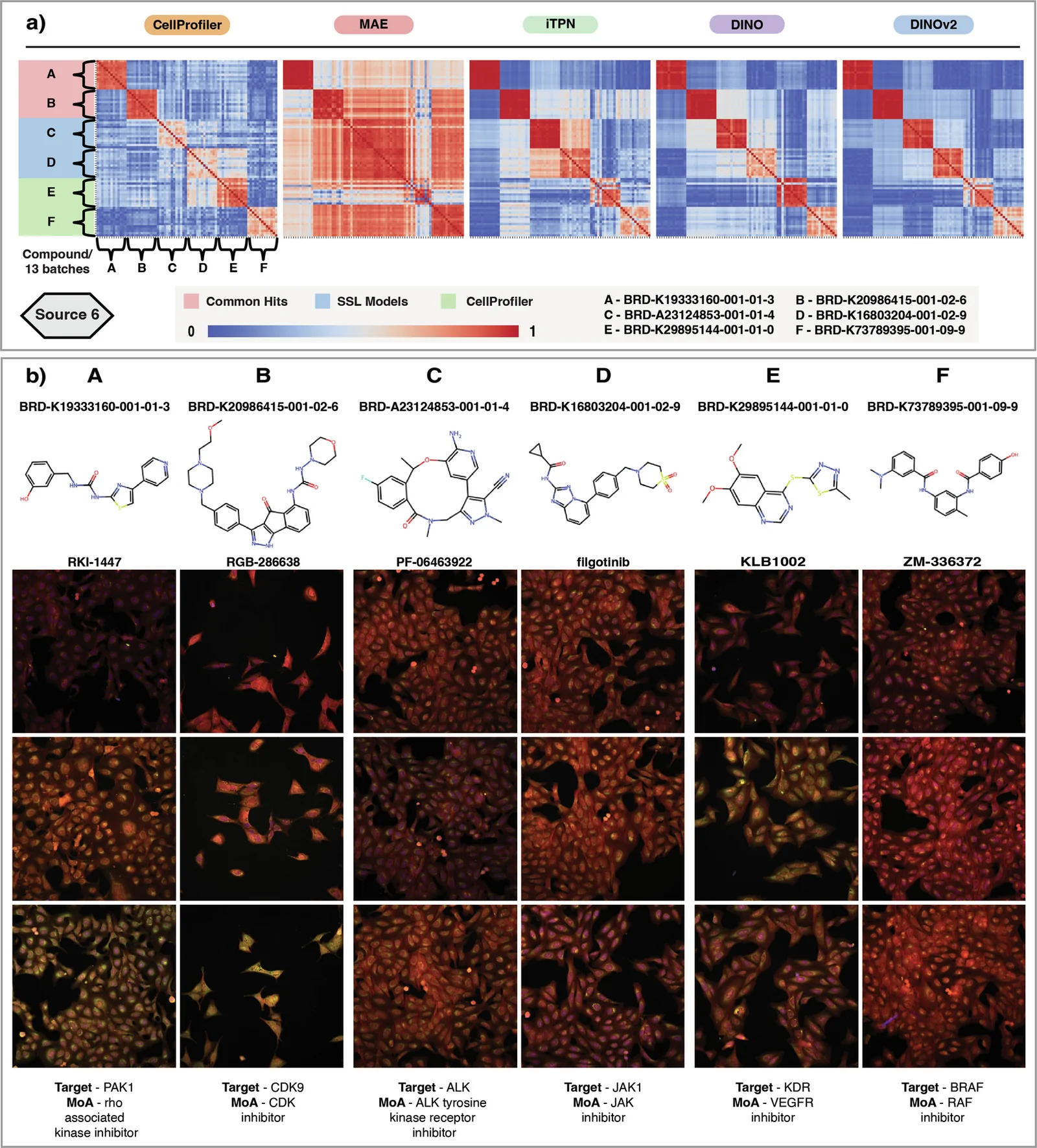
Comparative Performance of Different Methods in Learning Underlying Fingerprints of Selected Compounds. Comparative Performance of Different Methods in Learning Underlying Fingerprints of Selected Compounds: (**a**) heatmaps of batch-level pairwise correlation of representations learned from six compounds, each associated with a different gene target. These compounds were randomly selected based on the PNSB retrieval task (mAP ≥ 0.75) and categorized into three groups: common hits (compounds well learned by all methods, including SSL and CellProfiler), compounds better retrieved by SSL models, and compounds better retrieved by the CellProfiler approach. The heatmap illustrates that DINO and DINOv2 excel in capturing distinct compound-specific signatures, demonstrating their superior performance in learning the underlying fingerprints of these compounds. (**b**) Cell Painting images of these compounds (columns) across different batches (rows).

To gain further insight into the learned representations, we computed pairwise Pearson correlations across all experimental batches on the held-out source 6 for three categories of compounds (Extended Data Fig. 1). We randomly sampled two compounds per category, with each compound associated with a different gene target as shown in Extended Data Fig. 1b. In each category, the compounds were retrieved with mAP ≥ 0.75, and the categories were divided as follows: (1) compounds retrieved by all methods (that is, SSL and CellProfiler), (2) compounds better retrieved by SSL models and (3) compounds better retrieved by the CellProfiler approach. The heat maps reveal that DINO and DINOv2 captured stronger compound signatures compared with masked-based models and CellProfiler. Among masked-based models, iTPN showed notable improvements over MAE, which could be attributable to its hierarchical multi-scale ViT architecture (Extended Data Fig. 1a).

### Impact of pretraining strategies on model performance

**Extended Data Fig. 2:**
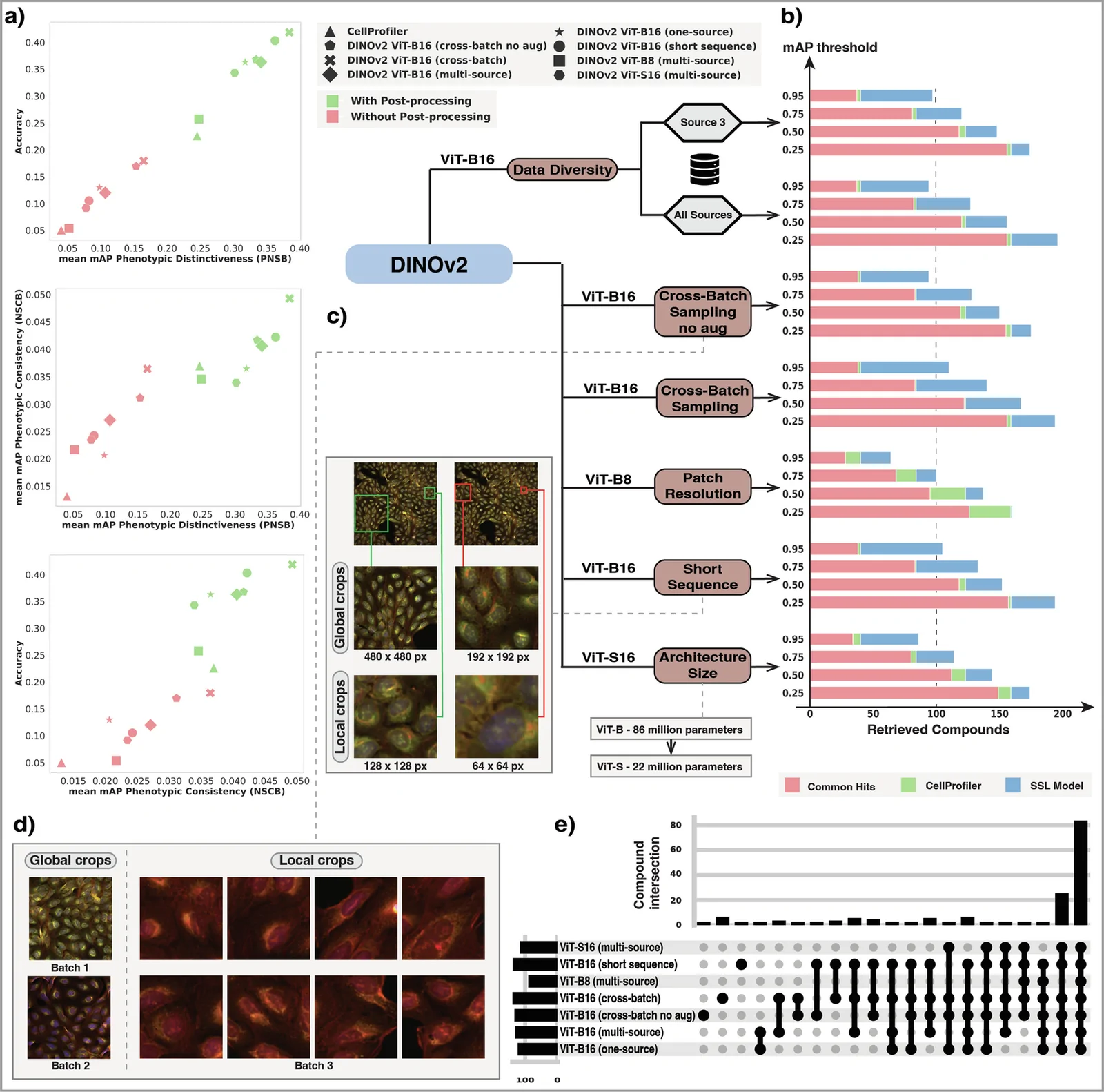
Evaluation of training strategies for DINOv2 and performance on retrieval tasks phenotypic distinctiveness (PNSB) and phenotypic consistency (NSCB) for Target2-Compounds. Evaluation of training strategies for DINOv2 and performance on retrieval tasks phenotypic distinctiveness (PNSB) and phenotypic consistency (NSCB) for Target2-Compounds (**a-c**). The different training strategies include data diversity, cross-batch sampling (**d**), resolution of patch tokens (that is, 8 × 8 vs 16 × 16), changing dimensions of global and local crop views (that is, patch-token sequence of different length), and varying architecture size for the vision transformer (that is, ViT-B16 vs ViT-S16). (**d**) Cross-batch sampling showing two global views sampled from different batches across-sources and eight local crops sampled from another different batch/source. Results reveal further performance gains achieved by incorporating data augmentation into the cross-batch sampling experiment. (**e**) Upset diagram showing the intersection of PSNB compounds retrieved by different models, reflecting overall homogeneity in retrieval performance.

In high-content image analysis, there has been a particular lack of ablation studies assessing the impact of various training strategies on the performance of the hybrid DINOv2 model, unlike the previously studied contrastive-based (that is, DINO^32^) and masked-based models^27^. Our evaluation, presented in Extended Data Fig. 2, covers several training conditions including data diversity, cross-batch sampling, patch token resolution, short sequence (that is, smaller input image size) and architecture size. Across different training strategies, DINOv2 consistently outperformed CellProfiler on retrieval accuracy (Extended Data Fig. 2a). Models pretrained on multi-source data outperformed those pretrained on a single source, underscoring the importance of data diversity in enriching FMs (Extended Data Fig. 2a,b and Supplementary Tables 3 and 4). Cross-batch sampling provides image crops with varied intensity distributions for the same perturbation (Extended Data Fig. 2d), which may reduce the need for additional data augmentation, while maintaining performance comparable to other models trained with the proposed augmentation techniques (Extended Data Fig. 2a). However, applying data augmentation together with cross-batch sampling further improved the overall model performance for both reproducibility and biological relevance tasks (Extended Data Fig. 2, Table 1, Supplementary Tables 3 and 4 and Supplementary Fig. 7). In the patch-token resolution experiment, the model trained with higher token resolution (ViT-B8) performed the lowest, probably owing to the quadratic complexity of the self-attention mechanism, which increases patch token data complexity and vulnerability to overfitting^34^. This suggests that higher-resolution models require further optimization or more extensive datasets to achieve better performance^27^. These findings provide valuable insights into the design and training of robust SSL models for HCI applications, highlighting the critical roles of data diversity and effective sampling techniques. In the data diversity experiments (Extended Data Fig. 2a,b), the datasets differ primarily in two aspects: (1) the total volume of images and (2) chemical diversity. To control for dataset size effect, we sampled all nine fields of view per well from the single-source dataset, whereas only one field of view (FOV) per well was randomly sampled from the multi-source dataset. Specifically, the single-source dataset includes 591,101 data points (each representing a 5-channel image) from all unannotated perturbations in source 3 (58,662 compounds), with full well sampling. By contrast, the multi-source dataset comprises 353,607 data points from 7 sources (119,876 compounds), with a single sampled FOV per well. Notably, the multi-source dataset contains approximately twice as many compounds, reflecting greater chemical diversity, while the total number of images in the single-source dataset is about 1.7 times larger. Despite this, the model performed better with the multi-source data, suggesting that chemical diversity has a more substantial impact on model robustness than sheer image volume alone.

In addition to evaluating the standard ViT-B16 architecture, we also explored the performance of a smaller architecture, ViT-S16, which has fewer parameters and reduced complexity. While ViT-S16 offers a more computationally efficient alternative, our results indicate that ViT-B16 outperforms ViT-S16 in both reproducibility and biological relevance tasks (Extended Data Fig. 2a,b, Table 1 and Supplementary Tables 3 and 4). This suggests that the additional parameters and complexity of ViT-B16 contribute to its superior performance in capturing biologically relevant features and ensuring consistent results across different experiments. Therefore, while ViT-S16 may be suitable for scenarios with limited computational resources, ViT-B16 remains the preferred choice for high-content image analysis tasks requiring high accuracy and reliability, particularly at the data scale presented in this study. Additionally, we compared the backbone architecture of ViT-B16 against a Swin Transformer V2 Base (window size 16)^55,56^ on the phenotypic distinctiveness task. ViT-B16 achieved superior performance after postprocessing (100% retrieval, 42.27% mAP) compared with SwinV2-B-W16 (94.12% retrieval, 29.45% mAP), suggesting that ViT’s capacity for modeling long-range dependencies provides a more robust feature representation (Supplementary Note 2, Supplementary Fig. 8 and Supplementary Table 11).

The computational complexities of training different SSL models vary owing to differences in their underlying optimization processes. DINOv2 incorporates FlashAttention^57^ and optimized training strategies^29^, making it 1.7 times faster than DINO on the same JUMP–CP training set. Masked-based methods achieve more efficient training by processing only unmasked input portions, with MAE being 1.35 times faster than iTPN (1,022.4 compared with 1,382.4 GPU hours; GPU, graphics processing unit). Despite these training differences, inference time is fast across all models (~1.5 min per plate; Supplementary Table 6).

The impact of various training strategies led to changes in the overall performance of downstream tasks for both reproducibility and biological relevance compared with the baseline model of DINOv2 ViT-B16 (multi-source). Among these strategies, the cross-batch sampling with augmentation achieved the best overall performance (Extended Data Fig. 2). Although differences among training strategies were observed, they were generally not statistically significant (Supplementary Fig. 9), particularly at the higher reproducibility retrieval threshold (mAP ≥ 0.75) for the JUMP–Target2–compounds. The exception was the DINOv2 model trained with a higher token resolution (ViT-B8) and a longer token sequence, which showed significantly lower performance (Supplementary Fig. 9). The ViT-B8 model’s underperformance is further explained by optimization challenges arising from the quadratic increase in token-sequence length (900–3,600 tokens; Supplementary Note 2 and Supplementary Fig. 10). These ablation analyses provide valuable insights into the design and training of robust SSL models, emphasizing the importance of data diversity, effective sampling strategies and appropriate token resolution.

### Revealing cellular semantics: insights from DINO and DINOv2 local representations

**Extended Data Fig. 3:**
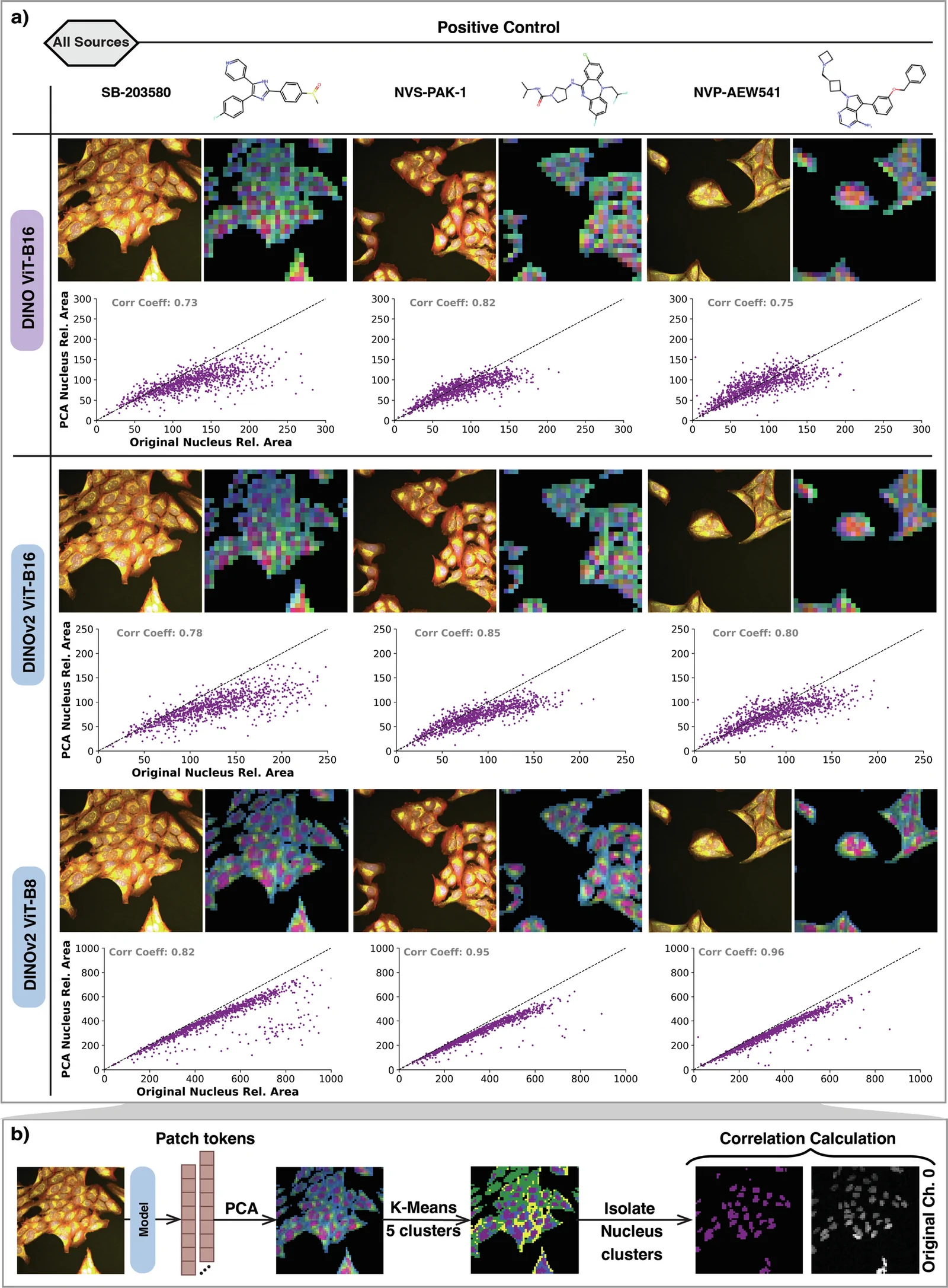
Analysis of local representations of DINO and DINOv2 models reveal cellular semantics. Analysis of local representations of DINO and DINOv2 models reveal cellular semantics: (**a**) original image crops and spatially-mapped PCA projections of high-dimensional patch tokens into a three-dimensional space, with PCA coordinates mapped to form an RGB image, revealing similar colors for similar local representations. (**b**) Summarized steps for cell nucleus segmentation based on the analysis of SSL-derived patch tokens. The scatter plots show a strong correlation between the original and SSL-derived nucleus segmentation (**a**), with a total of 2980 images analyzed for each treatment (columns) across all sources and experimental batches.

To understand the cellular semantics captured by knowledge distillation-based models of DINO and DINOv2, we analyzed their local representations (patch tokens) by projecting them into the principal component analysis (PCA) space and spatially mapping the coordinates to form RGB images (Extended Data Fig. 3 and Methods). This analysis was conducted across 3 well-retrieved positive-control drug conditions: SB-203580, NVS-PAK-1 and NVP-AEW541, using 3 model variants (DINO ViT-B16, DINOv2 ViT-B16, DINOv2 ViT-B8), totaling 2,980 high-content images from all available sources and batches. The analysis revealed that cellular structures with similar local representations cluster together, reflecting semantics captured through self-supervised knowledge distillation. Owing to the absence of ground-truth segmentation masks for cellular compartments in JUMP–CP, which would also require more advanced segmentation pipelines^58^, we focused on the cell nucleus as a readily extractable structure to quantify semantic insights. Therefore, the quantification was based on correlating nucleus areas extracted via *k*-means clustering of patch tokens with ground-truth nucleus areas obtained from DNA-channel thresholding (Extended Data Fig. 3b and Methods). Strong positive Pearson correlations were observed, particularly for DINOv2, where coefficients consistently exceeded 0.8 (Extended Data Fig. 3a). DINOv2 ViT-B8 achieved the highest correlations, showing that finer patch resolution captures more detailed cellular structures (Extended Data Fig. 3a and Supplementary Figs. 11 and 12). These findings highlight the interpretability of SSL local representations, showing that knowledge distillation-based models capture spatially resolved cellular semantics without supervision. This could support efforts to better understand cellular responses to perturbations^59,60^ and develop spatially informed biomarkers for drug response monitoring^61–63^. For masked-based models, reconstruction quality serves as the key indicator of effectiveness; we incorporated a Fourier-domain reconstruction loss into iTPN, which has previously shown improved data reconstruction and model convergence in MAE^27^ (Supplementary Note 3 and Supplementary Fig. 13).

### Biological relevance of FMs on EU-OPENSCREEN bioactive compounds: generalizability on unseen compounds and HepG2 cells

We assessed model generalizability through the biological relevance of the learned representations using the phenotypic consistency (NSCB) task on 2,464 densely annotated bioactive compounds from the EU‑OPENSCREEN dataset^53^. Images were acquired at four independent sites (FMP, MEDINA, IMTM and USC). All sites generated four‑channel high‑content images of HepG2 cells, and the FMP site additionally profiled U2OS cells. The pretrained FMs were applied to this dataset in a zero-shot setting, without any fine-tuning. We evaluated biological relevance across multiple annotation levels—pathways, MoAs and protein targets—by computing phenotypic consistency within shared annotations at each site independently and then consolidating results to enable cross-site comparison. Because most compounds are annotated with multiple targets, phenotypic consistency was evaluated using the multilabel setting implemented in the open-source Python package copairs^64^. Before biological relevance task evaluation, we filtered compounds to exclude toxic compounds—defined as compounds reducing the cell number below 2.5 standard deviations of the dataset median^53^—and retained only those showing significant phenotypic activity based on our phenotypic activity task (Supplementary Table 7). Consistent with earlier findings, Table 2 shows that DINO and DINOv2 consistently outperformed both the CellProfiler baseline and masked-based models across biological annotation levels in both U2OS and HepG2 cells.

**Table 2:** Evaluation of multi-level biological relevance using the phenotypic consistency (NSCB) task on the EU-OPENSCREEN cpg0036 dataset (2,464 compounds)

		Cell types (retrieved (%), mAP (%))				
	Site	FMP		IMTM	MEDINA	USC
	Model	U2OS	HepG2	HepG2	HepG2	HepG2
Target	CellProfiler	10.71, 2.26	13.08, 1.62	16.82, 3.78	16.44, 2.36	17.32, 1.71
	MAE ViT-B16	0.00, 0.86	0.00, 3.11	9.02, 4.49	12.50, 3.07	11.97, 1.13
	iTPN HiViT-B16	0.00, 0.72	0.31, 0.97	17.92, 3.68	16.41, 2.04	20.04, 1.31
	DINO ViT-B16	23.43, 3.62	23.18, 2.34	19.27, 4.09	27.77, 3.32	28.03, 2.67
	DINOv2 ViT-B16	24.72, 3.98	20.13, 2.09	19.28, 4.17	27.17, 3.16	26.99, 2.50
MoA	CellProfiler	7.21, 2.05	9.18, 1.68	10.61, 3.61	14.52, 2.80	11.11, 1.89
	MAE ViT-B16	0.00, 0.77	0.00, 1.72	2.86, 4.42	10.23, 2.92	2.41, 0.88
	iTPN HiViT-B16	0.00, 0.55	0.00, 0.73	6.40, 2.85	12.96, 1.82	9.64, 1.07
	DINO ViT-B16	15.03, 3.57	12.23, 2.20	12.95, 4.35	23.24, 3.79	17.33, 2.65
	DINOv2 ViT-B16	12.76, 3.68	9.79, 1.86	12.12, 4.00	21.16, 3.78	19.70, 2.49
Pathway reactome level 3	CellProfiler	30.58, 5.86	37.44, 5.99	51.28, 7.41	39.11, 7.38	35.96, 6.26
	MAE ViT-B16	3.30, 5.69	3.42, 9.85	48.28, 9.82	49.08, 11.47	34.90, 6.17
	iTPN HiViT-B16	4.46, 5.01	16.93, 5.85	56.61, 8.19	47.64, 8.13	46.00, 6.18
	DINO ViT-B16	57.21, 8.08	54.55, 7.32	58.29, 7.55	59.50, 8.16	61.58, 6.99
	DINOv2 ViT-B16	55.22, 8.25	51.50, 7.13	55.78, 7.36	59.69, 8.21	60.78, 6.99
Pathway reactome level 5	CellProfiler	39.29, 5.18	44.00, 4.65	57.47, 6.09	52.97, 5.85	52.89, 4.86
	MAE ViT-B16	1.51, 3.80	0.80, 6.88	41.75, 7.27	51.35, 8.41	45.33, 4.32
	iTPN HiViT-B16	3.18, 3.44	10.95, 4.08	58.88, 6.56	56.48, 6.11	56.50, 4.38
	DINO ViT-B16	67.71, 6.91	63.96, 5.74	64.38, 6.65	69.68, 6.67	72.65, 5.66
	DINOv2 ViT-B16	67.12, 7.12	63.56, 5.47	63.06, 6.64	66.82, 6.59	75.00, 5.52

Compounds were filtered to exclude toxic profiles and to retain those with significant phenotypic activity as determined by the phenotypic activity task (Supplementary Table 8). The remaining compounds were evaluated for phenotypic consistency across different biological levels, including pathway, MoA and protein-target annotations. Compared with baseline and masked-based methods, DINO and DINOv2 showed consistently higher performance across biological levels, cell types (U2OS and HepG2) and data acquired from different laboratories.

**Fig. 4:**
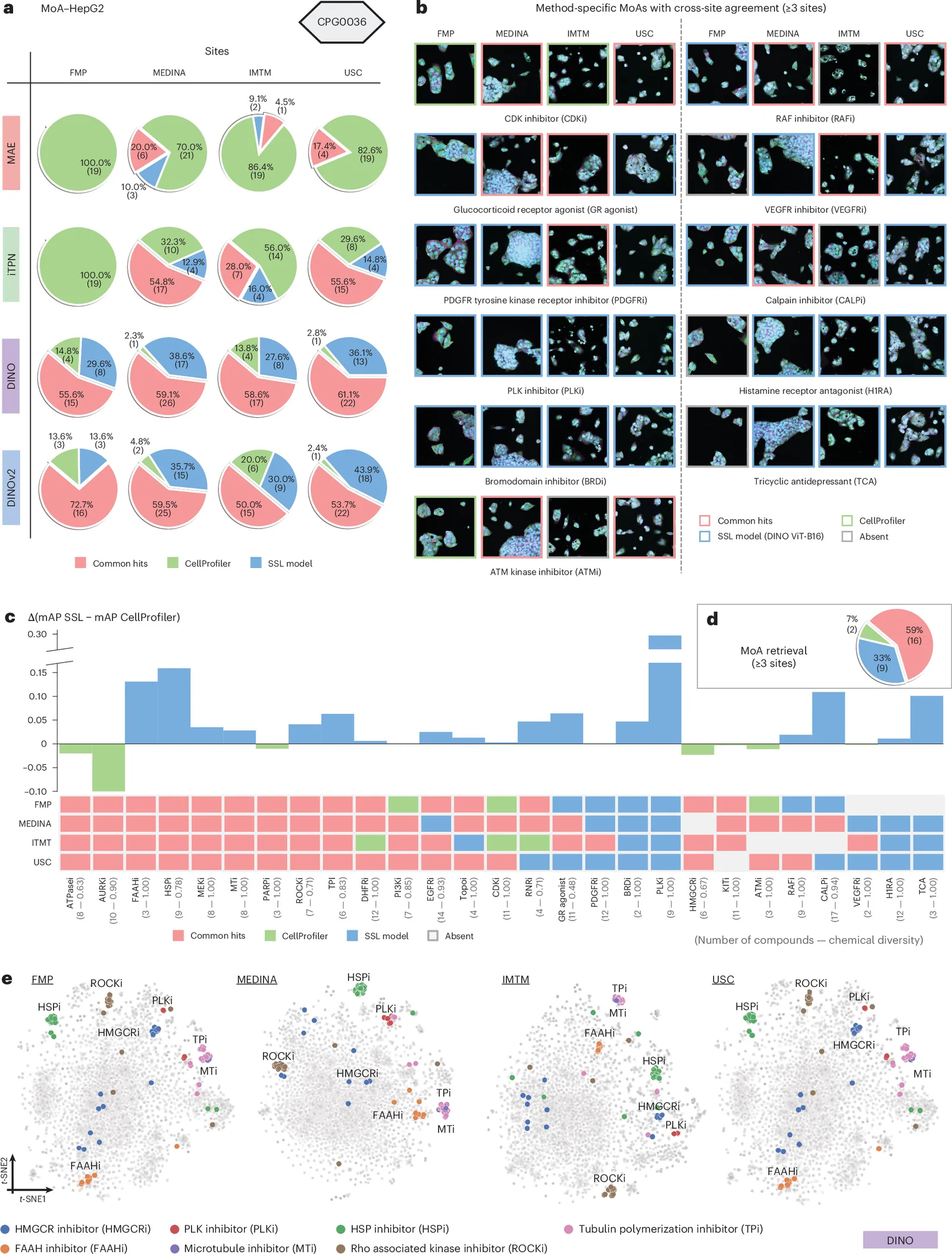
Evaluation of phenotypic consistency (NSCB) for MoA retrieval across four screening sites (FMP, MEDINA, IMTM, USC) in HepG2 cells. **a**, Site-wise comparison of CellProfiler and SSL models showing the number of MoAs significantly retrieved by each method (corrected *P* value < 0.05). **b**, Representative composite 4-channel cell painting images for MoAs uniquely retrieved by CellProfiler or SSL models with cross-site agreement (≥3 sites). **c**,**d**, Comparison between CellProfiler and the top-performing SSL model, DINO (**c**), summarizing cross‑site agreement in retrieved MoAs (**d**). Overall, 59% of MoAs were jointly identified by both methods. DINO retrieved 33% more MoAs than CellProfiler, including polo-like kinase (PLK) and bromodomain inhibitors consistently detected across all four sites. DINO also showed higher overall MoA retrieval performance as measured by mAP (Supplementary Table 13). Compound counts and chemical diversity per MoA (Murcko scaffolds and compounds) are indicated in **c**. **e**, *t*-SNE visualization of normalized, plate-aggregated DINO features for MoAs retrieved (mAP ≥ 0.2) revealing consistent phenotypic clustering patterns across sites.

At the MoA annotation level in HepG2 cells, site-wise comparison of CellProfiler against each SSL model showed that masked-based models underperformed CellProfiler, whereas DINO and DINOv2 consistently outperformed CellProfiler, enabling retrieval of more MoAs (Fig. 4a). We further evaluated phenotypic consistency performance for cross-site agreement (MoAs retrieved in at least three sites) (Fig. 4b–d). DINO recovered 89% (16 of 18) of the cross-site MoAs identified by CellProfiler; CDK and ATM kinase inhibitors were retrieved with cross-site agreement only by CellProfiler (Fig. 4c,d). In addition, DINO retrieved 33% more MoAs than CellProfiler with cross-site agreement and achieved higher overall MoA retrieval performance as measured by mAP (Fig. 4c and Supplementary Table 13). Notably, DINO uniquely retrieved polo-like kinase and bromodomain inhibitors across all 4 sites, with mAP scores of 0.326 and 0.076, respectively, compared with 0.028 and 0.027 for CellProfiler. Representative composite four-channel cell painting images for MoAs uniquely retrieved by CellProfiler or SSL models with cross-site agreement are shown in Fig. 4b. Compound counts and chemical diversity per MoA (Murcko scaffolds and compounds) are indicated in Fig. 4c. Low-dimensional visualization of normalized, plate-aggregated DINO features for MoAs retrieved with mAP ≥ 0.2 revealed consistent phenotypic clustering patterns across sites (Fig. 4e). These results show that DINO representations capture biologically meaningful and mechanistically coherent signals that generalize across laboratories.

### U2OS pretrained FMs show robustness and generalizability on MCF-7 cells: MoA classification of BBBC021

**Extended Data Fig. 4:**
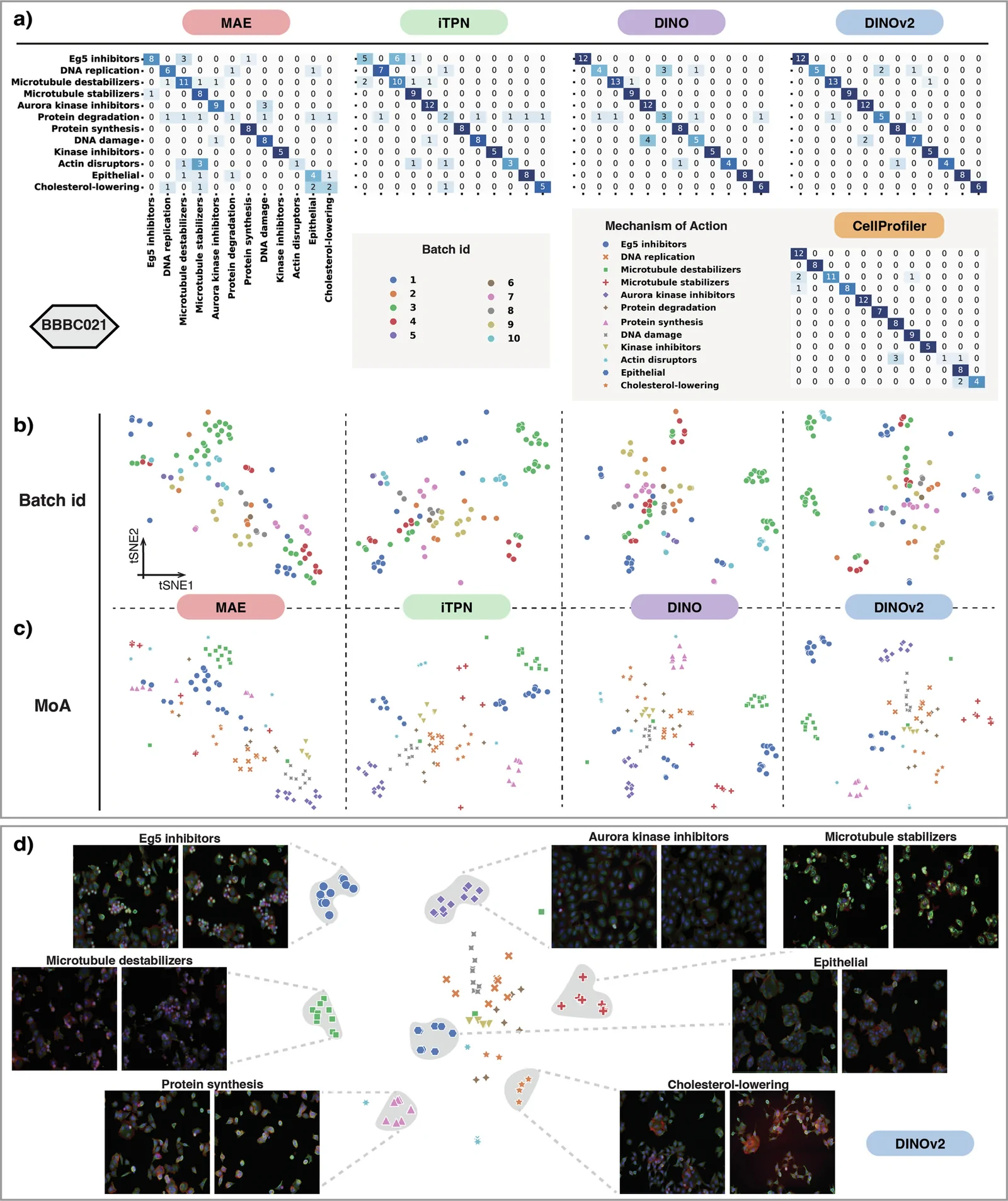
Comparative performance of various models on the sphering-normalized representation from BBBC021 dataset. Comparative performance of various models on the sphering-normalized representation from BBBC021 dataset: (**a**) Confusion matrix for the K-Nearest Neighbor (k = 1) prediction of MoAs for the Not-Same-Compound (NSC) evaluation. The CellProfiler confusion matrix was adopted based on results reported by Singh et al^65^ (**b-c**) t-SNE visualization of treatment-level aggregated average embeddings extracted from various foundation models and mapped from the original 768 dimensions into 2 dimensions. The t-SNE maps show clustering of similar MoAs (**c**) across different batches (**b**), reflecting the robustness of the learned representation in capturing biological signals. (**d**) Example of visual distribution of high-content images from well-predicted MoAs based on DINOv2 representation.

To demonstrate generalizability, we applied the pretrained FMs directly to the BBBC021 dataset, which features MCF-7 breast cancer cells imaged with three fluorescent channels—one of which (B-tubulin) was not present during pretraining. The models were applied in a locked state without fine-tuning, and extracted embeddings were normalized for batch effects before downstream analysis (Methods). Using a few-shot *k*-NN classifier (*k* = 1) under the not-same-compound (NSC) evaluation scheme, DINOv2 achieved the best overall NSC accuracy of 91.3%, followed by CellProfiler (90%)^65^, DINO (86.4%), iTPN (79.6%) and MAE (68.9%) (Extended Data Fig. 4a and Supplementary Table 8). The *t*-SNE maps of treatment-level averaged embeddings show clustering of similar MoAs across experimental batches in a zero-shot manner (Extended Data Fig. 4b–d). DINOv2 outperformed CellProfiler in NSC accuracy, while iTPN led in the other metric of NSCB accuracy across different *k* values (Supplementary Table 9).

Per-MoA analysis revealed that CellProfiler showed substantially lower accuracy for actin disruptors (20%) compared with 80% for DINO and DINOv2, whereas CellProfiler achieved superior performance on DNA replication (100%) relative to all SSL methods (Extended Data Fig. 4a). DINO and DINOv2 predicted 7 MoAs with 100% accuracy, compared with 5 for iTPN and 2 for MAE. For example, unlike masked-based models, Eg5 inhibitors were only well predicted by DINO and DINOv2. Conversely, masked-based models showed slightly better performance than DINO and DINOv2 in predicting DNA replication. Although DINO performed strongly overall, DINOv2 slightly outperformed DINO in predicting protein degradation and DNA damage. Notably, DINOv2 showed agreement with masked-based models in better predicting DNA damage, reflecting the benefit of its hybrid learning mechanism that integrates both contrastive and masked-based objectives. The PCA-based analysis of local representations further confirmed that DINO and DINOv2 models capture meaningful cellular semantics on this dataset (Supplementary Figs. 14 and 15). These results underscore the consistent robustness of the pretrained DINO and DINOv2 models, compared with masked-based models, in generalizing to unseen cell types.

### Generalization of FMs to RxRx1 dataset: genetic perturbations on diverse cell types

To further evaluate generalizability, we applied the pretrained FMs in a zero-shot manner to the RxRx1 dataset^52^, which includes 1,138 gene perturbations in 51 experimental batches across 4 cell types: HepG2, HUVEC, RPE and U2OS. Embeddings were assessed for phenotypic activity and phenotypic distinctiveness (PNSBW). Our results show that FMs pretrained on U2OS cells generalize effectively to three additional cell types with markedly different morphologies (Extended Data Table 2). Postprocessing substantially improved performance across all models and cell types across both metrics of phenotypic activity and distinctiveness (Extended Data Table 2 and Supplementary Table 10). Because RxRx1 includes only one DMSO well per plate, plate-wise MAD normalization to DMSO was unreliable for processing classical baseline features. We therefore adopted the same postprocessing pipeline used for SSL features (sphering–MAD–Harmony), in which sphering is performed using all DMSO wells across batches, followed by whole-plate MAD normalization and Harmony batch correction (Methods). For example, in HepG2 cells, DINOv2 achieved a mAP phenotypic activity score of 27.64%, which increased to 65.63% after postprocessing, representing an improvement of over 137% (Supplementary Table 10).

Compared with the classical baseline and masked-based models, DINO and DINOv2 consistently showed higher phenotypic distinctiveness (PNSBW) across all cell types (Extended Data Table 2), with DINOv2 attaining the highest retrieved scores (≥95%). Visualization of 30 positive-control compounds confirmed that DINO and DINOv2 achieved better phenotypic separation as quantitatively reflected by substantially higher silhouette and graph connectivity scores compared with MAE and iTPN (Supplementary Figs. 16 and 17). Of note, we did not filter compounds based on their phenotypic activities before the phenotypic distinctiveness task. This ensures unbiased comparison across SSL methods, as different models may identify different subsets of active compounds, which would otherwise lead to unequal sample sizes in the distinctiveness evaluation. These results underscore the generalizability of DINO and DINOv2 in capturing meaningful biological signals from genetic perturbations across diverse cell types.

## Discussion

This study presents a large-scale systematic benchmarking of SSL FMs for morphological profiling, pretrained on the JUMP–CP dataset. Our findings show that different SSL learning mechanisms yield representations with distinct strengths across downstream tasks, and the pretrained FMs show generalizability to unseen perturbations and cell types. We publicly release these pretrained FMs as deep feature extractors that bypass cell segmentation and feature engineering, to facilitate adoption by the morphological profiling community.

SSL-based modeling offers fundamental advantages over classical morphological profiling methods by avoiding the dependence on cell segmentation and feature engineering^5,13^, thereby enhancing the robustness and scalability needed for large-scale profiling^27^. Cell segmentation requires iterative processing^66^ that can be particularly challenging for complex cell types, such as neuronal cells^67^, and feature engineering can introduce source-specific bias, as evidenced by CellProfiler’s suboptimal performance on source 8 (Supplementary Note 1). By contrast, SSL methods show consistent robustness across all sources without additional feature selection. Despite these advantages, and unlike masked-based models, contrastive and hybrid approaches rely on data augmentation for learning objectives, which could impact representation quality. This limitation can be mitigated through cross-batch sampling, a domain adaptation strategy incorporated into the learning process that leverages experimental metadata to sample views of the same perturbation across different batches^44,45^. While these U2OS pretrained FMs, particularly DINO and DINOv2, showed strong generalizability in zero-shot and few-shot settings, future efforts could investigate FM fine-tuning strategies^68^ to further optimize adoption across additional cell types.

Despite the strong performance of FMs, batch effects remain a challenging problem in cell painting^54^, especially when aligning data from different laboratories such as those provided by JUMP–CP^6^. Our ablation analysis covered widely adopted batch-correction methods within the image-profiling field^9,54,69^, as well as methods from other domains such as RNA sequencing^70^. Notably, Harmony, originally developed for single-cell RNA sequencing, has recently been reported as a promising approach for image-based profiling^54^. The ordered sequential combination of sphering, MAD normalization and Harmony applied to SSL features achieved the highest scores for phenotypic distinctiveness and phenotypic consistency tasks. Nevertheless, an inherent limitation of sphering and Harmony is their inability to adapt precomputed transforms to align new batches, necessitating reprocessing of all data. While within-source batch correction substantially improved retrieval performance, aligning data across different sources proved more challenging (Supplementary Fig. 3), underscoring the need for advanced batch-correction methods capable of handling complex inter-laboratory variations. One potential direction involves incorporating previous knowledge to regularize the pretraining phase, extending the cross-batch sampling approach^44^ with cost functions that account for source-level variations.

While classical morphological profiling methods such as CellProfiler offer greater potential for feature interpretability^71^, deep learning-based methods often lack interpretability. This is particularly relevant in drug discovery, where understanding model decisions is crucial for ensuring reliability and safety of potential treatments^72^. Recent works such as gradient-weighted class activation mapping overlap (Grad-CAMO)^73^ aim to measure model attention on relevant biological features, and CytoSummaryNet^74^ uses SSL within a multiple-instance learning framework to enhance interpretability of single-cell morphological profiles. DINO and DINOv2 show promise in this direction by focusing on relevant cellular features and learning rich local representations without manual annotations, effectively supporting the study of biological variations including single-cell heterogeneity^46^. Despite these advancements, interpretability remains a notable challenge, and continued innovation is needed to achieve more interpretable and trustworthy systems in complex biological contexts^4,72^ (Supplementary Note 4).

The current advances in representation learning offered by FMs hold potential for enabling complex applications, such as matching genetic and chemical perturbations^1,75^. The JUMP–CP dataset provides a unique opportunity as a public image-based resource with annotated pairs of genetic and chemical perturbations. This dataset includes imaging-based CRISPR–Cas9 knockout and gene overexpression data, which facilitates the comparison of mechanisms underlying perturbed genetic pathways and can lead to the discovery of MoAs. The availability of paired genetic and chemical perturbations in JUMP–CP paves the way for future research to develop multi-modal FMs optimized for learning joint representations or novel fusion strategies between these modalities^76^. Recent efforts have shown performance gains with multi-modal approaches integrating representations from high-content images and chemical structures^9,77,78^. Moreover, multi-modal learning approaches supported by robust batch-correction methods^79^ can better capture subtle biological variations and underlying heterogeneity, enhancing our understanding of the complex interactions between genetic and chemical perturbations.

In conclusion, this study provides a large-scale systematic benchmark across major SSL learning mechanisms and offers pretrained FMs as a scalable and robust alternative to classical pipelines for morphological profiling. However, limitations remain. Our FMs were pretrained exclusively on U2OS cells with chemical perturbations; although they generalized to four additional cell types in a zero-shot setting, more complex cell types such as neurons may require fine-tuning. While initial generalization to genetic perturbations was shown (RxRx1), comprehensive evaluation on large-scale genetically perturbed datasets, such as the CRISPR and overexpression data in JUMP–CP, remains to be conducted. Moreover, cross-source batch effect correction remains challenging and deep learning features are less interpretable than engineered ones.‌ The demonstrated generalizability across laboratories, multiple cell types and perturbation modalities provides a basis for future expansion by incorporating multi-modal FMs, regularized with batch effect mitigation strategies, to unlock biological insights from complex applications such as matching genetic and chemical perturbations across different laboratories.

## Methods

### Datasets

#### JUMP–CP dataset

We used the largest publicly available cell painting dataset generated by the JUMP–CP consortium^6^. Specifically, our FMs were pretrained and evaluated on the principal JUMP–CP dataset of cpg0016, available from the Cell Painting Gallery^80^ on the Registry of Open Data on AWS (https://registry.opendata.aws/cellpainting-gallery/). These data were downloaded to our company servers and accessed based on the available updates as of February 2023. The dataset includes morphological profiles and approximately 5.8 million 5-channel HCI of human U2OS osteosarcoma cells, acquired at different labs (‘sources’) and treated with around 136,000 diverse chemical and genetic perturbations. The five-channel images are based on the standard cell painting assay protocol^3^ that reveals the following cellular compartments: nucleus (DNA), mitochondria, nucleoli and cytoplasmic RNA, endoplasmic reticulum, Golgi and plasma membrane, and the actin cytoskeleton. The images were acquired at 20× resolution using 5 different microscopic systems available at various sources. All the images are available in 16-bit format with spatial dimensions varying between sources, ranging from 970 × 970 to 1,080 × 1,280 pixels. There were a total of 10 sources; however, we had to exclude two sources from our analysis, source 1 and source 9, as they did not offer any HCI data at that time. All the remaining sources were used to train and validate our models.

#### JUMP–CP data split

We divided the dataset into training and evaluation sets. The training set consists of all unannotated chemical perturbations, totaling 119,876 compounds (353,607 wells from 1,364 plates and 87 experimental batches), sampled from all sources except source 6, which was held out entirely for evaluation. In this study, ‘batch’ refers to experimental batches generated within the same laboratory, totaling 87 across sources, unless otherwise specified. To accelerate FM training while maintaining data diversity, we randomly sampled one FOV per well, resulting in ~1.77 million microscopic images. The evaluation set comprises three groups: (1) source 6, (2) controls and (3) JUMP–Target2–compounds, which annotates 302 compounds to 160 gene targets from all 8 sources across 98 experimental batches. Extended Data Table 1 shows summary statistics on data distribution.

#### EU-OPENSCREEN Bioactives dataset (cpg0036)

To further show and benchmark the biological utility of our FMs, we evaluated downstream biological relevance tasks using the public EU-OPENSCREEN Bioactives cell painting dataset^53^. This dataset holds 2,464 curated and well-annotated bioactive small molecule compounds across 4 imaging sites (FMP, IMTM, MEDINA and USC) and 2 cell lines (HepG2 and U2OS) under a standardized, cross-site-optimized cell painting workflow. Supplementary Table 12 shows summary statistics on data distribution across sites. This densely annotated collection includes protein-target annotations for 96% of compounds, covering 2,841 targets in total; and compounds are typically multi-target, with a median of 6 targets per compound. The screen used 10 µM dosing with plate-level DMSO controls, and images were acquired on high-content confocal microscopes using a 4-channel staining panel (nucleus, endoplasmic reticulum, actin and mitochondria). Tetrandrine and nocodazole at 5 µM concentration served as positive controls. The assay was run in 4 replicates to assess robustness across experimental batches, and the collection comprises 483,795 four-channel images. The dataset provides annotations across biological scales—including pathways (for example, Reactome), MoAs and protein targets—and, together with associated materials (raw images, metadata and processed profiles), is publicly accessible via the Cell Painting Gallery (accession cpg0036-EU-OS-bioactives). For more information on this dataset, we refer to ref. ^53^

#### BBBC021 dataset

The capabilities of our FMs to generalize on different cell types were validated using the BBBC021v1 dataset^50^, available from the Broad Bioimage Benchmark Collection^51^. This dataset contains 16-bit three-channel images (DNA, F-actin, B-tubulin) of MCF-7 p53-wildtype breast cancer cells treated with various chemical perturbations. The annotated subset includes 12 unique MoAs identified for 103 treatments (38 compounds at 1–7 concentrations each) across 10 experimental batches, comprising 1,320 DMSO control images and 2,528 MoA-annotated images. The annotated subset was downloaded using a freely available Python package (https://github.com/giacomodeodato/pybbbc).

#### RxRx1 dataset

The RxRx1 public dataset^52^ comprises 125,510 6-channel high-content images of 4 human cell types (HUVEC, RPE, HepG2 and U2OS) subjected to gene knockdown via small interfering RNA, totaling 1,138 treatments across 51 experimental batches. Images were generated using a standardized cell painting protocol highlighting six organelles: nucleus, endoplasmic reticulum, actin cytoskeleton, nucleolus, mitochondria and Golgi apparatus. Although the raw images are publicly available, neither CellProfiler-derived features nor a CellProfiler pipeline has been released for this dataset. To provide a benchmarking baseline, we extracted engineered morphological features using Acapella (PerkinElmer), a commercial high-content image analysis package. The analysis workflow was adapted from the JUMP–CP CellProfiler pipeline^6^ and included illumination correction followed by cell segmentation, extracting a total of 900 features capturing intensity, texture and geometric properties, with single-cell features averaged per well. Although this Acapella-based pipeline generalized well to three of the four cell types, HepG2 required additional segmentation optimization beyond the scope of this work; accordingly, baseline results are not reported for this cell type.

### Image preprocessing and crop sampling

All images were first preprocessed using channel-wise operations that involved intensity clipping at the 0.01st and 99.9th intensity percentiles, followed by min–max normalization to achieve a mean of 0 and a standard deviation of 1. To improve memory and computational complexities during training of the FM, the HCI training dataset, ${X}_{{tr}}=\left\{{x}_{{tr}}^{\left(1\right)},\,{x}_{{tr}}^{\left(2\right)},\ldots ,\,{x}_{{tr}}^{\left(N\right)}\right\},$ was converted into an 8-bit format. Nevertheless, the inference pipeline operated directly on the preprocessed 16-bit images. The FMs were trained on non-overlapping image crops (that is, views) sampled from the original image using a regular grid of predefined size. Additionally, Otsu’s thresholding was applied to the DNA channel to avoid sampling empty crops. Moreover, the annotated BBBC021 dataset was preprocessed using an illumination correction method with a Gaussian smoothing filter of 320 pixels, as previously defined^65^. For the RxRx1 dataset, we used only the first five channels, omitting the sixth channel (Alexa Fluor 555 Agglutinin fluorescent staining). This decision was informed by previous findings^52^, which showed that excluding the sixth channel improved perturbation classification accuracy by approximately 2% in baseline models, suggesting that it reduces noise and improves robustness of the extracted features.

### Model architecture

The backbone of our FMs is based on ViTs^35^. A high-content image $x\in {{\mathbb{R}}}^{H\times W\times C}$ is modeled as a sequence of smaller squared image patches ${S}_{p}=\{{x}_{p}^{\left(1\right)},{x}_{p}^{\left(2\right)},\ldots ,{x}_{p}^{\left(n\right)}\}$; ${x}_{p}\in {{\mathbb{R}}}^{P\times P\times C}$, where (*H*, |, *W*) and (*P*, |, *P*) are the spatial dimensions of the input image and each patch respectively, *C* is the number of channels and *n* is the sequence length provided as input to the transformer. We experimented with patch sizes of 8 × 8 pixels and 16 × 16 pixels. The FM maps an input image into a *k*-dimensional embedding space: ${FM}:{x}^{\left(i\right)}\in {{\mathbb{R}}}^{H\times W\times C}\to {z}^{\left(i\right)}\in {{\mathbb{R}}}^{k}$, where *z* is the learned feature representation. The embedding dimension is determined by the model size: a small vision transformer (ViT-S) has 22 million parameters with 384-dimensional embeddings, whereas a base model (ViT-B) has 86 million parameters with 768-dimensional embeddings. The optimized model provides both local patch embeddings (patch tokens) and a global image-level representation (class (CLS) token). Patch embeddings provide local representations for each patch image ${x}_{p}^{(i)}$ in the sequence *S*_*p*_, whereas global image-level representation is calculated based on attention-based aggregation from the sequence of patch embeddings. We used the global image-level representation for all downstream analyses. For more information about ViTs, we refer to ref. ^35^.

### Self-supervised learning

#### Contrastive-based learning

DINO^34^ is an SSL approach that leverages a knowledge-distillation framework (student–teacher network) to learn robust feature representations without the need for labeled data. In this framework, both the student and teacher networks have the same ViT architecture but with different sets of parameters. The student network is trained to match the output of the teacher network, while the teacher model is slowly updated using exponential moving averages of the student. This process allows the model to learn meaningful representations by contrasting different augmented views of the same image without requiring any negative sampling (that is, without contrasting against views from a different image with a different perturbation). For a given high-content image, 2 non-overlapping global crops (480 × 480 × 5) and 8 non-overlapping local crops (128 × 128 × 5) were sampled. The student network processes all crops, whereas the teacher network processes only the global crops. We adopt augmentation recommendations from ref. ^32^, in which we applied geometrical augmentation (that is, random flipping with a probability of 0.5) and intensity augmentation using the open-source Torchvision library. The intensity augmentation process includes random changes in brightness and contrast with a uniform factor sampled between [0.5, 1.5] and Gaussian blurring (kernel sizes [7, 11] with probabilities 0.4 and 0.9 for teacher and student views, respectively) with standard deviation sampled between [0.1, 2]. For more details on the DINO methodology, we refer to ref. ^34^.

#### Masked-based generative learning

##### MAE

MAE^41^ is an SSL approach that learns representations by reconstructing masked portions of the input image. The architecture consists of an encoder–decoder structure: for a given high-content image, a random subset of patches is masked out (75% masking ratio), the encoder processes only the visible patches, and the decoder reconstructs the original image from the encoded representations. In our approach, we use a grid search strategy to identify the top eight regions with the highest cell density based on intensity. For each high-content image, we use non-overlapping local crops of size 224 × 224 × 5, and at each epoch, randomly select 4 crops from these regions to ensure diverse and representative sampling. Otsu’s method is used for black tile detection to exclude tiles without information, thereby improving the quality of the training data. To further enhance the learning process, we incorporate both the original MAE loss (mean squared error (MSE)) and a Fourier-domain reconstruction loss^27^. The Fourier loss, computed as the MSE between the Fourier transforms of the reconstructed and original images, helps capture both spatial and frequency domain information, leading to more robust feature representations and improved generalization. We adopt geometrical augmentations including selective random cropping and flipping.

##### iTPN

iTPN^49^ leverages the hierarchical ViT (HiViT) architecture to learn feature representations by predicting masked patches of the input image. Similar to MAE, iTPN masks a portion of the input image and trains the model to reconstruct the missing patches. However, it adds the hierarchical concept by using HiViT which consists of multiple stages where each stage processes the input at different resolutions, progressively refining the feature representations through a series of transformer layers, enabling the model to capture both global and local context. The training process and augmentation techniques follow the same methodology as MAE. For more information on iTPN, we refer to ref. ^49^.

#### Hybrid-based learning

##### DINOv2

Building upon DINO, DINOv2^29^ introduces several technical advancements that accelerate and stabilize training at scale. DINOv2 uses a hybrid learning mechanism based on knowledge distillation that integrates contrastive and masked strategies through the concurrent optimization of both DINO and iBOT loss functions. The architecture consists of two major heads: the DINO head, which uses a contrastive mechanism optimized to learn image-level representations, and the iBOT head, which uses masked-based modeling optimized to learn local representations at the patch level. Multi-crop views are sampled as two non-overlapping global crops and eight non-overlapping local crops, augmented as described in the DINO section. The DINO head is optimized using cross-entropy, where the student is trained to match the teacher’s class token. In the iBOT head, patches passed to the student network are randomly masked (masking probability 0.5), while the teacher processes all patches without masking; the student is trained to reconstruct the masked patches using cross-entropy to match the teacher’s patch token. Additionally, DINOv2 optimizes a third loss function adopting the Kozachenko–Leonenko differential entropy estimator^81^ to encourage uniform feature distribution and regularize training. For more information on DINOv2, we refer to ref. ^29^.

### Experimental settings

To establish an unbiased comparison across SSL methods, we used the same base ViT architecture with a multi-channel patch size of 16 × 16 (ViT-B16) as the backbone for all models. All models were trained on a high-performance NVIDIA DGX node equipped with 8 A100 GPUs (80 GB RAM each) and 40 CPUs with 512 GB RAM. The inference pipeline used a single GPU on the same platform. Models were implemented using CUDA 11.7, TorchVision 0.15.0 and PyTorch 2.0.0. All models were trained for 400 epochs using the AdamW optimizer. Model-specific hyperparameters are detailed below.

#### MAE

The default hyperparameters for training MAE were used^41^, with a few modifications: batch size per GPU of 32 (effective batch size 256), base learning rate of 0.0015, mask ratio of 0.75, patch size of 16 × 16. The learning rate warmup period was set to 40 epochs, followed by a cosine learning rate decay schedule to gradually reduce the learning rate to a minimum value of 0 over the remaining epochs. Training used mixed precision floating point with PyTorch Distributed Data Parallel (DDP).

#### iTPN

The default hyperparameters for training iTPN were used^49^, with a few modifications: batch size per GPU of 8 (effective batch size 64), base learning rate of 0.0015, mask ratio of 0.75 and patch size of 16 × 16. The learning rate warmup period was set to 40 epochs, followed by a cosine learning rate decay schedule to gradually reduce the learning rate to a minimum value of 0 over the remaining epochs. Training used mixed precision floating point with DDP.

#### DINO

The default hyperparameters for training DINO were used^34^, with a few modifications: batch size per GPU of 64 (effective batch size 512), base learning rate of 0.00025, single precision floating point (fp32), gradient clipping of 0 and teacher network momentum of 0.9995. Training was performed using the PyTorch module of DDP.

#### DINOv2

The default hyperparameters for training DINOv2 were used^29^, with a few modifications: batch size per GPU of 64 (effective batch size 512), base learning rate of 0.001, teacher network momentum of 0.996 and teacher temperature schedule from 0.03 to 0.04 over 20,700 iterations. Both DINO and iBOT heads consisted of 65,536 prototypes, with iBOT masking probability of 0.5. Training used mixed precision floating point with Fully Sharded Data Parallel.

Moreover, we evaluated different training strategies, focusing on DINOv2, which lacks ablation studies in the context of HCI analysis unlike previously studied contrastive^32^ and masked-based models^27^. Our evaluation covered the following settings:

1. Data diversity: We compared DINOv2 models pretrained on multiple sources versus a single source, evaluating two factors: sheer volume of images and chemical diversity. This evaluation aimed to determine the impact of data diversity on the robustness of the learned representations. The single-source dataset included 591,101 data points (that is, a data point represents a 5-channel high-content image) from all unannotated perturbations in source 3 (58,662 compounds) with all 9 fields of view per well. The multi-source dataset comprised 353,607 data points from 7 sources (119,876 compounds) using one randomly sampled FOV per well.
2. Cross-batch sampling: To incorporate domain adaptation strategies into the learning process^44,45^ and assess their impact on representation robustness, we used metadata to sample multi-crop views of the same treatment across different experimental batches and sources. By default, no data augmentation was applied. In this setup, two global crops were sampled from two different batches or sources, and local crops were collected from an HCI image sampled from another batch. If any of these sampling conditions were not met, the associated crop was augmented as previously explained in the DINO section. Moreover, we also evaluated the effect of applying data augmentation systematically to all sampled crops.
3. Resolution of patch token: Besides our base patch resolution of 16 × 16 pixels across all 5 channels, we also evaluated the impact of a higher token resolution of 8 × 8 pixels. This comparison aimed to understand how different resolutions affect the granularity and quality of the learned representations.
4. Input image dimensions (token-sequence length): The base model used global and local crops at 480 × 480 and 128 × 128 pixels, respectively. We compared this with a model trained on shorter token sequences derived from global crops of 192 × 192 pixels and local crops of 64 × 64 pixels. This variation in crop size directly influences the patch token-sequence length, allowing for the evaluation of the impact of token-sequence length on model performance.
5. Architecture size: In addition to our base ViT-B architecture (86 million parameters), we experimented with a smaller architecture of ViT-S (22 million parameters) to assess the trade-off between model capacity and performance.

### SSL feature extraction

The pretrained FMs were locked during inference and used to extract SSL-based features (embeddings) to enable downstream analysis. The inference pipeline was applied on the HCI test set, ${X}_{\mathrm{tst}}=\left\{{x}_{\mathrm{tst}}^{\left(1\right)},\,{x}_{\mathrm{tst}}^{\left(2\right)},\ldots ,\,{x}_{\mathrm{tst}}^{\left({N}^{^{\prime} }\right)}\right\},$ where ${I}_{{\rm{c}}}^{(i)}\in\,{{{{\mathbb{R}}}}}^{h\times w\times 5}$ and *N*′ is the total number of test images, with each image representing an FOV. Of note, all FOVs per well were processed and the extracted features were aggregated using the arithmetic median to provide a well-level representation, ${z}_{\mathrm{well}}\in{{{{\mathbb{R}}}}}^{k}$, of *k*-dimensional features. Each HCI image was divided into (*n*) non-overlapping and non-empty image crops, such that ${x}_{\mathrm{tst}}^{\left(i\right)}=\left\{{I}_{c}^{\left(i\right)},c=1,\ldots ,{n}_{\mathrm{crops}}\right\}$, where $\langle k(T)\rangle =\frac{1}{\langle \tau (T)\rangle }=\frac{\frac{{g}_{1}}{{\tau }_{1}}+\frac{{g}_{2}}{{\tau }_{2}}\exp (-\frac{{\Delta }{E}_{12}}{{k}_{{\rm{B}}}T})}{{g}_{1}+{g}_{2}\exp (-\frac{{\Delta }{E}_{12}}{{k}_{{\rm{B}}}T})}.$ and *h* × *w* is the crop’s spatial dimensions. Each image crop was then analyzed by the pretrained FM. For optimal performance, the crop size, *h* × *w*, was set to match the same resolution of global crops used during model training (for example, 480 × 480). Nevertheless, the model can adapt to image crops of different sizes owing to the nature of the underlying ViT architecture, which uses interpolation methods to adjust the positional embeddings accordingly^35^.

### Feature normalization

The extracted features undergo further postprocessing aimed at feature normalization and mitigating batch effects. Batch effects in cell painting refer to the systematic variations that arise from differences in experimental conditions, such as staining procedures, plate layouts, processing times and equipment calibration^54^. These technical variations can introduce unwanted noise and bias into the extracted features, potentially confounding the biological signals and affecting the robustness and reproducibility of downstream analyses. Among the widely adopted normalization methods for morphological profiling are sphering^69,82^, robust MAD^1,32,33^ and more recently, Harmony^70^, which has shown promising results when combined with these methods^9,54^. We performed an ablation analysis to assess the impact of each normalization method, individually and in combination, on the learned representations from the various SSL models. These methods were implemented using the public Python packages Pycytominer^83^ (v1.0.1) and HarmonyPy (v0.0.9).

#### Sphering

Sphering normalizes features across experimental batches by optimizing a transformation matrix that maps features into a space where technical variations are minimized. We adopted the method as previously described^69^ and applied it to well-level aggregated features. A whitening transform (PCA without dimensionality reduction) was computed on DMSO embeddings from different experimental batches: embeddings were centered by subtracting the feature mean, the covariance matrix was decomposed into eigenvalues and eigenvectors, and the whitening transform was derived to produce zero-mean, unit-variance, decorrelated features. The regularization parameter was set to 1 unless otherwise stated. The optimized transform was subsequently applied to all embeddings (treatment and control), standardizing features across experimental conditions.

#### Robust MAD

The robust MAD normalization algorithm was applied on a per-plate basis, as shown in equation (1). For a given plate (*p*), the median and median absolution deviation were calculated based on the plate-wise embedding statistics of all the held-out wells. The plate embeddings (*Z*^(*p*)^), which are a set of well-level aggregated embeddings, were then transformed accordingly to yield a set of robust MAD normalized features (${Z}_{\mathrm{MAD}}^{\left(p\right)}).$

1 $${Z}_{\mathrm{MAD}}^{\left(p\right)}=\frac{{Z}^{\left(p\right)}-\mathrm{{median}}\left({Z}^{\left(p\right)}\right)}{\mathrm{MAD}\left({Z}^{\left(p\right)}\right)},$$

where

$$\,\mathrm{MAD}\left({Z}^{(p)}\right)=\mathrm{median}(|{z}_{i}-\,\mathrm{median}({Z}^{(p)})|),\forall \,{z}_{i}\in {Z}^{(p)}$$

#### Harmony

Harmony is an iterative batch-correction algorithm originally developed for single-cell omics^70^ and recently adopted in morphological applications^9,54^. It uses PCA and soft clustering iteratively to align embeddings from different batches. The integration variable was specified as the experimental batch for within-source alignment (that is, data from the same laboratory). Harmony assigns data points (well-level representations) to multiple clusters, iterating to favor clusters represented by different batches while penalizing clusters overrepresented by a single batch. Cluster-specific linear correction factors are computed based on centroids, where a cluster optimally represents a specific phenotype. These correction factors define a linear adjustment that maps data points into a new space sensitive to decouple intrinsic morphological phenotypes from technical variations.

### Baseline representation (CellProfiler)

The raw CellProfiler features are available as part of the JUMP–CP dataset. We followed a standard preprocessing pipeline^12,54^ and implemented it using the Python package Pycytominer^83^. The preprocessing pipeline consists of several steps: excluding low-variance features, plate-wise robust MAD normalization using negative control (DMSO) and feature selection. We applied several standard feature selection operations, implemented in Pycytominer, to remove missing data (‘drop_na_columns’), remove outliers (‘drop_outliers’), remove low-variance features (‘variance_threshold’), remove highly correlated features (‘correlation_threshold’) and remove a list of previously observed unstable and noisy features (‘blocklist’)^12,84^.

### Embedding visualization

The extracted high-dimensional features were visualized in a 2D map using the non-linear dimensionality reduction algorithm *t*-SNE. The default settings of *t*-SNE^85^, as implemented in the Python package ‘Scikit-learn’^86^ (v1.0.2), were used for this visualization unless otherwise stated.

### Compound retrieval tasks

We implemented standardized compound retrieval tasks^1,32,64^ divided into two assessments: reproducibility and biological relevance. The reproducibility assessment, termed phenotypic distinctiveness, evaluates the ability to retrieve replicates of the same compound across different experimental batches. We used two selection criteria: PNSB and PNSBW. PNSBW acts as a more stringent criterion controlling for both experimental-batch and well-position confounding effects, whereas PNSB is susceptible to well-position effects. Therefore, careful interpretation of phenotypic distinctiveness (PNSB) results is necessary. Owing to dataset limitations, the current JUMP–Target2–compounds dataset provides fewer data points meeting the PNSBW criteria for source-wise analysis—specifically, 14 positive controls (‘poscon_diverse’) offered by every source. By contrast, the RxRx1 dataset offers more data points that meet the PNSBW criteria. Consequently, the phenotypic distinctiveness assessment is conducted using either PNSB or PNSBW, depending on data availability. The biological relevance task, termed phenotypic consistency, uses the selection criteria of NSCB. Phenotypic consistency (NSCB) assesses the representation’s ability to retrieve a different compound from different experimental batches, where the queried representation shares the same gene target as the retrieved compound. All PNSB-, PNSBW- and NSCB-based tasks used batch-level median aggregation of compound representations. Cosine similarity was used to measure embedding similarities, and the mAP was used as the performance metric. Notably, the absolute value of the cosine similarity was considered for the NSCB task because compounds annotated for the same gene target might be positively or negatively correlated, as explained in ref. ^1^.

### Mechanism of action classification task—BBBC021

Generalizability on different cell types was assessed using few-shot learning through *k*-NN classification (default *k* = 1), following previous studies^82,87^. This task classifies 12 MoAs associated with 103 treatments (compound–concentration pairs). Our FMs were applied for feature extraction after adding two zero-padded channels to the original three channels. Extracted features were mean-aggregated at the treatment level and normalized with respect to DMSO embeddings using a sphering transform. Cosine distance measured similarities between aggregated embeddings. Evaluation used a leave-one-treatment-out approach with two accuracy metrics: NSC, which restricts matches to exclude the same compound, and NSCB, which additionally excludes matches from the same batch. The default assessment for both metrics was based on top-1 accuracy, with qualitative assessment conducted using *t*-SNE visualization.

### Evaluation metrics for downstream tasks

The model performance on downstream tasks was quantitatively assessed using cosine similarity and mAP.

#### Cosine similarity

Cosine similarity was used as metric to measure the directional similarities between embeddings (*Z*). The cosine similarity between a query embedding (*z*_*q*_) and the *i*th embedding (*z*_*i*_) is given by equation (2). The range of cosine similarity is between [−1, 1], where values of 1 and −1 indicate that the two embeddings have identical and opposite directions, respectively. A cosine similarity of 0 means the two embeddings are orthogonal. Cosine similarity was used for JUMP–Target2–compounds retrieval tasks, whereas cosine distance was used for the BBBC021 classification task, as shown in equation (3). The sampling for embedding matches was based on the following task-specific criteria:

- NSC task: *z*_*q*_ ∈ *Z*_*c*_and *z*_*i*_ ∈ *Z*\*Z*_*c*_, where *Z*_*c*_ holds all embeddings that belong to compound *c*. The term (*Z*\*Z*_*c*_) means embeddings in *Z*_*c*_ were excluded from *Z*. NSC evaluates the retrieval of an embedding *z*_*i*_ from different compound *c*, relative to a query embedding *z*_*q*_.
- PNSB task: *z*_*q*_ ∈ *Z*_*b*_ and *z*_*i*_ ∈ *Z*\*Z*_*b*_, where *Z*_*b*_ holds all embeddings that belong to experimental batch *b*. PNSB evaluates the retrieval of an embedding *z*_*i*_ from different experimental batch *b*, relative to a query embedding *z*_*q*_. While the PNSB metric primarily evaluates the retrieval of embeddings across different experimental batches, it does not consider well position as a confounding factor; therefore, interpretation should account for this limitation.
- PNSBW: *z*_*q*_ ∈ *Z*_[*b*,*w*]_ and *z*_*i*_ ∈ *Z*_[*b*, *w*]_, where *Z*_[*b*, *w*]_ holds all embeddings that belong to experimental batch *b* and embeddings from well position *w* across the entire dataset. For a given query embedding *z*_*q*_ representing compound *c* in batch *b* and acquired at well *w*, the PNSBW metric applies two exclusion criteria before sampling *z*_*i*_: (1) exclude all embeddings of compound *c* acquired at well position *w* across all batches, and (2) exclude all embeddings from batch *b*, regardless of compound. PNSBW evaluates the retrieval of an embedding *z*_*i*_ from different *b* and different *w*, relative to the query *z*_*q*_.
- NSCB task: *z*_*q*_ ∈ *Z*_[*c*, *b*]_and *z*_*i*_ ∈ *Z*\*Z*_[*c*, *b*]_, where *Z*_[*c*, *b*]_holds all embeddings that belong to compound *c* across the entire dataset and all embeddings in experimental batch *b*. For a given query embedding *z*_*q*_ representing compound *c* at batch *b*, the NSCB metric applies two exclusion criteria before sampling *z*_*i*_: (1) exclude all embeddings of compound *c* across all batches, and (2) exclude all embeddings from batch *b*, regardless of compound. The evaluation measures the retrieval of an embedding *z*_*i*_ from a different compound *c* and different experimental batch *b*, relative to the query *z*_*q*_.

2 $${\mathrm{Cosine}}_{\mathrm{Sim}}\left({z}_{q},{z}_{i}\right)=\frac{{z}_{q}.{z}_{i}}{\parallel {z}_{q}\parallel \parallel {z}_{i}\parallel }$$

3 $${\mathrm{Cosine}}_{\mathrm{distance}}\left({z}_{q},{z}_{i}\right)=1-{\mathrm{Cosine}}_{\mathrm{Sim}}{\left({z}_{q},{z}_{i}\right)}_{q}$$

#### mAP

Average precision (AP) was used to evaluate the ranking performance based on cosine similarity. AP is computed by considering the rank of relevant embeddings in the sorted list of embeddings based on cosine similarity scores relevant to the query embedding. The AP is calculated as the average of the precision values at each rank (*k*) where a relevant embedding is found. An AP of 1 indicates perfect retrieval, whereas an AP of 0 indicates irrelevant retrievals. The formula for AP is given by:

4 $$\mathrm{AP}=\,\sum _{k}\left({R}_{k}-{R}_{k-1}\right){P}_{k}$$

where *R*_*k*_ and *P*_*k*_ are the recall and precision at the *k*th rank (that is, threshold).

As AP was calculated for replicates of each perturbation, the mAP for a compound (*c*) is calculated by averaging the AP values of all its replicates (*M*) (equation (5)). This provides a robust measure of the model’s performance across multiple instances of the same perturbation, ensuring that the evaluation is not biased by any single replicate.

5 $$\mathrm{{mAP}}(c)=\frac{1}{M}\mathop{\sum }\limits_{i=1}^{M}A{P}_{i}\left(c\right)$$

We adopted the statistical framework developed in ref. ^64^, implemented in the open-source Python package copairs, which standardizes metric reporting for retrieval tasks in morphological profiling. This framework uses mAP, as defined in equations (4) and (5), together with statistical significance testing to evaluate three retrieval tasks: phenotypic activity (a perturbation’s ability to retrieve its own replicates against negative controls); phenotypic consistency (retrieval of perturbations sharing a common biological annotation, such as MoA, reported following NSC and NSCB criteria); and phenotypic distinctiveness (retrieval of replicates against other perturbations, reported following PNSB and PNSBW criteria). The copairs framework estimates a *P*value for each average precision score using a permutation-based significance test against a null distribution. In our setup, this null distribution is generated by shuffling the rank list 100,000 times, and the observed score is evaluated against it. *P* values are adjusted for multiple testing using the Benjamini–Hochberg method. Performance is reported as the percentage of retrieved classes with corrected *P* values below the significance threshold of 0.05, along with the overall mAP at the task level. For more information, we refer to ref. ^64^.

### Statistical significance

To assess the statistical significance of retrieval task performance between CellProfiler and each SSL method, two-sided *t*-tests were conducted using the ‘ttest_ind’ function from the SciPy library. Exact *P* values from these tests are reported in the ‘Results’ section.

### Quantitative metrics for assessing batch effect correction

For large-scale evaluation, the impact of batch effect correction was assessed using the performance of three retrieval tasks: phenotypic activity, phenotypic consistency and phenotypic distinctiveness. For smaller-scale evaluations (for example, positive control of Target2–compounds), we used two complementary methods: silhouette and *k*-NN graph connectivity. The silhouette value assesses how well an object is clustered with its own group compared with others. High values indicate good clustering, while low or negative values suggest potential issues with the number of clusters. The *k*-NN graph connectivity method is based on *k*-NN graph algorithm to measure how well samples of the same biological annotation are connected, assuming that effective batch effect removal results in close proximity of these elements. Together, these methods provide a quantitative evaluation of batch effect correction efficiency and how well the methods ensure the integrity of the biological information after correction^54,88,89^.

### Spatial visualization of local representations (patch tokens)

To characterize the learned representations of DINO and DINOv2, we developed a pipeline to quantify how well local representations (that is, patch token) capture nuclear morphology (Extended Data Fig. 3b). High-content images were processed, using the pretrained DINO and DINOv2 models, to extract patch tokens. PCA was applied in two stages^29^: first on the entire set of patch tokens, where the first principal component was used to generate a foreground mask separating cellular content from background; then exclusively on the foreground patch tokens, retaining the first three principal components as features. The resulting three-dimensional PCA features were subjected to *K*-means clustering (*K* = 5) to identify distinct cellular sub-structures within the foreground. To identify the nucleus cluster, a ground-truth binary mask was obtained by applying Otsu’s thresholding to the DNA channel. The Pearson correlation coefficient was calculated between this ground-truth mask and each *K*-means cluster mask; the cluster with the highest correlation was designated as the nucleus cluster. The relative area of the nucleus identified from the SSL-derived cluster was then correlated with the original nucleus area from the DNA channel to quantify the performance of the local representations in capturing nuclear size. This analysis was conducted across 3 positive-control compounds—SB-203580, NVS-PAK-1 and NVP-AEW541—totaling 2,980 high-content images from all available sources and batches.

### Reporting summary

Further information on research design is available in the Nature Portfolio Reporting Summary linked to this article.

## Online content

Any methods, additional references, Nature Portfolio reporting summaries, source data, extended data, supplementary information, acknowledgements, peer review information; details of author contributions and competing interests; and statements of data and code availability are available at https://doi.org/10.1038/s41592-026-03270-z.

## Supplementary information

Supplementary Information Supplementary Figs. 1–17, Supplementary Tables 1–13 and Supplementary Notes 1–4.

Reporting Summary

## Figures and Tables

**Extended Data Table 1:**
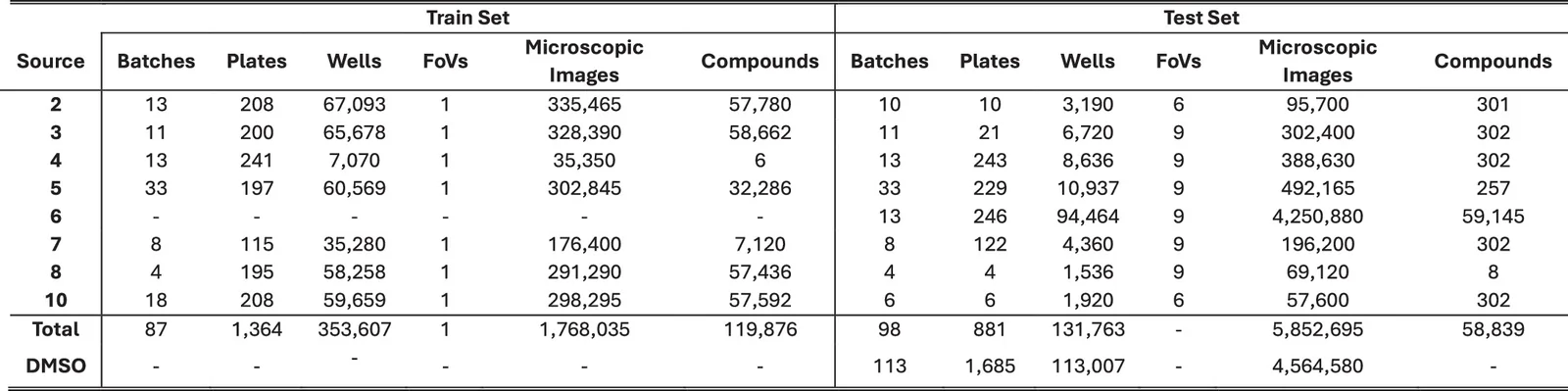
Summary statistics of JUMP-CP data (cpg0016) distribution within each of the training and test sets

Summary statistics of JUMP-CP data (cpg0016) distribution within each of the training and test sets. The table details the number of experimental batches, plates, wells, images, and compounds. The training set holds all unannotated perturbations from multi-sources, and a random field-of-view (FoV) was sampled from each well. The test set consists of Target2-Compounds from all sources, along with Source6 data that was held out entirely for evaluation purposes.

**Extended Data Table 2:**
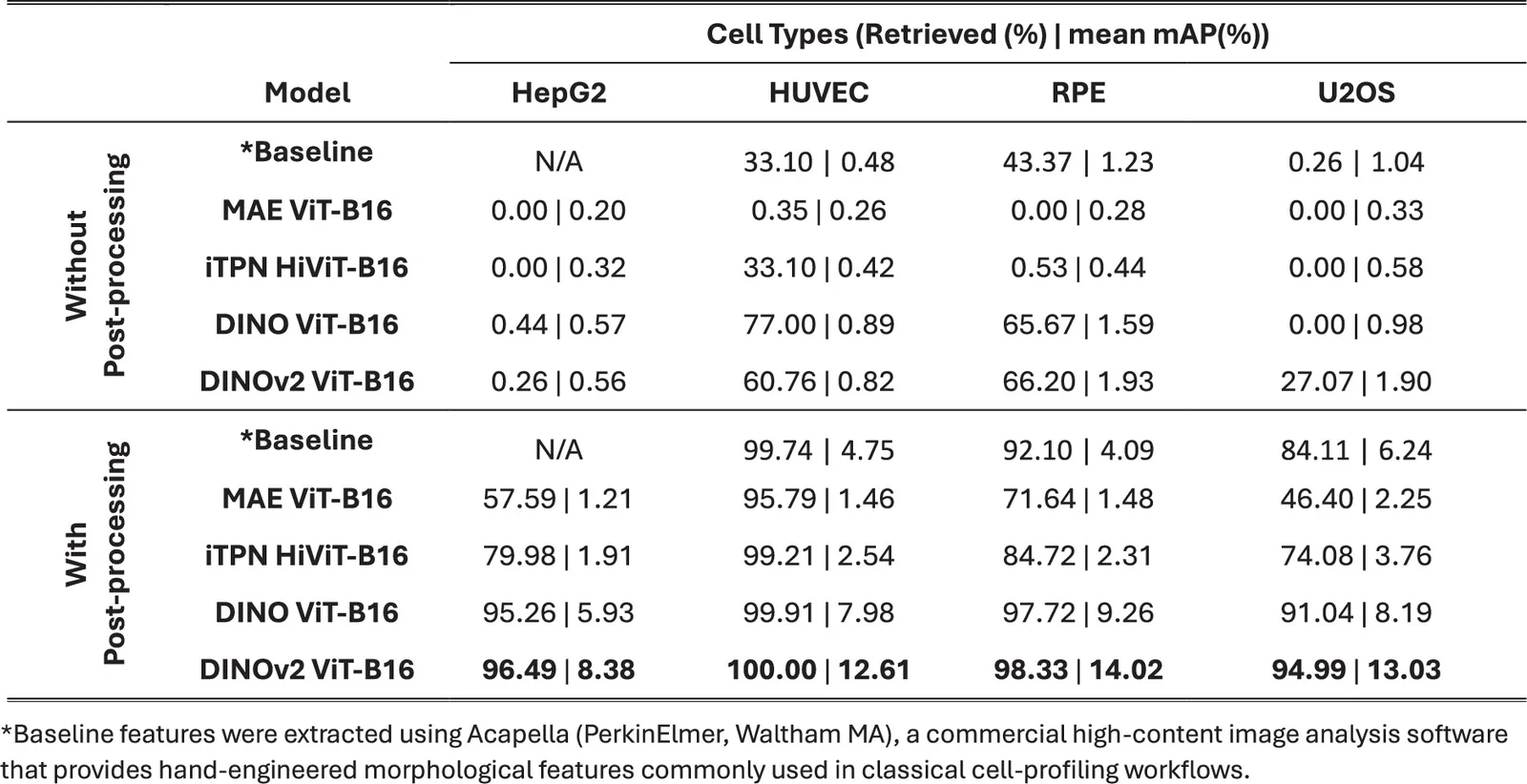
Evaluation of the Phenotypic Distinctiveness (PNSBW) task on RxRx1 Dataset (1,138 genetic perturbations in 51 experimental batches across 4 cell types), comparing performance across different models

Evaluation of the Phenotypic Distinctiveness (PNSBW) task on RxRx1 Dataset (1,138 genetic perturbations in 51 experimental batches across 4 cell types), comparing performance across different models. Postprocessing consistently yields substantial improvements for both the percentage of retrieved classes and mean mAP, which reflects improved batch effect correction and phenotypic discrimination.

## Data Availability

All the datasets used in this study are publicly available. The principal JUMP–CP dataset of cpg0016 is available from the Cell Painting Gallery on the Registry of Open Data on AWS (https://registry.opendata.aws/cellpainting-gallery/)^80^. The BBBC021 dataset is available from the Broad Bioimage Benchmark Collection (https://bbbc.broadinstitute.org/BBBC021)^50,51^. The RxRx1 is available by ref. ^52^ and it can be downloaded at https://www.rxrx.ai/rxrx1. The EU-OPENSCREEN Bioactives dataset (cpg0036) is available by ref. ^53^ and can be downloaded from the Cell Painting Gallery (accession cpg0036-EU-OS-bioactives). The embeddings derived from our models are deposited via Zenodo at https://doi.org/10.5281/zenodo.21036400(ref. ^90^). The pretrained FMs (CC BY-NC-SA 4.0) are publicly available at https://github.com/johnsonandjohnson/DeepCellMorpho.

## Code availability

The pretrained FMs (CC BY-NC-SA 4.0), inference code and figure generation code are publicly available at https://github.com/johnsonandjohnson/DeepCellMorpho.
