# Supplementary material for: Advancing morphological profiling with self-supervised learning through comparative insights into representation learning of cellular perturbations
Source: Nat Methods. 2026 Oct 29;23(11):2181–94. doi: 10.1038/s41592-026-03270-z (PMC13736651; doi:10.1038/s41592-026-03270-z)
Supplement: Supplementary file 1 [file 41592_2026_3270_MOESM1_ESM.pdf]

nature methods

Supplementary information https://doi.org/10.1038/s41592-026-03270-z

Advancing morphological profiling with self-supervised learning through comparative insights into representation learning of cellular perturbations

In the format provided by the authors and unedited

Supplementary Material

Table of contents:

1. Supplementary Notes
2. Supplementary Tables
3. Supplementary Figures

1. Supplementary Notes

Supplementary Note 1: Feature Engineering Limitations of Classical Approaches

Despite applying the same standard Pycytominer pipeline for CellProfiler feature selection across all sources[1,2]it yielded suboptimal performance on Source8 (Supplementary Fig. S4). The t-SNE visualization of the selected CellProfiler features reveals a noisy distribution of data points from various experimental batches, forming an outer circular shape (Supplementary Fig. S4.a). This pattern reflects the need for further filtration of the selected features or the adoption of a different feature selection framework, such as those employed in genomics[3,4]. This noisy distribution impacted the quantitative evaluation of the reproducibility task (PNSB retrieval), explaining the underperformance of CellProfiler compared to SSL (Supplementary Fig. S4.b). In contrast, SSL-based methods consistently demonstrated robustness on Source8 without any further feature selection or filtration.

Supplementary Note 2: Token Resolution, Model Capacity, and Backbone Architecture

Token resolution and sequence length: The DINOv2 model trained with higher token resolution (ViT-B8) underperformed, likely due to the quadratic complexity of the self-attention mechanism. Specifically, models were pretrained on global crops of 480×480 pixels, resulting in an increase in token sequence length from 900 for ViT-B16 to 3,600 for ViT-B8. At this longer sequence length, the model's capacity (86 million parameters) was limited by the increased data complexity, leading to optimization challenges (Supplementary Fig. S10). The DINOv2 ViT-B8 model pretrained on shorter token sequence outperformed its counterpart pretrained on a longer sequence (Table 1, Supplementary Tables S3-S4). Nevertheless, at lower sequence resolutions, there was no significant difference between ViT-B16 pretrained on shorter or longer sequence lengths for input image sizes of 192×192 and 480×480 pixels (Supplementary Fig. S9), which resulted in sequence lengths of 144 and 900 – both manageable given the model's capacity. While finer patch sizes can provide more detailed spatial features, their benefits depend on maintaining manageable sequence lengths that do not overwhelm model capacity. These findings align with recent observations in scaling laws for vision transformers[5,6], underscoring the importance of balancing patch size, input resolution, and model capacity to optimize ViT performance.

Backbone architecture comparison: To understand how backbone architecture influences performance, we evaluated the phenotypic distinctiveness task (PNSB) on JUMP-Target2-Compounds using two models pretrained on the same JUMP-CP training dataset (119,876 compounds): one with a Swin Transformer V2 Base backbone (window size 16) [7,8] and another with ViT-B16. ViT divides images into patch tokens[9] and applies a standard transformer[10], modeling long-range dependencies effectively. SwinV2 employs a hierarchical design with shifted windows to capture local and global features across multiple scales[8]. Without postprocessing, ViT-B16 slightly outperformed SwinV2-B-W16 in both retrieval rate (19.93% vs. 18.63%) and retrieval strength of mean mAP (7.34% vs. 6.36%). After postprocessing, ViT-B16 achieved 100% retrieval and 42.27% mean mAP, while SwinV2-B-W16 reached 94.12% retrieval and 29.45% mean mAP (Supplementary Table S11). These findings suggest that ViT's capacity for modeling long-range dependencies provides more robust representations for phenotypic discrimination[11,12]. Source-wise results are detailed in Supplementary Fig S8. A comprehensive comparison of backbone architectures remains a direction for future research.

Supplementary Note 3: Reconstruction Quality of Masked-Based Models

The learning strategies of masked-based models and contrastive-based models are fundamentally different, leading to distinct approaches for interpreting their visual capabilities. Masked-based models, such as MAE and iTPN, operate by masking parts of the input and reconstructing the missing portions, thereby capturing context and structural information. In contrast, contrastive-based models, such as DINO, utilize a knowledge-distillation framework where a student-teacher network architecture is optimized to learn meaningful representations by contrasting different augmented views of the same image without the need for negative sampling. To interpret the capabilities of these models in capturing informative visual features, we analyzed the local patch representations for models like DINO and DINOv2, as shown in see ED Fig 3 and Supplementary Figs. S11 and S12, revealing cellular semantic structures. For masked-based models, the quality of data reconstruction serves as the key indicator of their effectiveness. A visual comparison of image reconstruction quality across masked-based models – iTPN HiViT-B16, iTPN HiViT-B8, MAE ViT-B16, and MAE ViT-B8 – is shown in Supplementary Fig. S13. We incorporated a recently introduced Fourier-domain reconstruction loss into the iTPN HiViT-B8 model. This loss function, which has been successfully integrated with MAE, has demonstrated potential for improved data reconstruction and model convergence[12]. The Fourier loss operates in the frequency space, encouraging the model to better reconstruct textures and reduce background artifacts. This enhancement is visually evident in the improved reconstructed images, which show fewer artifacts and more accurate textures of cellular morphology. The Fourier loss also facilitates more reliable navigation of the loss landscape during reconstruction, leading to overall better performance[12].

Supplementary Note 4: Explainability and Interpretability in SSL for HCI

Traditional machine learning methods, such as those used by CellProfiler, offer greater potential for feature interpretability[13]. However, with the rise of advanced neural-networks based modeling, performance has significantly increased, leading the scientific community towards deep learning techniques. Despite their superior performance, deep learning methods often lack interpretability, working as "black boxes." To address this, Explainable AI (XAI) techniques are essential, particularly in drug discovery, where understanding model decisions is crucial for ensuring reliability and safety of potential treatments[14]. Recent works, such as Grad-CAMO, aim to measure model attention on relevant biological features versus irrelevant background noise[15]. Similarly, CytoSummaryNet uses SSL within a multiple-instance learning (MIL) framework to enhance interpretability and performance of single-cell morphological profiles[16]. Despite these advancements, interpretability remains a significant challenge[14]. Future directions involve developing more sophisticated XAI methods tailored to specific applications. For example, in SSL-based approaches, XAI methods could help clarify how models learn from unlabeled data, ensuring learned representations are relevant for downstream tasks. Currently ongoing research highlights the need for continuous innovation to achieve truly interpretable and trustworthy AI systems in complex biological challenge[14] .

2. Supplementary Tables

Table S1 – Comparative evaluation of the biological relevance task (NSCB) on Target2-Compounds. A source-wise analysis of gene target retrieval (mAP ≥ 0.25) across various methods.

SSL Model	source	Common Targets	Exclusive Cell Profiler	Exclusive SSL Model
**MAE ViT-B16**	2	HSP90AB1, CDK2	TUBB4B, CDK9, PTPN2, S1PR1	PAK1
	3	TUBB4B, PTPN2, CDK2	MAPK14, HSP90AB1	PAK1
	4	---	CDK9, MAPK14, HSP90AB1, CDK2	---
	5	TUBB4B, HSP90AB1	---	---
	6	S1PR1	PTPN2, ANXA1, CDK2, TUBB4B, HSP90AB1	---
	7	---	CDK9, P3H1, FGF1, PRKCB, LPAR1, GLRA3	TUBB4B, PTPN2, S1PR1, PAK1
	10	HSP90AB1	TUBB4B, RPL3, CDK2	---
**iTPN HiViT-B16**	2	HSP90AB1, CDK2	TUBB4B, CDK9, PTPN2, S1PR1	PAK1
	3	PTPN2, HSP90AB1, CDK2	TUBB4B, MAPK14	PAK1
	4	CDK2	CDK9, MAPK14, HSP90AB1	---
	5	TUBB4B, HSP90AB1	---	---
	6	PTPN2, ANXA1, CDK2, TUBB4B, HSP90AB1, S1PR1	---	---
	7	---	CDK9, P3H1, FGF1, PRKCB, LPAR1, GLRA3	PORCN, PTPN2, OPRL1, TUBB4B, RPL23A, S1PR1, MAPK14, PAK1
	10	HSP90AB1, CDK2	TUBB4B, RPL3	DDR2
**DINO ViT-B16**	2	PTPN2, HSP90AB1, S1PR1, CDK2	TUBB4B, CDK9	MAPK14, KCNMA1, ANXA1
	3	MAPK14, PTPN2, HSP90AB1, CDK2	TUBB4B	CDK9, S1PR1, PAK1
	4	MAPK14, HSP90AB1, CDK2	CDK9	PAK1
	5	HSP90AB1	TUBB4B	---
	6	PTPN2, ANXA1, CDK2, TUBB4B, HSP90AB1, S1PR1	---	CDK9
	7	CDK9	P3H1, FGF1, PRKCB, LPAR1, GLRA3	TUBB4B, TUBB3, PTPN2, S1PR1
	10	HSP90AB1, CDK2	TUBB4B, RPL3	PTPN2
**DINOv2 ViT-B16**	2	CDK9, PTPN2, CDK2, HSP90AB1, S1PR1	TUBB4B	MAPK14
	3	MAPK14, PTPN2, HSP90AB1, CDK2	TUBB4B	CDK9, S1PR1, PAK1
	4	CDK9, MAPK14, HSP90AB1, CDK2	---	---
	5	TUBB4B, HSP90AB1	---	CDK9
	6	PTPN2, ANXA1, CDK2, TUBB4B, HSP90AB1, S1PR1	---	CDK9, MAPK14, JAK1
	7	CDK9	P3H1, FGF1, PRKCB, LPAR1, GLRA3	PTPN2, MMP2, TUBB4B, LCK, S1PR1, TUBB3, MAPK14, PAK1
	10	HSP90AB1, CDK2	TUBB4B, RPL3	CDK9, PTPN2

Table S2 – Quantitative evaluation of batch effect correction across different models on a smaller subset of JUMP-Target2-Compounds. Postprocessing consistently results in significant improvements in both the Silhouette and Graph Connectivity scores.

	Model	Targets of Interest*		Positive Control (cp, orf, dv)	
		Silhouette Score ↑ better	k-NN Graph Connectivity ↑ better	Silhouette Score ↑ better	k-NN Graph Connectivity ↑ better
Without Post-processing	**CellProfiler**	-0.1201	0.2983	-0.2789	0.1396
	**MAE ViT-B16**	0.0003	0.4319	-0.1469	0.3401
	**iTPN HiViT-B16**	0.2253	0.6305	0.0221	0.5708
	**DINO ViT-B16**	0.1153	0.5367	-0.0773	0.4737
	**DINOv2 ViT-B16**	0.1594	0.5402	-0.0738	0.4779
With Post-processing	**CellProfiler**	0.5197	0.9263	0.1984	0.7781
	**MAE ViT-B16**	0.0232	0.5476	-0.0980	0.5030
	**iTPN HiViT-B16**	0.4472	0.9674	0.1535	0.7645
	**DINO ViT-B16**	0.7204	0.9901	0.3130	0.8589
	**DINOv2 ViT-B16**	**0.8139**	**0.9926**	**0.3355**	**0.8806**

*Targets of interest are highlighted in Figure 2.d.

Table S3 – Evaluation of the Phenotypic Distinctiveness (PNSB): Source-wise Analysis of the JUMP-Target2-Compounds (~302) across different models. Postprocessing consistently yields significant performance improvements, as indicated by both the mean mAP and the percentage of classes retrieved with corrected p-value below significance threshold of 0.05.

		**Without Post-processing**							**With Post-processing**						
	**Model / Source**	**2**	**3**	**4**	**5**	**6**	**7**	**10**	**2**	**3**	**4**	**5**	**6**	**7**	**10**
Retrieved (%)	**CellProfiler**	32.89	22.22	21.57	17.97	22.88	1.85	0.98	86.23	87.91	99.67	99.22	99.67	4.94	84.64
	**MAE ViT-B8**	29.18	2.29	36.27	27.73	34.31	6.75	24.18	90.82	85.62	97.06	98.05	96.73	35.80	82.68
	**MAE ViT-B16**	28.20	13.40	31.37	26.56	41.50	13.50	18.30	89.84	81.70	98.69	99.22	100.00	33.33	80.07
	**iTPN HiViT-B8**	32.46	2.94	38.56	28.52	37.58	5.52	29.08	96.07	94.44	100.00	100.00	100.00	40.12	96.73
	**iTPN HiViT-B16**	34.75	3.27	38.24	30.08	42.16	9.82	26.80	97.38	94.12	100.00	99.61	99.67	42.59	98.69
	**DINO ViT-B16**	24.59	1.63	24.18	15.23	30.07	0.61	19.93	100.00	98.69	100.00	100.00	100.00	45.68	100.00
	**DINOv2 ViT-B16**	32.13	2.29	21.57	20.31	32.03	4.91	20.26	98.36	78.43	100.00	100.00	100.00	39.51	99.67
	**DINOv2 ViT-B16 cross-batch (no aug)**	30.49	24.84	41.50	22.66	34.31	30.06	22.88	85.25	71.90	99.67	98.44	100.00	41.98	78.10
	**DINOv2 ViT-B16 cross-batch**	36.39	28.43	51.31	23.83	38.89	29.45	26.80	97.70	88.56	100.00	100.00	100.00	41.36	84.97
	**DINOv2 ViT-B16 one-source**	29.84	20.92	29.41	23.44	30.39	4.91	24.51	99.34	88.56	100.00	100.00	100.00	43.21	95.75
	**DINOv2 ViT-B16 (short sequence)**	29.18	2.29	24.84	20.31	31.37	6.13	21.90	100.00	97.39	100.00	100.00	100.00	38.89	99.35
	**DINOv2 ViT-B8**	25.25	1.31	14.38	14.45	31.37	0.61	15.36	72.46	59.80	100.00	98.05	100.00	31.48	48.04
	**DINOv2 ViT-B8 (short sequence)**	31.48	15.36	26.80	21.09	32.68	9.82	22.88	100.00	95.10	100.00	100.00	100.00	43.21	99.02
	**DINOv2 ViT-S16**	31.80	1.96	23.20	19.92	31.37	4.91	20.26	86.89	55.88	99.02	96.88	100.00	35.80	99.02
Mean mAP (%)	**CellProfiler**	6.23	3.27	2.40	2.90	2.85	6.40	2.10	29.66	25.67	27.49	27.25	36.63	6.09	20.73
	**MAE ViT-B8**	4.97	1.49	5.11	6.50	9.32	7.22	5.63	20.62	13.70	14.79	17.94	22.03	23.16	16.79
	**MAE ViT-B16**	6.16	2.05	4.92	6.28	10.46	9.87	4.72	20.41	16.06	14.91	17.59	21.12	23.58	13.59
	**iTPN HiViT-B8**	7.03	1.55	6.35	10.34	13.99	6.94	6.55	31.36	24.14	25.57	28.00	33.77	28.41	27.50
	**iTPN HiViT-B16**	9.95	1.76	7.04	12.90	19.79	7.66	7.36	33.99	26.99	27.37	29.14	36.88	29.92	31.40
	**DINO ViT-B16**	6.75	1.22	5.63	4.82	14.39	4.07	7.34	46.93	39.21	37.79	34.99	47.37	33.90	42.27
	**DINOv2 ViT-B16**	12.97	2.07	4.41	7.87	16.34	5.59	8.38	36.30	28.73	29.63	26.34	48.24	26.61	37.76
	**DINOv2 ViT-B16 cross-batch (no aug)**	15.50	7.54	12.99	9.54	18.46	16.14	9.69	35.94	31.81	29.71	27.15	43.74	31.54	30.59
	**DINOv2 ViT-B16 cross-batch**	20.70	10.23	16.20	11.66	21.93	17.92	12.69	43.27	37.90	34.86	29.79	50.58	33.68	34.70
	**DINOv2 ViT-B16 one-source**	10.01	3.72	6.86	7.95	11.49	5.64	8.46	38.83	27.51	30.04	31.11	41.56	28.92	29.67
	**DINOv2 ViT-B16 (short sequence)**	10.70	2.12	5.60	7.00	14.49	6.72	9.37	41.28	33.31	33.52	29.80	47.10	30.49	34.72
	**DINOv2 ViT-B8**	9.39	1.22	3.09	5.64	13.38	4.46	6.51	28.70	23.23	25.86	22.55	45.72	19.15	22.76
	**DINOv2 ViT-B8 (short sequence)**	14.93	2.81	6.84	7.68	14.80	8.08	10.49	42.23	32.50	33.24	30.37	48.24	31.72	35.23
	**DINOv2 ViT-S16**	10.52	1.88	4.88	6.70	14.97	5.50	8.59	33.96	25.86	26.50	23.66	38.37	26.60	32.46

Table S4 – Evaluation of the Phenotypic Consistency (NSCB): Source-wise Analysis of the JUMP-Target2-Compounds (~302) across different models. Postprocessing consistently yields significant performance improvements, as indicated by both the mean mAP and the percentage of classes retrieved with corrected p-value below significance threshold of 0.05.

		Without Post-processing							With Post-processing						
	**Model / Source**	**2**	**3**	**4**	**5**	**6**	**7**	**10**	**2**	**3**	**4**	**5**	**6**	**7**	**10**
**Retrieved (%)**	**CellProfiler**	0.00	0.00	0.00	3.17	0.00	0.00	0.00	8.28	4.11	8.22	14.29	10.27	0.00	2.05
	**MAE ViT-B8**	0.69	0.00	0.00	1.59	1.37	0.00	0.00	2.76	2.05	1.37	3.97	2.74	0.00	0.00
	**MAE ViT-B16**	0.00	0.00	0.68	2.38	1.37	0.00	0.00	4.14	3.42	2.05	4.76	4.11	0.94	0.00
	**iTPN HiViT-B8**	0.69	0.00	0.00	2.38	2.74	0.00	0.00	3.45	4.11	2.74	11.90	7.53	0.00	0.68
	**iTPN HiViT-B16**	0.69	0.00	0.00	2.38	2.74	0.00	0.00	3.45	4.79	3.42	11.11	7.53	0.94	0.68
	**DINO ViT-B16**	1.38	0.00	1.37	3.17	3.42	0.00	0.68	13.10	7.53	8.90	11.90	11.64	0.94	4.79
	**DINOv2 ViT-B16**	2.76	0.00	1.37	4.76	3.42	0.00	0.00	8.97	7.53	7.53	9.52	11.64	0.00	4.11
	**DINOv2 ViT-B16 cross-batch (no aug)**	3.45	2.74	1.37	4.76	4.11	0.00	0.68	8.97	7.53	3.42	9.52	10.96	0.94	2.74
	**DINOv2 ViT-B16 cross-batch**	3.45	2.74	2.05	4.76	4.11	0.00	0.68	9.66	9.59	7.53	11.11	13.70	0.94	4.11
	**DINOv2 ViT-B16 one-source**	0.69	0.00	1.37	2.38	2.74	0.00	0.00	6.90	7.53	4.79	12.70	9.59	0.00	2.05
	**DINOv2 ViT-B16 (short sequence)**	2.76	0.00	1.37	3.97	3.42	0.00	0.68	8.28	6.85	3.42	10.32	12.33	0.00	3.42
	**DINOv2 ViT-B8**	1.38	0.00	1.37	4.76	2.74	0.00	0.68	3.45	7.53	3.42	7.94	10.96	0.00	2.05
	**DINOv2 ViT-B8 (short sequence)**	2.76	0.00	1.37	3.97	4.11	0.00	0.68	8.97	8.22	8.22	10.32	11.64	4.72	3.42
	**DINOv2 ViT-S16**	2.07	0.00	1.37	3.97	3.42	0.00	0.00	7.59	4.11	3.42	9.52	7.53	1.89	2.05
**Mean mAP (%)**	**CellProfiler**	0.78	0.63	0.70	0.99	0.71	1.30	0.80	2.74	2.23	2.02	2.90	2.96	1.91	1.78
	**MAE ViT-B8**	0.67	0.49	0.66	1.05	0.94	1.47	0.65	1.52	1.50	0.99	1.60	1.73	3.11	1.31
	**MAE ViT-B16**	0.74	0.55	0.67	1.00	0.91	1.18	0.75	2.01	2.04	1.16	1.69	1.65	3.02	1.11
	**iTPN HiViT-B8**	0.72	0.46	0.63	1.21	1.08	0.74	0.61	2.23	2.53	1.73	2.19	2.50	2.63	1.67
	**iTPN HiViT-B16**	0.91	0.46	0.63	1.44	1.43	0.79	0.64	2.54	3.04	1.89	2.34	2.82	4.05	2.05
	**DINO ViT-B16**	0.96	0.39	0.74	1.14	1.50	0.55	0.77	4.25	3.84	2.82	2.66	3.85	4.14	3.03
	**DINOv2 ViT-B16**	1.42	0.46	0.68	1.65	1.51	0.78	0.69	3.48	3.10	2.37	2.48	3.85	3.42	2.38
	**DINOv2 ViT-B16 cross-batch (no aug)**	1.67	1.04	1.01	1.57	1.59	2.08	0.71	3.76	3.98	2.28	2.28	3.53	3.64	2.29
	**DINOv2 ViT-B16 cross-batch**	2.10	1.25	1.42	1.83	2.30	2.30	0.98	4.41	4.74	2.69	2.51	4.40	4.53	3.00
	**DINOv2 ViT-B16 one-source**	0.74	0.51	0.69	1.26	0.84	0.70	0.62	2.94	2.93	2.20	2.43	2.80	3.15	2.28
	**DINOv2 ViT-B16 (short sequence)**	1.23	0.44	0.73	1.34	1.51	0.80	0.81	3.95	3.45	2.47	2.47	3.87	3.37	2.45
	**DINOv2 ViT-B8**	1.04	0.41	0.55	1.42	1.31	0.62	0.70	2.46	2.42	1.85	2.30	3.50	2.82	1.63
	**DINOv2 ViT-B8 (short sequence)**	1.51	0.52	0.84	1.50	1.67	1.40	0.89	4.26	3.38	2.74	2.56	4.18	4.29	2.77
	**DINOv2 ViT-S16**	0.97	0.42	0.68	1.32	1.07	1.16	0.65	3.14	2.27	1.94	2.15	2.55	4.16	1.87

Table S5. Evaluation of the Phenotypic Distinctiveness (PNSBW) task on the JUMP-Target2 14 positive controls (poscon_diverse) collected from all sources. PNSBW-based task offers robustness against experimental-batch and well-position effects.

	Without Post-processing		With Post-processing	
**Model**	**Retrieved (%)**	**Mean mAP (%)**	**Retrieved (%)**	**Mean mAP (%)**
**CellProfiler**	85.55	30.10	87.91	74.95
**MAE ViT-B8**	92.86	31.07	92.86	55.25
**MAE ViT-B16**	92.86	32.38	100.00	52.73
**iTPN HiViT-B8**	100.00	35.11	100.00	72.86
**iTPN HiViT-B16**	100.00	39.28	100.00	78.66
**DINO ViT-B16**	85.71	39.27	100.00	98.93
**DINOv2 ViT-B16**	57.14	38.28	100.00	97.20
**DINOv2 ViT-B16 cross-batch (no aug)**	78.57	46.34	100.00	97.76
**DINOv2 ViT-B16 cross-batch**	85.71	62.54	**100.00**	**99.46**
**DINOv2 ViT-B16 one-source**	100.0	41.66	100.00	87.69
**DINOv2 ViT-B16 (short sequence)**	71.43	47.79	100.00	97.36
**DINOv2 ViT-B8**	50.00	33.61	100.00	86.94
**DINOv2 ViT-B8 (short sequence)**	85.71	56.11	100.00	99.05
**DINOv2 ViT-S16**	57.14	37.91	100.00	90.14

Table S6 – Computational Efficiency Metrics Over 400 Epochs for SSL Methods. This table presents the training and evaluation times for various self-supervised learning (SSL) methods, including total training time in GPU hours and inference time for an entire plate.

	Training time (GPU hours)	Inference time
**SSL Method**	**Training Set**	**Plate (384 well - 9 FoV)**
**MAE ViT-B16**	1022.4	1min 33s
**iTPN HiViT-B16**	1382.4	2min 17s
**DINO ViT-B16**	2693.1	1min 36s
**DINOv2 ViT-B16**	1548.0	1min 36s

Table S7. Evaluation of the phenotypic activity task on the EU-OPENSCREEN cpg0036 dataset (2,464 compounds). Performance across models reveals the percentage of compounds identified as phenotypically active, which serves as the input set for the downstream biological relevance (NSCB) analysis reported in Table 4.

		Cell Types (Retrieved (%) | mean mAP(%))				
	**Site**	**FMP**		**IMTM**	**MEDINA**	**USC**
	**Model**	**U2OS**	**HepG2**	**HepG2**	**HepG2**	**HepG2**
**Without Post-processing**	**CellProfiler**	10.82 | 7.23	0.90 | 2.24	0.57 | 0.74	0.81 | 2.33	8.64 | 6.47
	**MAE ViT-B16**	10.62 | 7.28	2.15 | 2.45	3.95 | 3.03	2.15 | 3.07	3.20 | 5.92
	**iTPN HiViT-B16**	23.84 | 14.06	3.12 | 3.10	10.17 | 4.36	2.88 | 4.74	10.79 | 7.09
	**DINO ViT-B16**	23.97 | 14.04	6.20 | 6.27	21.19 | 7.02	15.53 | 6.24	31.10 | 11.37
	**DINOv2 ViT-B16**	24.01 | 14.05	5.96 | 6.07	20.62 | 7.35	13.87 | 6.13	29.08 | 10.45
**With Post-processing**	**CellProfiler**	97.38 | 69.28	93.37 | 55.37	92.04 | 52.65	84.98 | 44.38	95.70 | 47.22
	**MAE ViT-B16**	58.80 | 18.40	10.26 | 6.39	46.35 | 16.46	37.51 | 11.22	74.98 | 21.81
	**iTPN HiViT-B16**	83.21 | 35.66	57.95 | 13.24	78.95 | 37.19	71.29 | 27.05	88.73 | 38.92
	**DINO ViT-B16**	85.48 | 48.82	84.35 | 45.16	91.32 | 52.67	82.24 | 44.73	90.02 | 47.66
	**DINOv2 ViT-B16**	88.73 | 51.77	88.69 | 48.01	94.08 | 57.22	83.82 | 46.94	93.15 | 51.53

Table S8 – Quantitative evaluation of overall MoA clustering performance after batch effect correction on learned representations from different models on the annotated BBBC021 dataset. Performance metrics include Silhouette Scores and K-NN Graph Connectivity scores, with higher values indicating better clustering.

	BBBC021 MoA Clustering	
**Model**	**Silhouette Score ↑ better**	**k-NN Graph Connectivity ↑ better**
**MAE ViT-B16**	0.0992	0.4361
**iTPN HiViT-B16**	0.3367	0.5429
**DINO ViT-B16**	0.3300	0.5518
**DINOv2 ViT-B16**	**0.4187**	**0.5769**

Table S9 – Comparative performance of different methods using a few-shot learning approach with a KNN classifier to predict 12 MoAs from the BBBC021 dataset. The classification performance was assessed using the Not-Same-Compound (NSC) and Not-Same-Compound-Batch (NSCB) evaluation schemes at various K-values (1 to 5).

	**K**									
	**1**		**2**		**3**		**4**		**5**	
	**NSC**	**NSCB**	**NSC**	**NSCB**	**NSC**	**NSCB**	**NSC**	**NSCB**	**NSC**	**NSCB**
**DINO ViT-B16**	0.8641	0.7500	0.8932	0.837	0.9126	0.8804	0.9515	0.9239	0.9709	0.9457
**DINOv2 ViT-B16**	**0.9126**	0.7935	**0.9417**	0.8261	**0.9612**	0.8587	**0.9612**	0.8913	**0.9709**	0.9348
**MAE ViT-B16**	0.6893	0.6413	0.7767	0.6957	0.8252	0.7391	0.8447	0.7391	0.8738	0.7500
**iTPN HiViT-B16**	0.7961	**0.8804**	0.8350	**0.9130**	0.8544	**0.9348**	0.8835	**0.9457**	0.9126	**0.9457**
**CellProfiler [Singh *et al.*[17]]**	0.9000	0.8500	-	-	-	-	-	-	-	-

Table S10 – Evaluation of the Phenotypic Activity (PNSBW) task on RxRx1 Dataset (1,138 genetic perturbations in 51 experimental batches across 4 cell types), comparing performance across different models. Postprocessing consistently yields significant improvements for both the percentage of retrieved classes and mean mAP, which reflects improved batch effect correction and phenotypic discrimination.

		**Cell Types (Retrieved (%) | mean mAP(%))**			
	**Model**	**HepG2**	**HUVEC**	**RPE**	**U2OS**
**Without Post-processing**	***Baseline**	N/A	49.30 | 31.77	77.24 | 53.19	0.00 | 33.65
	**MAE ViT-B16**	0.00 | 24.38	22.76 | 26.12	0.00 | 28.00	0.00 | 34.33
	**iTPN HiViT-B16**	0.44 | 27.08	65.20 | 40.86	37.96 | 39.98	0.00 | 38.05
	**DINO ViT-B16**	4.39 | 29.88	73.20 | 47.48	74.78 | 56.23	0.44 | 38.82
	**DINOv2 ViT-B16**	1.49 | 27.64	61.95 | 37.79	66.96 | 46.20	0.00 | 34.91
**With Post-processing**	***Baseline**	N/A	**93.41** | 59.71	83.04 | 53.54	55.71 | 62.39
	**MAE ViT-B16**	56.77 | 45.80	80.05 | 52.53	70.39 | 55.07	46.40 | 58.37
	**iTPN HiViT-B16**	75.48 | 51.60	88.14 | 63.63	84.27 | 67.06	60.98 | 66.76
	**DINO ViT-B16**	82.60 | 62.94	89.81 | 69.55	90.77 | 76.90	68.63 | 71.09
	**DINOv2 ViT-B16**	**84.71** | **65.63**	92.18 | **73.79**	**92.71** | **80.22**	**72.93** | **74.70**

*Baseline features were extracted using Acapella (PerkinElmer, Waltham MA), a commercial high-content image analysis software that provides hand-engineered morphological features commonly used in classical cell-profiling workflows.

Table S11 –Overall comparative performance of DINO-based models with different backbone architectures, Vision Transformer and SWIN, on the phenotypic distinctiveness (PNSB) task for JUMP Target2-Compounds.

	Without Post-processing		With Post-processing	
**Model**	**Retrieved (%)**	**Mean mAP (%)**	**Retrieved (%)**	**Mean mAP (%)**
**DINO ViT-B16**	19.93	7.34	**100.00**	**42.27**
**DINO SWINv2-B-W16**	18.30	6.43	93.14	28.20

Table S12. Summary statistics of the EU-OPENSCREEN (cpg0036) dataset analyzed in this study, including counts of experimental batches, plates, wells, images, and compounds.

	Site	Cell type	Batches	Plates	Wells	FoVs	Microscopic Images	Compounds
	**FMP**	**U2OS**	**4**	**28**	**9,940**	**9**	**357,840**	**2,464**
		**HepG2**	**4**	**28**	**9,968**	**9**	**358,848**	**2,464**
	**IMTM**	**HepG2**	**7**	**28**	**9,968**	**9**	**358,680**	**2,464**
	**MEDINA**	**HepG2**	**10**	**28**	**9,968**	**9**	**358,848**	**2,464**
	**USC**	**HepG2**	**7**	**28**	**9,968**	**9**	**358,848**	**2,464**
**Total**	**4**	**2**	**32**	**140**	**49,812**	**9**	**1,793,064**	**2,464**
**DMSO**	**-**	**-**	**32**	**112**	**3,206**	**9**	**199,572**	**-**

Table S13. Comparative performance of overall mean average precision (mAP) per MoA for CellProfiler and DINO. Results are shown for MoAs that were significantly retrieved with cross-site agreement (≥3 sites), as shown in Figure 8, using the phenotypic consistency (NSCB) task evaluated independently at each screening site.

Mechanism of Action (MoA)	Number of Compounds	Chemical Diversity	mAP CellProfiler (%)	mAP DINO (%)	Δ(mAP DINO – mAP CellProfiler) (%)
ATPase inhibitor (ATPasei)	8	0.62	14.88	12.86	-2.02
Aurora kinase inhibitor (AURKi)	10	0.90	22.08	12.13	-9.95
FAAH inhibitor(FAAHi)	3	1.00	11.71	24.78	13.07
HSP inhibitor (HSPi)	9	0.78	42.53	58.44	15.91
MEK inhibitor (MEKi)	8	1.00	13.89	17.39	3.50
microtubule inhibitor (MTi)	3	1.00	41.43	44.17	2.74
PARP inhibitor (PARPi)	8	1.00	11.01	10.03	-0.98
rho associated kinase inhibitor (ROCKi)	7	0.71	32.11	36.22	4.11
tubulin polymerization inhibitor (TPi)	6	0.83	23.93	30.24	6.31
dihydrofolate reductase inhibitor (DHFRi)	7	0.85	10.69	11.35	0.66
PI3K inhibitor (PI3Ki)	12	1.00	6.12	6.23	0.11
EGFR inhibitor (EGFRi)	14	0.93	4.90	7.37	2.47
topoisomerase inhibitor (Topoi)	4	1.00	9.37	10.67	1.30
CDK inhibitor (CDKi)	11	1.00	4.92	5.16	0.24
ribonucleotide reductase inhibitor (RNRi)	4	0.71	11.02	15.73	4.71
glucocorticoid receptor agonist (GR agonist)	11	0.48	4.21	10.59	6.38
PDGFR tyrosine kinase receptor inhibitor	12	1.00	2.36	2.54	0.18
bromodomain inhibitor (BRDi)	9	1.00	2.85	7.65	4.80
PLK inhibitor (PLKi)	2	1.00	2.74	32.64	29.90
HMGCR inhibitor (HMGCRi)	6	0.67	18.70	16.40	-2.30
KIT inhibitor (KITi)	11	1.00	3.39	3.08	-0.31
ATM kinase inhibitor (ATMi)	3	1.00	9.13	7.97	-1.16
RAF inhibitor (RAFi)	9	1.00	4.74	6.57	1.83
calpain inhibitor (CALPi)	2	1.00	6.91	17.77	10.86
VEGFR inhibitor (VEGFRi)	17	0.94	2.41	2.21	-0.20
histamine receptor antagonist (H1RA)	12	1.00	2.13	3.18	1.05
tricyclic antidepressant (TCA)	3	1.00	4.18	14.32	10.14

3. Supplementary Figures

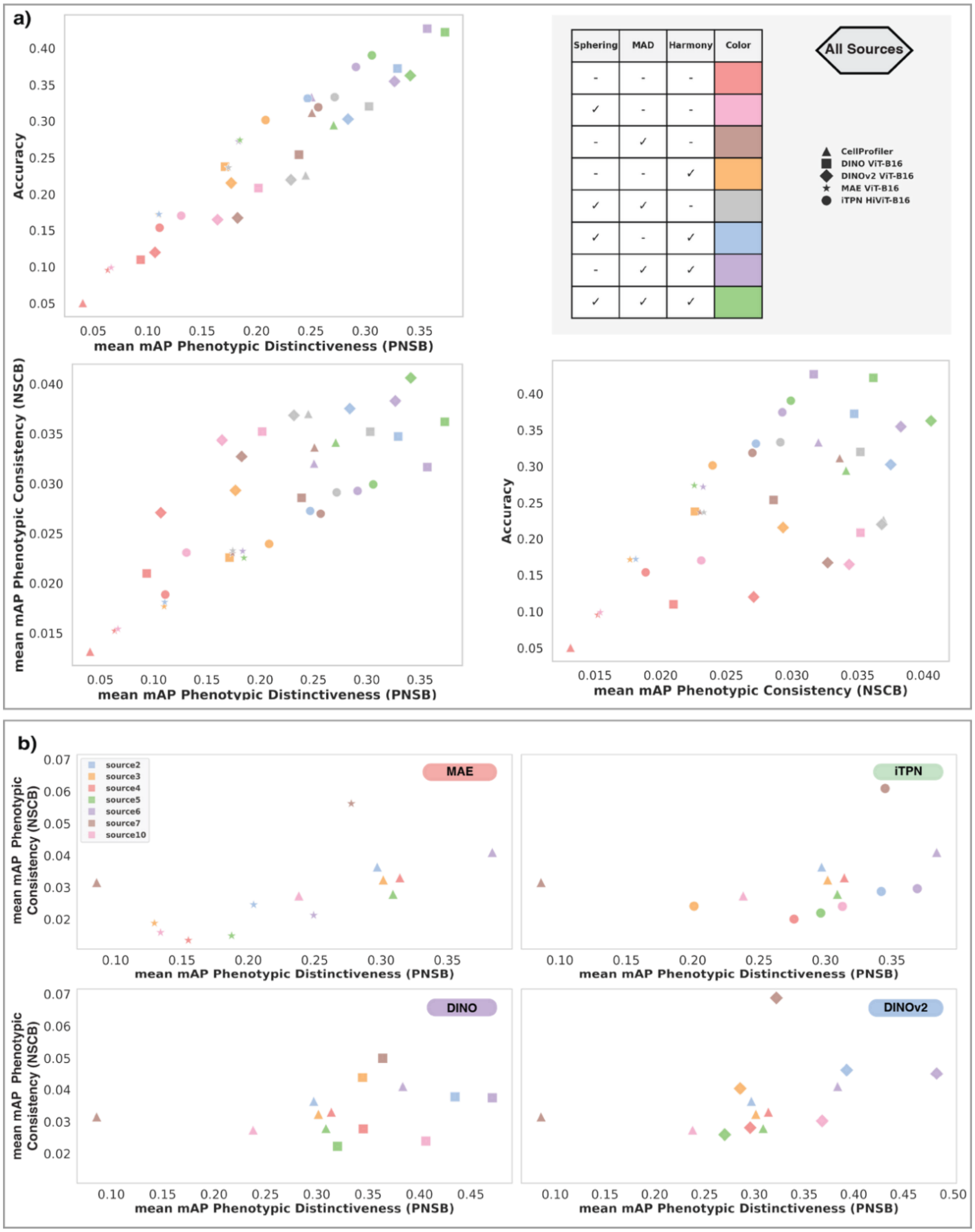

Fig. S1. Postprocessing of the learned representation for batch effect mitigation demonstrated on JUMP-Target2-Compounds: a) Distribution of the overall retrieval performance across different methods and average-aggregated across all sources—evaluating impact of various postprocessing combinations. Performance is assessed on downstream tasks of phenotypic distinctiveness (reproducibility) and phenotypic consistency (biological relevance). b) Performance comparison across all methods for each source individually, using the best postprocessing combination. Results are always compared against the baseline method, CellProfiler.

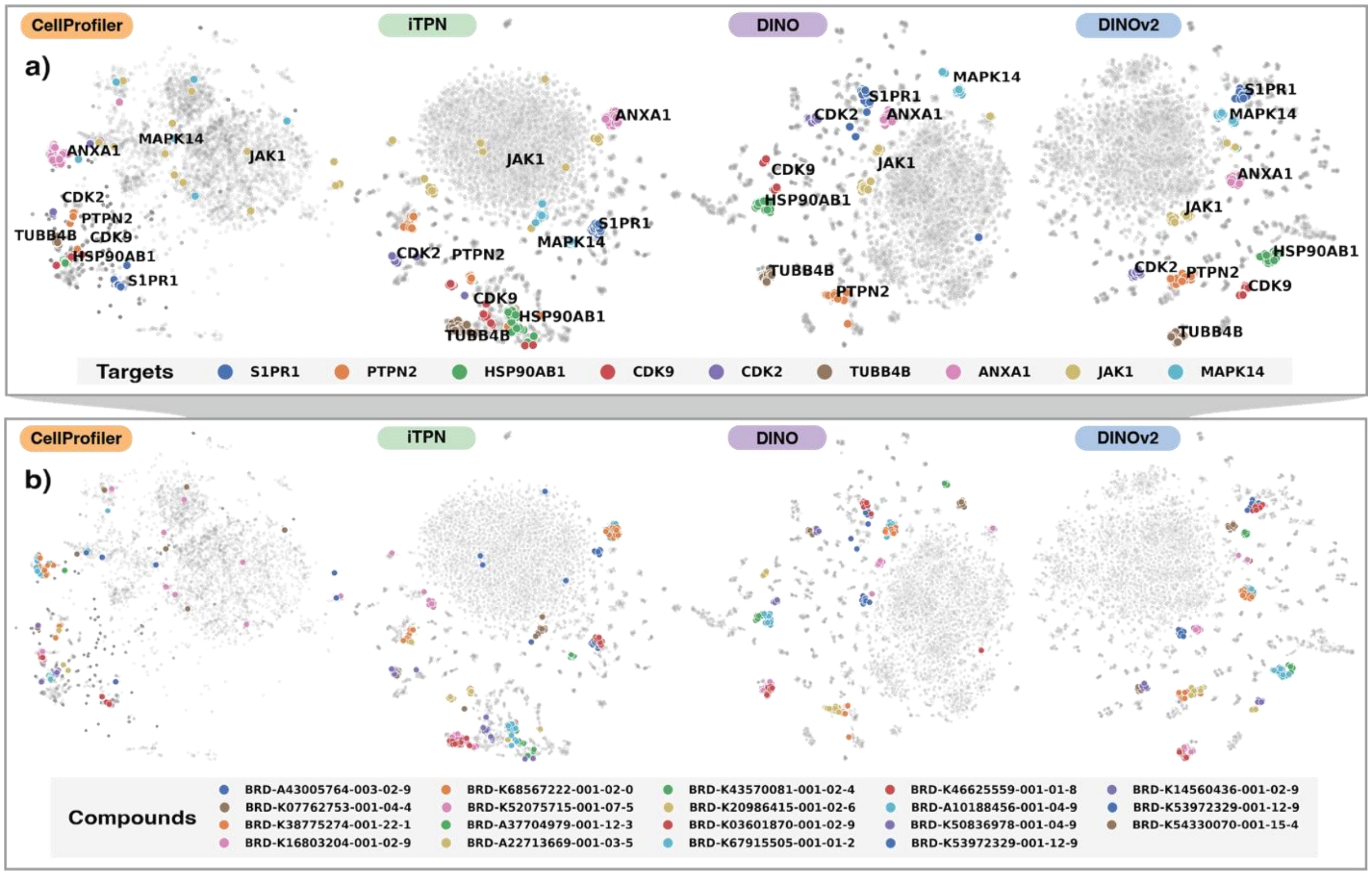

Fig. S2. t-SNE Visualization of postprocessed embeddings from Target2-Compounds (Source6) across different models: a) distribution of targets retrieved based on the phenotypic consistency (NSCB) task (mAP ≥ 0.25), showing a total of 9 unique targets across all methods, with each target represented by two compounds as shown in (b). While 6 of these targets were retrieved by the different methods at the retrieval threshold (mAP ≥ 0.25), DINOv2 demonstrated greater robustness by retrieving three additional targets (MAPK14, JAK1, and CDK9), forming more compact clusters compared to those learned by other methods, specifically CellProfiler.

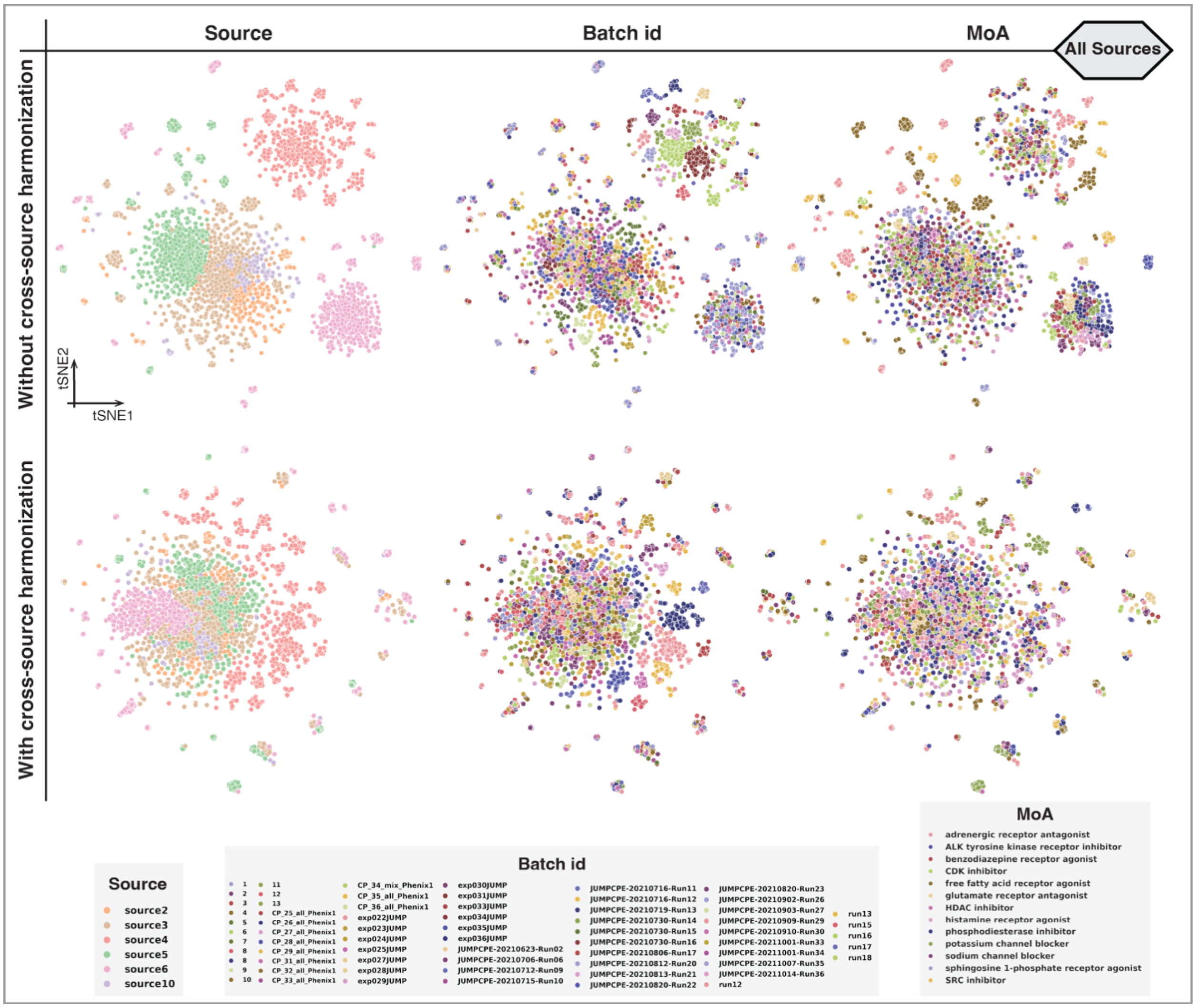

Fig. S3. Impact of batch effects on the alignment of multi-source data (illustrated on MoA subset annotated for JUMP-Target2 compounds): the t-SNE plots illustrate the challenges of batch effects across multi-source data before and after harmonization using the Harmony algorithm. In this multi-source harmonization analysis, the Harmony algorithm used the term "batch" as data generated within the same source to correct for cross-source variations. The t-SNE plots are colored by Source, Experimental Batch, and MoA: "By Source" highlights significant variations between data sources; "By Batch ID" shows distinct clusters indicating strong batch effects; and "By MoA" reveals overlapping clusters, suggesting confounding by cross-source variations.

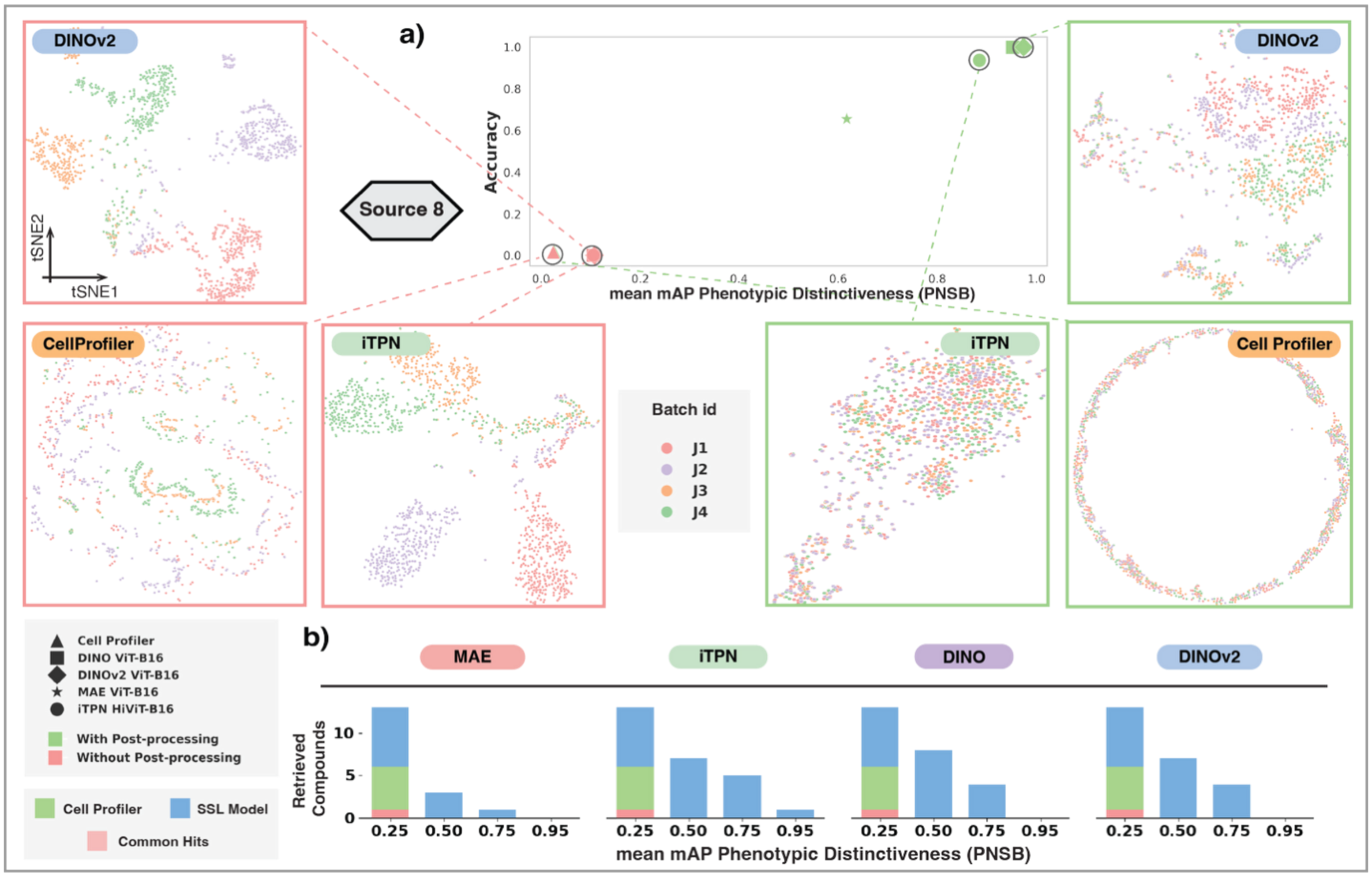

Fig. S4. Comparative performance on Source8 for JUMP-Target2 compounds analysis: (a) Unlike SSL-based models, CellProfiler features do not show structural information (t-SNE maps) and significantly underperform on the phenotypic distinctiveness (PNSB) task (b). This low performance and noisy distribution in the CellProfiler t-SNE plot are mainly due to limitations in the feature engineering pipeline. Despite performing well on other sources, the standard Pycytominer pipeline shows limitations when applied to Source8, requiring further optimization in the feature selection process to filter out noisy distributions.

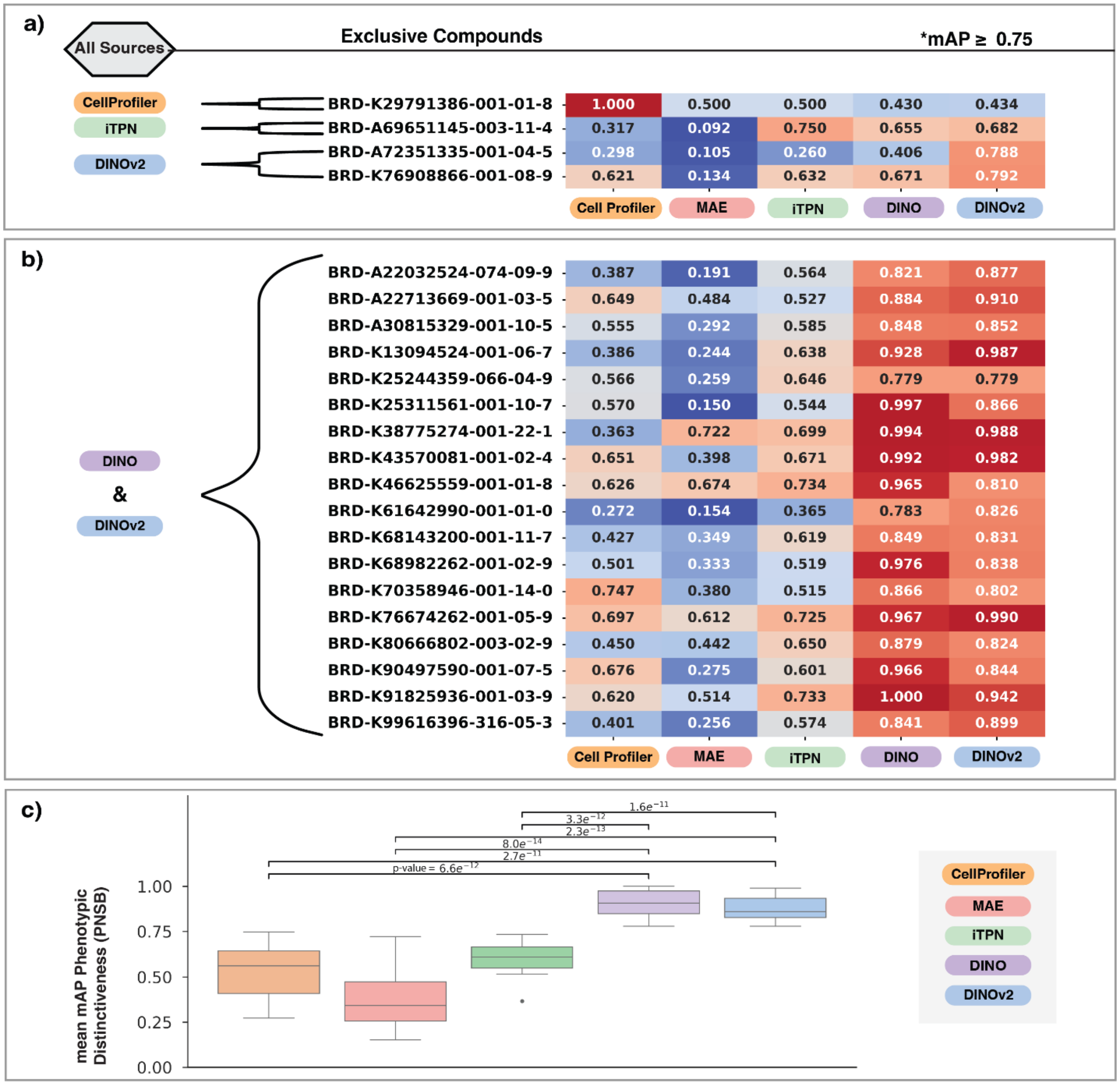

Fig. S5. Analysis of unique and overlapping compounds for reproducibility retrieval task (PNSB) on JUMP-Target2 compounds by all Models (retrieval threshold mAP ≥ 0.75): a) Heatmap of mAP values for compounds uniquely retrieved by each method. b) Heatmap of mAP values for overlapping compounds retrieved by both DINO and DINOv2, which were not well retrieved by other methods (CellProfiler, MAE, and iTPN). c) Box plot comparing mAP values of compounds uniquely retrieved by DINO and DINOv2, shown in (b), against the other models. Box plots display the median (center line), interquartile range (IQR; box bounds, 25th–75th percentiles), and whiskers extending to 1.5× IQR; outliers are shown as individual points. n = 18 compounds per box plot. Exact p-values from two-sided Welch's t-tests are reported above the box plots (c).

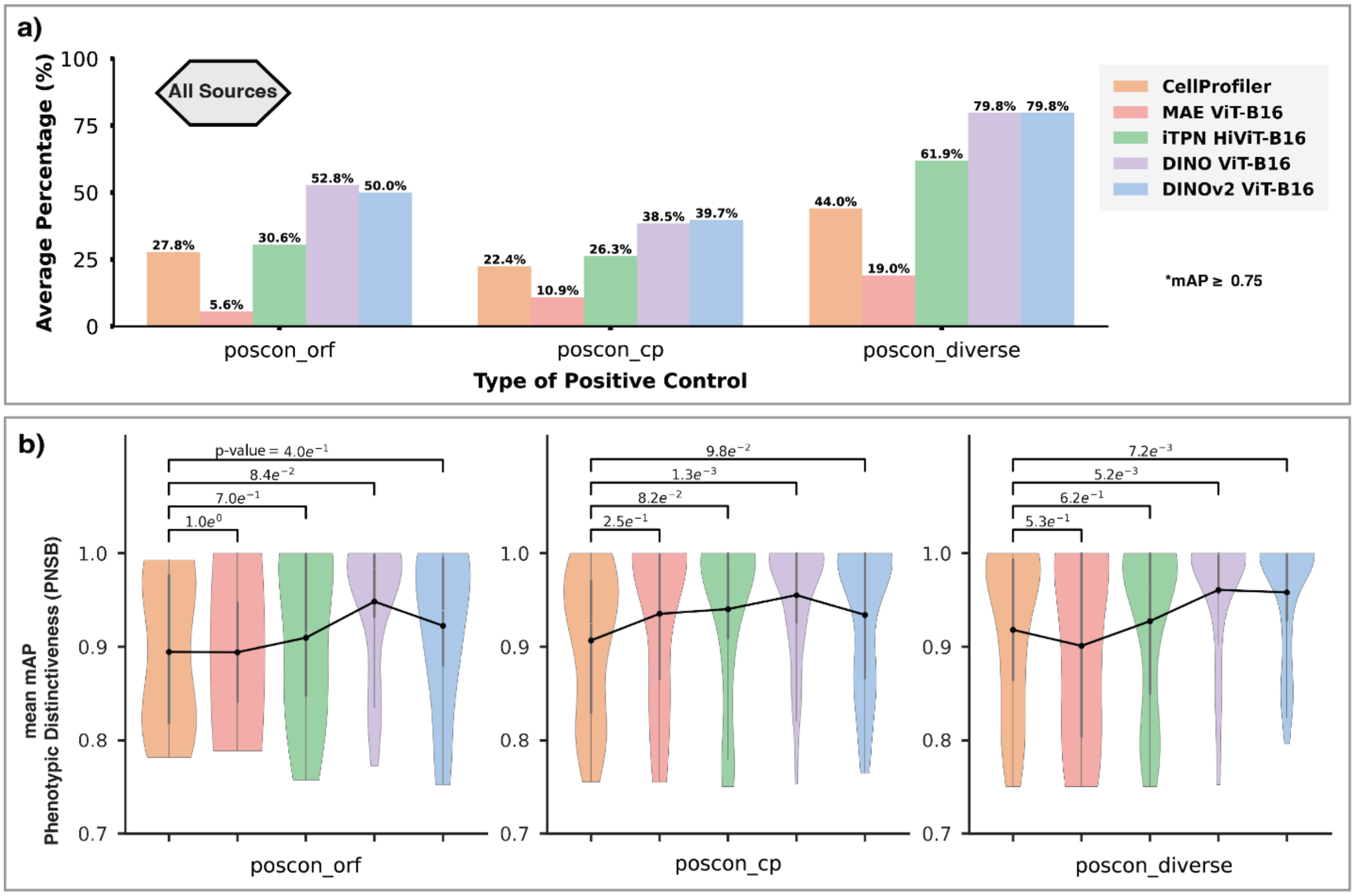

Fig. S6. Comparable performance of different models on the reproducibility retrieval task for JUMP-Target2 positive controls: a) Comparison of various models on replicate retrieval (retrieval threshold mAP ≥ 0.75) for three categories of positive controls: compounds that correlate strongly with overexpression genes (poscon_orf), compounds that strongly correlate with the genes they target (poscon_cp), and pairs of compounds that are strongly correlated with each other but weakly correlated with other poscon_diverse compounds (poscon_diverse). Percentages of significantly retrieved compounds are shown above the bar plots. b) Violin plots showing the distribution of PNSB retrieval performance for each SSL method compared to CellProfiler. The black dot indicates the median, the thick bar represents the interquartile range (IQR; 25th–75th percentiles), and the thin line extends to 1.5× IQR. Sample sizes per positive-control group and method are as follows: poscon_orf: CellProfiler: n = 10 datapoints; MAE ViT-B16: n = 2 datapoints; iTPN HiViT-B16: n = 11 datapoints; DINO ViT-B16: n = 19 datapoints; DINOv2 ViT-B16: n = 18 datapoints | poscon_cp: CellProfiler: n = 35 datapoints; MAE ViT-B16: n = 17 datapoints; iTPN HiViT-B16: 41 datapoints; DINO ViT-B16: n = 60 datapoints; DINOv2 ViT-B16: n = 62 datapoints | poscon_diverse: CellProfiler: n = 37 datapoints; MAE ViT-B16: 16 datapoints; iTPN HiViT-B16: 52 datapoints; DINO ViT-B16: n = 67 datapoints; DINOv2 ViT-B16: n = 67 datapoints. Datapoints represent observations of positive control compounds across JUMP sources and batches. Exact p-values from two-sided Welch's t-tests are reported above the violin plots.

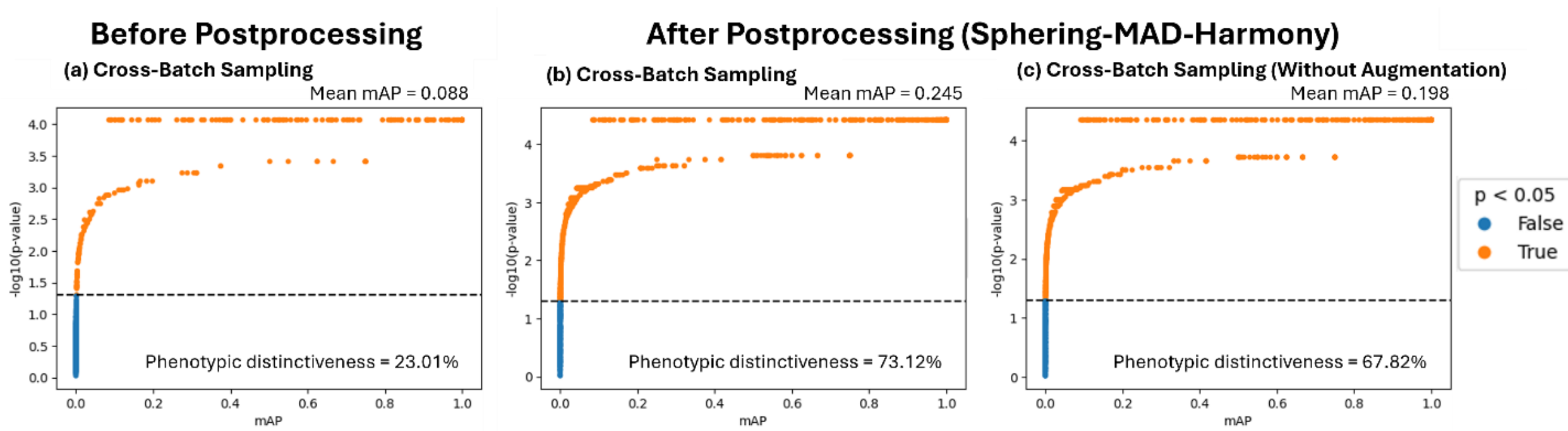

Fig. S7. Phenotypic distinctiveness (PNSB) evaluated on DINOv2 ViT-B16 representations of the entire Source 6 dataset (~59k compounds across 13 experimental batches, excluding DMSO): (a) shows the baseline performance before batch effect correction, while (b-c) demonstrate substantial improvements after correction. Additionally, (b-c) illustrate further performance gains achieved by incorporating data augmentation into the cross-batch sampling experiment (significance is based on corrected p-value).

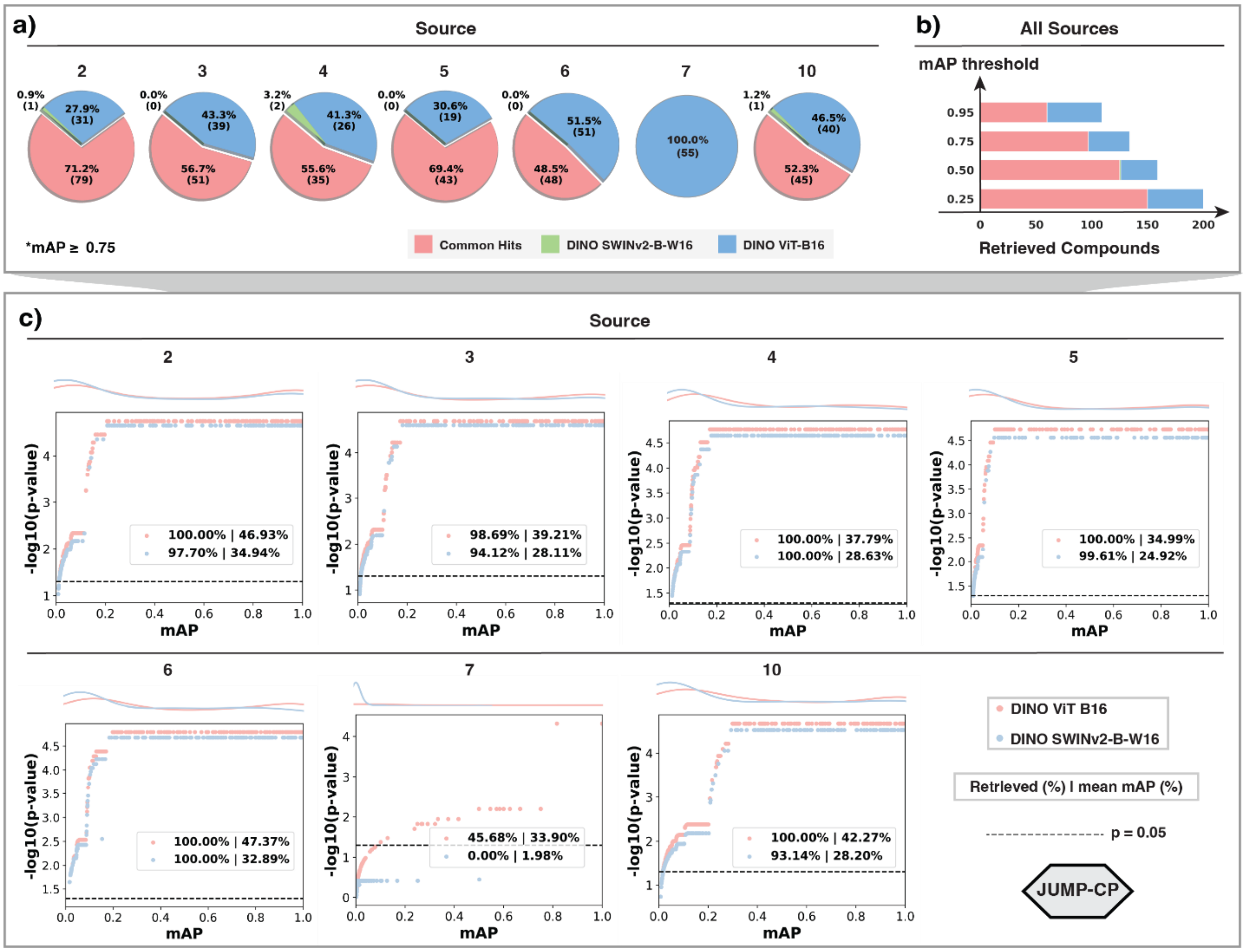

Fig. S8. Source-wise comparison of phenotypic distinctiveness (PNSB) performance between ViT and SWIN architectures. The plots illustrate the overlap and unique retrievals at various retrieval thresholds (a-b), and overall quantitative performance metrics for each method across multiple sources (c).

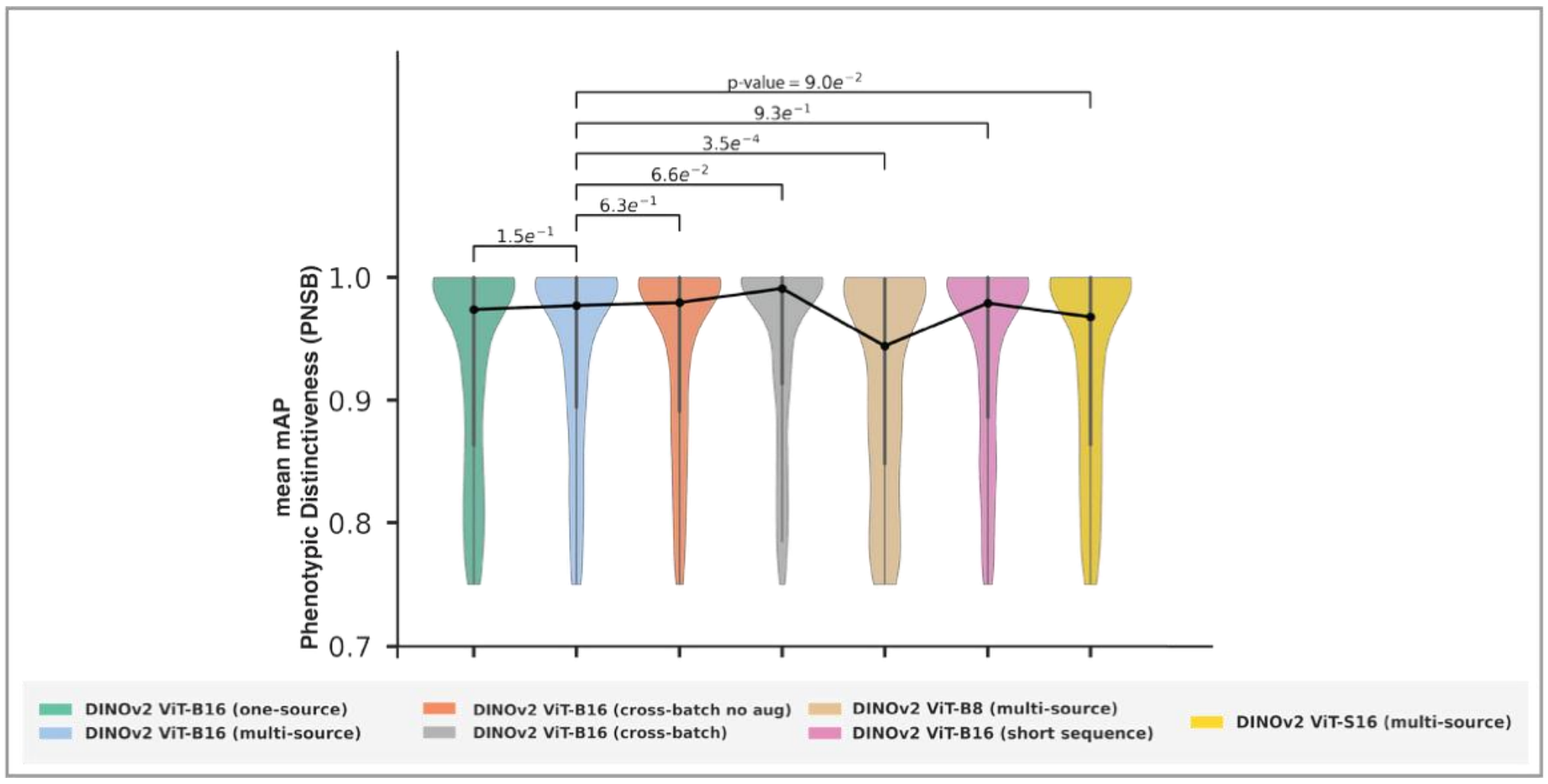

Fig. S9. Statistical evaluation of various training strategies on model performance compared to the baseline model termed "DINOv2 ViT-B16 (multi-source)": distribution of various model performance on the phenotypic distinctiveness (PNSB) task at (mAP ≥ 0.75) from all sources. The analysis reveals statistical significance only observed in the retrieval performance between baseline and the model pretrained with a higher token resolution (DINOv2 ViT-B8). Overall, this comparison underscores the robustness and stability of the training strategy employed in our baseline DINOv2 model. Violin plots showing the distribution of PNSB retrieval performance for each DINOv2 model from the ablation study compared to baseline DINOv2 model. The black dot indicates the median, the thick bar represents the interquartile range (IQR; 25th–75th percentiles), and the thin line extends to 1.5× IQR. Sample sizes per method are as follows: DINOv2 ViT-B16 (one-source): n = 117 compounds (423 datapoints); DINOv2 ViT-B16 (multi-source): n = 125 compounds (448 datapoints); DINOv2 ViT-B16 (cross-batch no aug): n = 127 compounds (472 datapoints); DINOv2 ViT-B16 (cross-batch): n = 139 compounds (569 datapoints); DINOv2 ViT-B8 (multi-source): n = 84 compounds (240 datapoints); DINOv2 ViT-B16 (short sequence): n = 132 compounds (496 datapoints); DINOv2 ViT-S16 (multi-source): n = 110 compounds (392 datapoints). Datapoints represent total observations across JUMP sources and batches. Exact p-values from two-sided Welch's t-tests are reported above the violin plots.

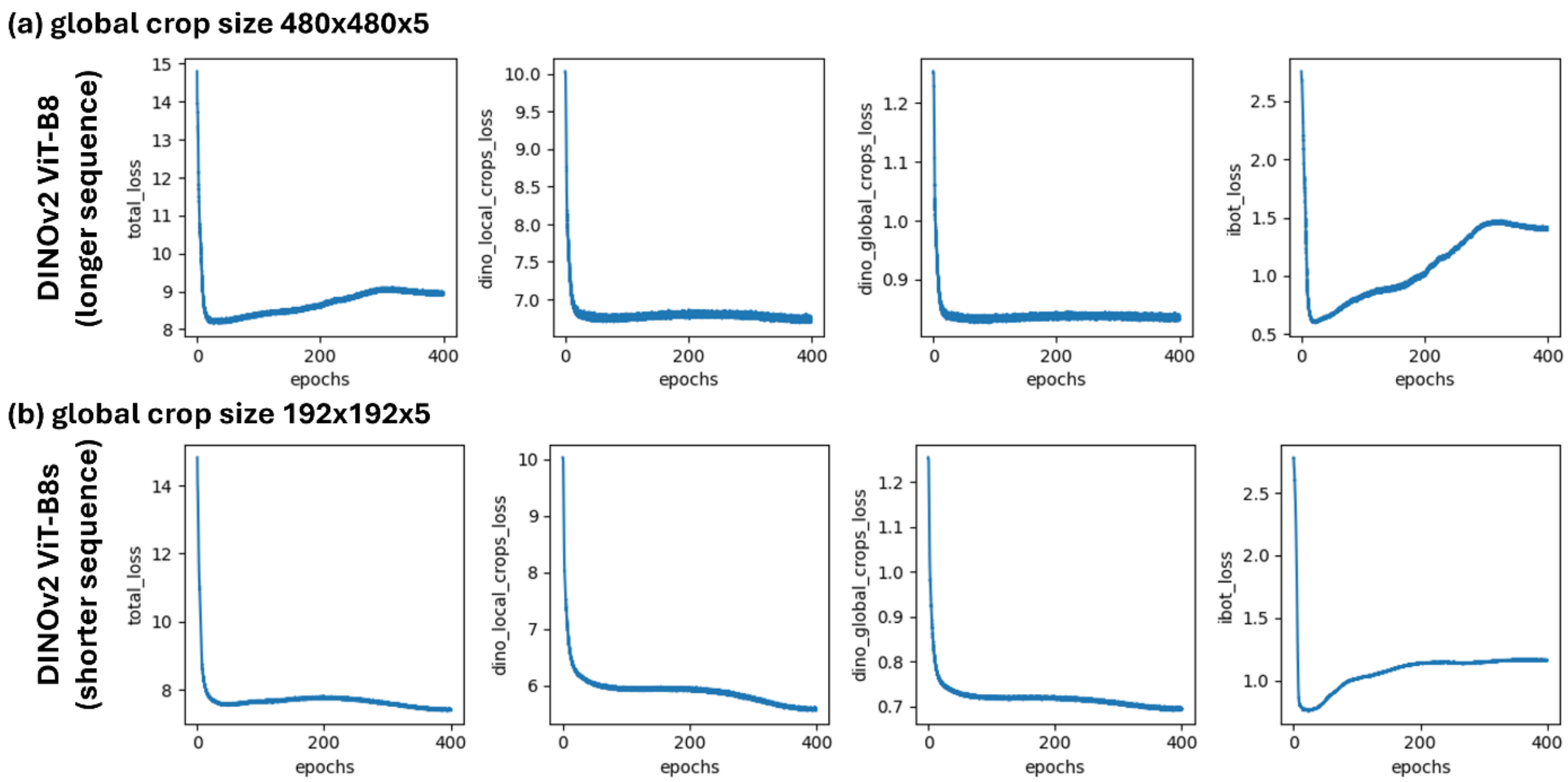

Fig. S10. Loss distribution revealing different dynamics of DINOv2 optimization at higher token resolution with a patch size of 8×8, using a base model architecture with 86 million parameters: (a) ViT-B8 pretrained on a longer token sequence exhibits greater divergence during iBOT loss optimization, indicating underlying challenges in the optimization process; (b) ViT-B8s pretrained on a shorter token sequence demonstrates overall better convergence.

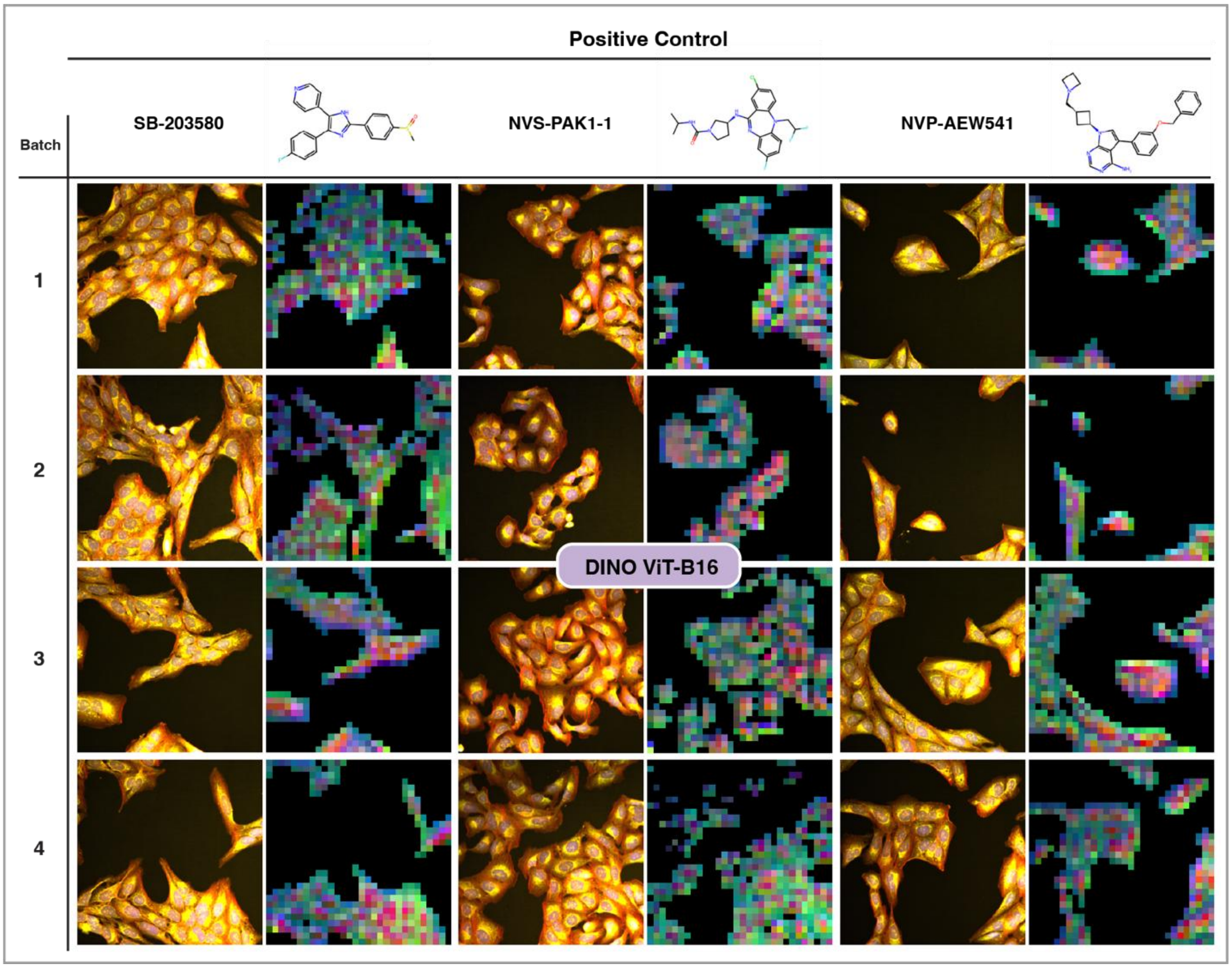

Fig. S11. Analysis of local representations of DINO ViT-B16 cellular semantics across four independent batches for three different compounds.

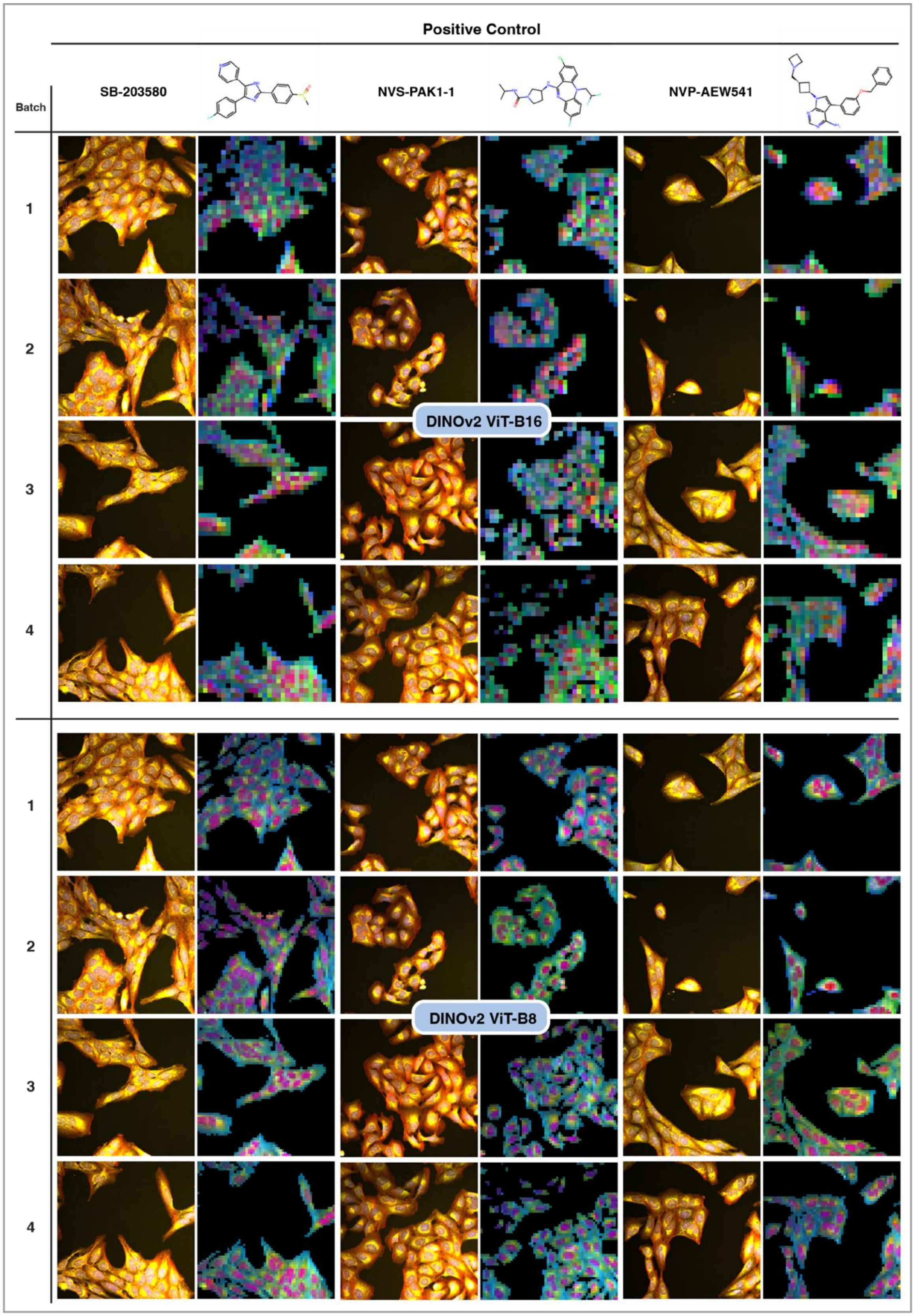

Fig. S12. Analysis of local representations of DINOv2 ViT-B16 and DINOv2 ViT-B8 cellular semantics across four independent batches for three different compounds.

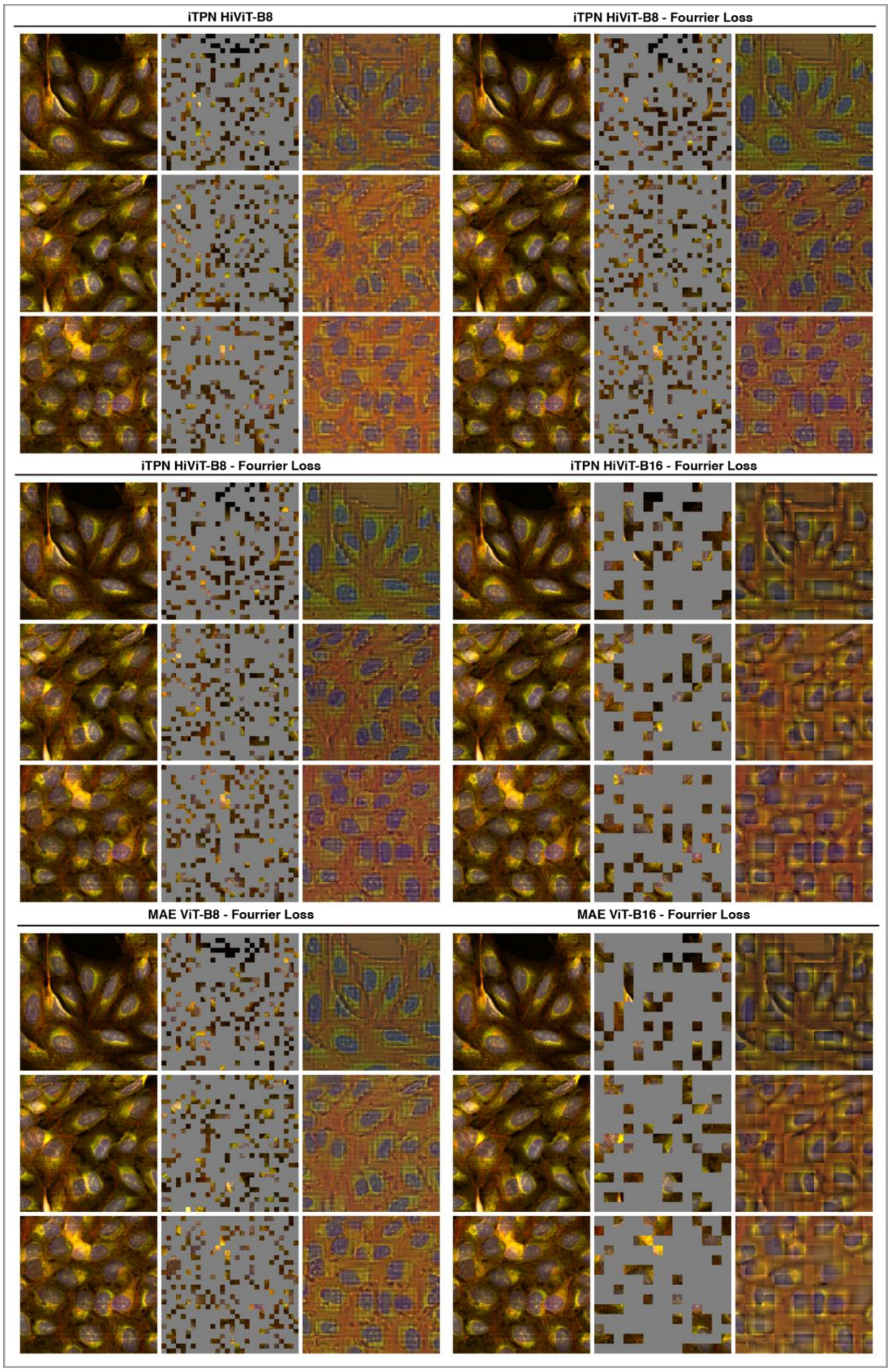

Fig. S13. Visualizing reconstructions on random test images from the JUMP-CP dataset. For each dataset column, we show a triplet consisting of the original image (left), the masked input (middle), and the reconstruction (right). For these models, 75% of the patches constructed from the 224 x 224 random crop of the full image are randomly masked. Reconstruction results are compared for iTPN HiViT-B16, iTPN HiViT-B8, MAE ViT-B16, and MAE ViT-B8. Additionally, the impact of incorporating the Fourier Loss term is evaluated for iTPN HiViT-B8.

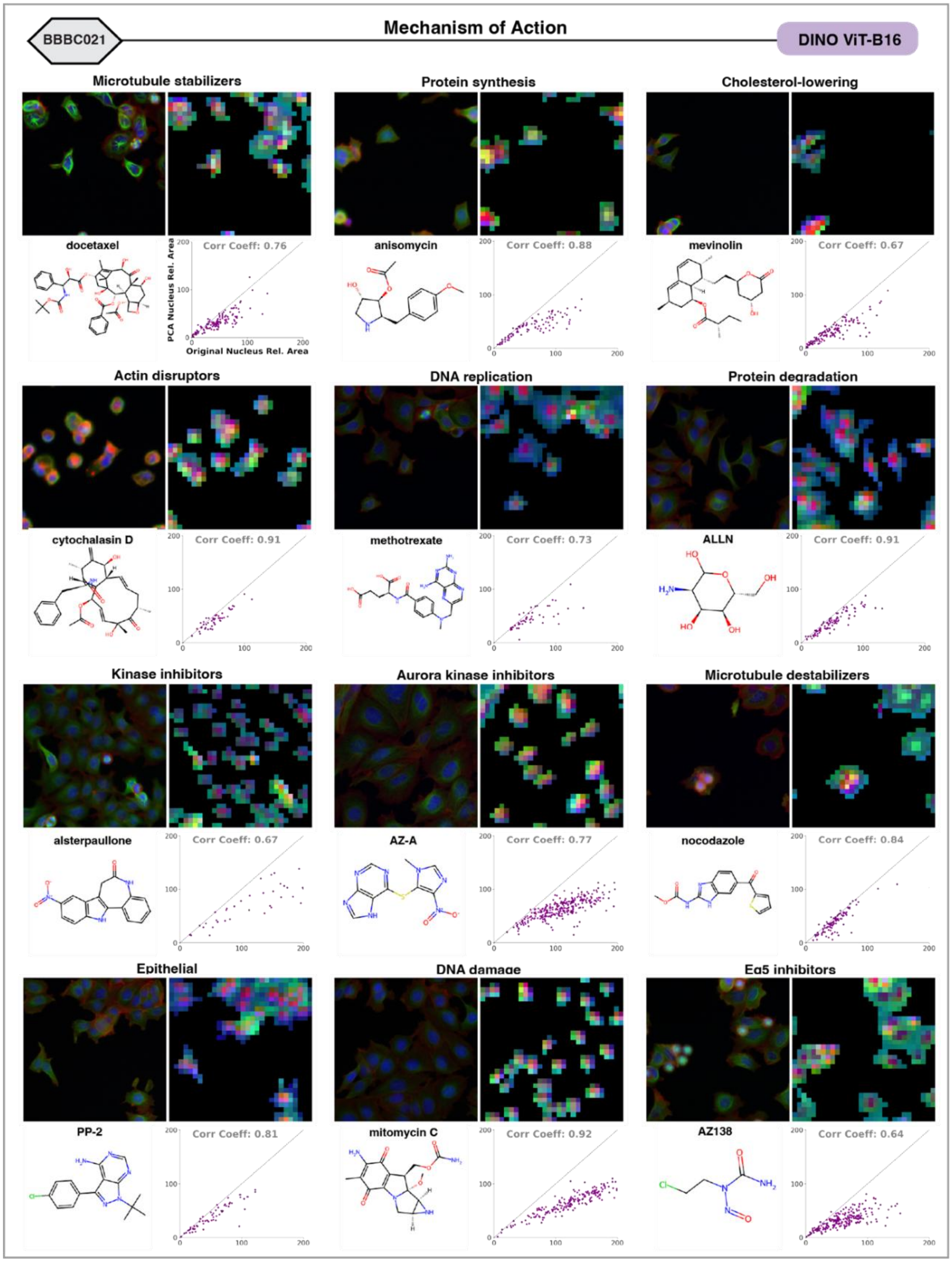

Fig. S14. Generalizability of U2OS-pretrained DINO model on BBBC021 dataset: Analysis of local representations extracted from a novel cell type, MCF-7 breast cancer cells, based on zero-shot learning using the U2OS-pretrained DINO model. PCA-based analysis of local representations reveals cellular semantics across 12 different mechanisms of action (MoAs). Each MoA is represented by an HCI image of a selected compound (left) and its counterpart spatially-mapped PCA image of the local representation (right). The scatter plots reveal strong correlations between the areas of the original nucleus and the SSL-derived nucleus identification.

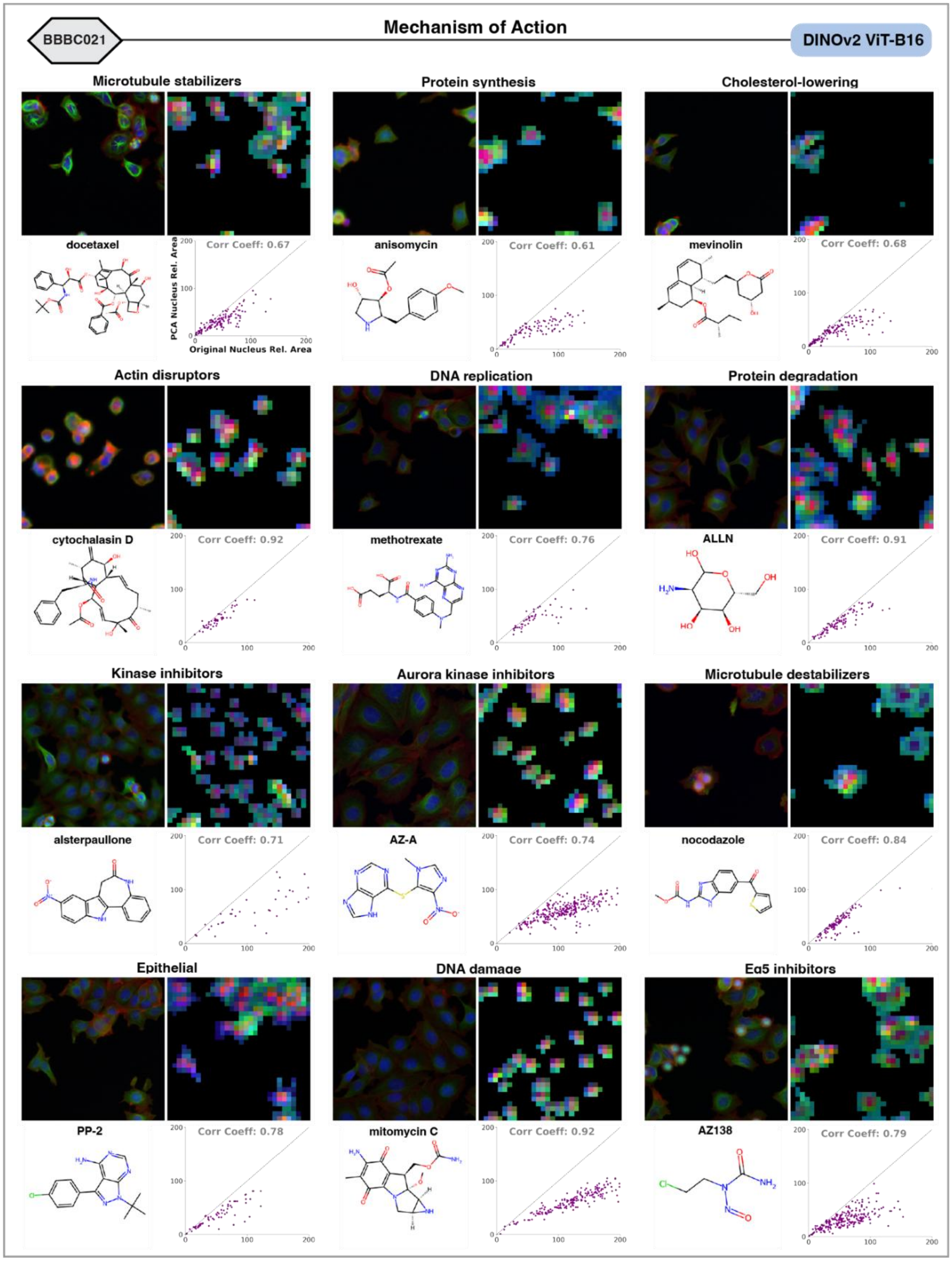

Fig S15. Generalizability of U2OS-pretrained DINOv2 model on BBBC021 dataset: Analysis of local representations extracted from a novel cell type, MCF-7 breast cancer cells, based on zero-shot learning using the U2OS-pretrained DINOv2 model. PCA-based analysis of local representations reveals cellular semantics across 12 different mechanisms of action (MoAs). Each MoA is represented by an HCI image of a selected compound (left) and its counterpart spatially-mapped PCA image of the local representation (right). The scatter plots reveal strong correlations between the areas of the original nucleus and the SSL-derived nucleus identification.

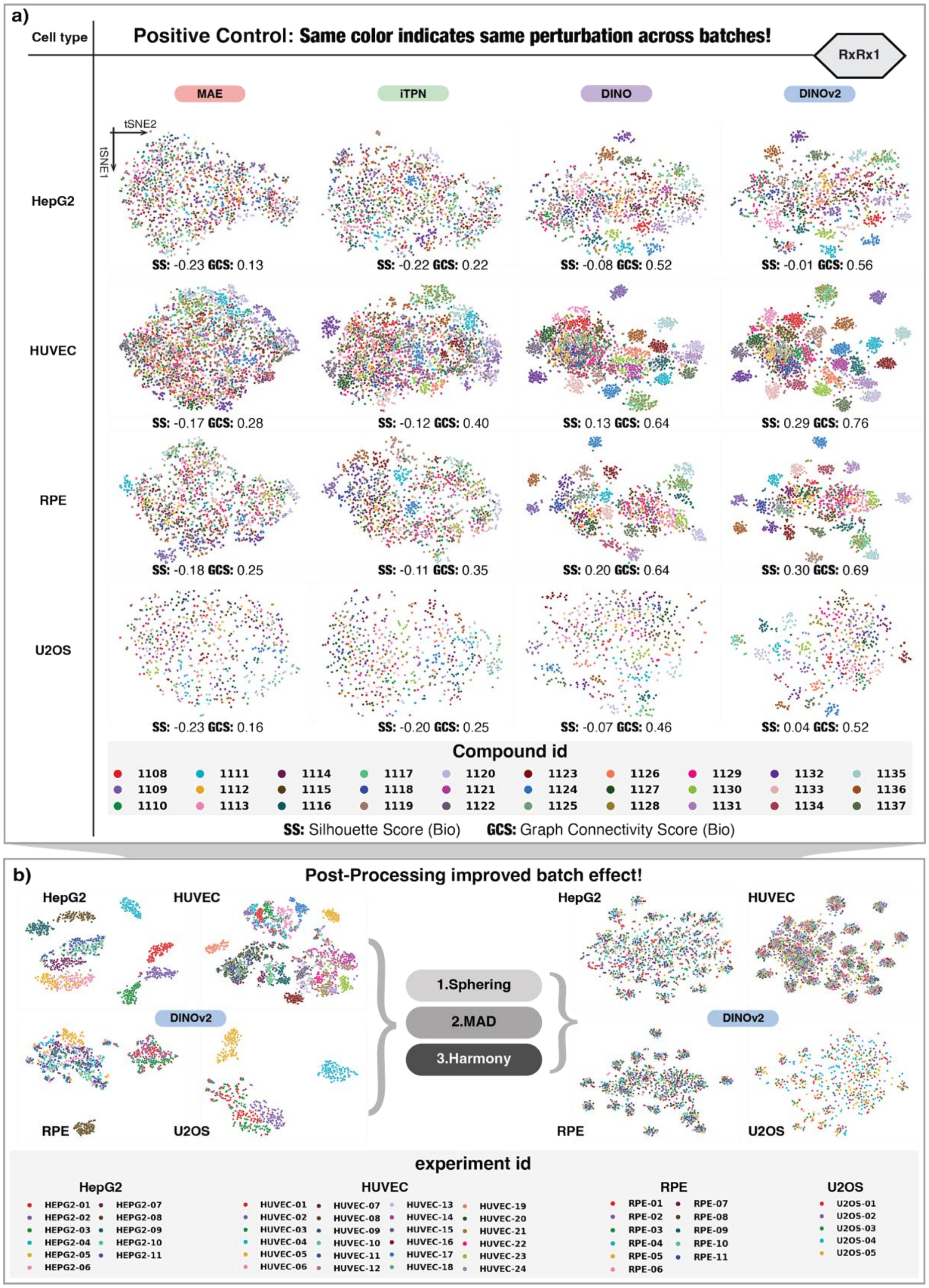

*Fig. S16. t-SNE visualization of zero-shot embeddings from different SSL models, illustrating the clustering quality of 30 positive control compounds across four cell types in the RxRx1 dataset: a) t-SNE maps of well-level embeddings after post-processing, revealing variable clustering performance across different models, with DINO and DINOv2 demonstrating better phenotypic separation compared to the masked-based methods, b) highlights how post-processing mitigates technical variations from 51 experimental batches, as demonstrated by the DINOv2 embeddings (see **Supplementary Figure S15** for details across all SSL models). Quantitative assessment of the clustering in (a) was performed using the Silhouette Score (SS) and Graph Connectivity Score (GCS), where higher scores indicate better capture of relevant phenotypic signals. Overall, these visualizations underscore the generalizability and robustness of DINO and DINOv2 in encoding meaningful biological signals across diverse cell types and experimental batches.*

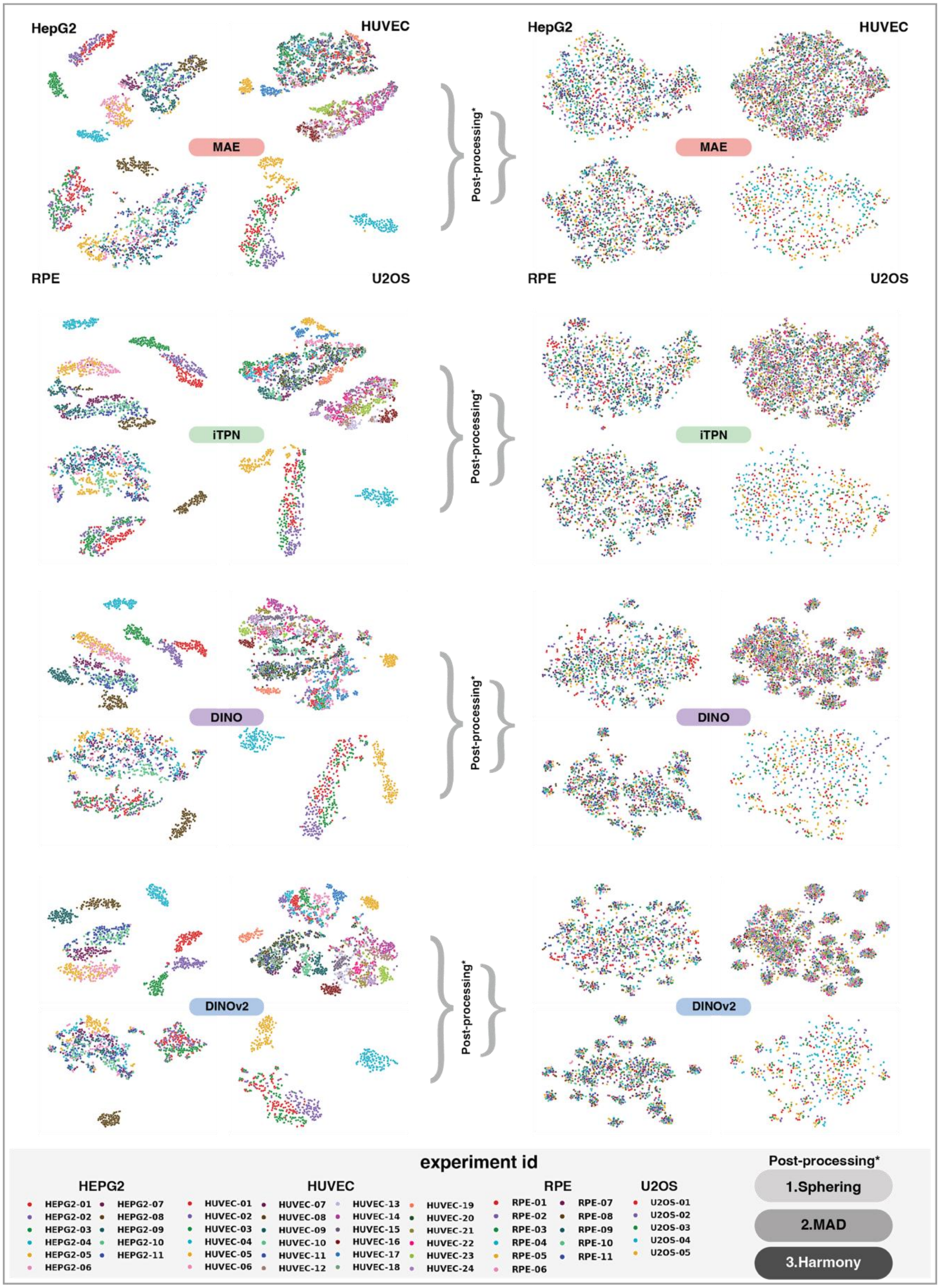

Fig. S17. t-SNE maps of zero-shot embeddings from different SSL models, showing the effect of postprocessing on the clustering quality of 30 positive control compounds across four cell types in the RxRx1 dataset. Each point represents a lower dimensional visualization of a well-level embedding.

References:

1 Way, G. *et al.* Morphology and gene expression profiling provide complementary information for mapping cell state *Cell Systems* **13**, 911-923. e919 (2022).

2 Arevalo, J. *et al.* Evaluating batch correction methods for image-based cell profiling. *Nat Commun* **15**, 6516 (2024). https://doi.org:10.1038/s41467-024-50613-5

3 Ding, C. & Peng, H. Minimum redundancy feature selection from microarray gene expression data. *Journal of bioinformatics and computational biology* **3**, 185-205 (2005).

4 Cox, M. J. *et al.* Tales of 1,008 small molecules: phenomic profiling through live-cell imaging in a panel of reporter cell lines. *Scientific reports* **10**, 13262 (2020).

5 Zhai, X., Kolesnikov, A., Houlsby, N. & Beyer, L. in *Proceedings of the IEEE/CVF conference on computer vision and pattern recognition.* 12104-12113.

6 Wang, F. *et al.* Scaling Laws in Patchification: An Image Is Worth 50,176 Tokens And More. *arXiv preprint arXiv:2502.03738* (2025).

7 Liu, Z. *et al.* in *Proceedings of the IEEE/CVF international conference on computer vision.* 10012-10022.

8 Liu, Z. *et al.* in *Proceedings of the IEEE/CVF conference on computer vision and pattern recognition.* 12009-12019.

9 Dosovitskiy, A. An image is worth 16x16 words: Transformers for image recognition at scale. *arXiv preprint arXiv:2010.11929* (2020).

10 Vaswani, A. *et al.* Attention is all you need. *Advances in neural information processing systems* **30** (2017).

11 Dee, W., Sequeira, I., Lobley, A. & Slabaugh, G. Cell-vision fusion: A Swin transformer-based approach for predicting kinase inhibitor mechanism of action from Cell Painting data. *iScience* **27**, 110511 (2024). https://doi.org:10.1016/j.isci.2024.110511

12 Kraus, O. *et al.* in *Proceedings of the IEEE/CVF Conference on Computer Vision and Pattern Recognition.* 11757-11768.

13 Seal, S. *et al.* From pixels to phenotypes: Integrating image-based profiling with cell health data as BioMorph features improves interpretability. *Molecular Biology of the Cell* **35**, mr2 (2024).

14 Alizadehsani, R. *et al.* Explainable artificial intelligence for drug discovery and development-a comprehensive survey. *IEEE Access* (2024).

15 Gopalakrishnan, V., Ma, J. & Xie, Z. in *Proceedings of the IEEE/CVF Conference on Computer Vision and Pattern Recognition.* 6988-6996.

16 van Dijk, R., Arevalo, J., Babadi, M., Carpenter, A. E. & Singh, S. Capturing cell heterogeneity in representations of cell populations for image-based profiling using contrastive learning. *PLOS Computational Biology* **20**, e1012547 (2024).

17 Singh, S., Bray, M. A., Jones, T. & Carpenter, A. Pipeline for illumination correction of images for high-throughput microscopy. *Journal of microscopy* **256**, 231-236 (2014).
