# Supplementary material for: Advancing morphological profiling with self-supervised learning through comparative insights into representation learning of cellular perturbations
Source: Nat Methods. 2026 Oct 29;23(11):2181–94. doi: 10.1038/s41592-026-03270-z (PMC13736651; doi:10.1038/s41592-026-03270-z)
Supplement: Supplementary file 2 [file 41592_2026_3270_MOESM2_ESM.pdf]

nature portfolio

Corresponding author(s): Walid M. Abdelmoula and Rui Liao

Last updated by author(s): Jul 7, 2026

Reporting Summary

Nature Portfolio wishes to improve the reproducibility of the work that we publish. This form provides structure for consistency and transparency in reporting. For further information on Nature Portfolio policies, see our Editorial Policies and the Editorial Policy Checklist.

Statistics

For all statistical analyses, confirm that the following items are present in the figure legend, table legend, main text, or Methods section.

n/a	Confirmed	
☐	☒	The exact sample size (*n*) for each experimental group/condition, given as a discrete number and unit of measurement
☐	☒	A statement on whether measurements were taken from distinct samples or whether the same sample was measured repeatedly
☐	☒	The statistical test(s) used AND whether they are one- or two-sided<br>*Only common tests should be described solely by name; describe more complex techniques in the Methods section.*
☒	☐	A description of all covariates tested
☒	☐	A description of any assumptions or corrections, such as tests of normality and adjustment for multiple comparisons
☐	☒	A full description of the statistical parameters including central tendency (e.g. means) or other basic estimates (e.g. regression coefficient) AND variation (e.g. standard deviation) or associated estimates of uncertainty (e.g. confidence intervals)
☐	☒	For null hypothesis testing, the test statistic (e.g. *F*, *t*, *r*) with confidence intervals, effect sizes, degrees of freedom and *P* value noted<br>*Give P values as exact values whenever suitable.*
☒	☐	For Bayesian analysis, information on the choice of priors and Markov chain Monte Carlo settings
☒	☐	For hierarchical and complex designs, identification of the appropriate level for tests and full reporting of outcomes
☒	☐	Estimates of effect sizes (e.g. Cohen's *d*, Pearson's *r*), indicating how they were calculated

Our web collection on statistics for biologists contains articles on many of the points above.

Software and code

Policy information about availability of computer code

Data collection	The public JUMP-CP dataset (cpg0016) from Chandrasekaran et al. [BioRxiv, 2023] was downloaded using AWS CLI instructions provided in the Cell Painting Gallery on the Registry of Open Data on AWS [https://registry.opendata.aws/cellpainting-gallery/]. Detailed documentation is also available at [https://broadinstitute.github.io/cellpainting-gallery].<br>The download of the public BBBC021 dataset [Ljosa et al., Nature Methods, 2012; and Caie et al. Molecular Cancer Therapeutics, 2010] was facilitated by a freely available Python package, which can be found at [https://github.com/giacomodeodato/pybbbc].<br>The public dataset of RxRx1 is provided by [Sypetkowski et al. CVPR 2023], and it can be downloaded at https://www.rxrx.ai/rxrx1<br>The EU-OPENSCREEN Bioactives dataset (cpg0036) is available by [Wolff et al. iScience 2025] and can be downloaded from the Cell Painting Gallery (accession cpg0036-EU-OS-bioactives).
Data analysis	Our data analysis were performed using python-based scripts (Python 3.8.8), mainly based on these libraries: numpy (1.24.3), pandas (1.4.2), scikit-learn (1.0.2), scikit-image (0.18.1), pillow (8.2.0), scipy (1.10.1), pycytominer (1.0.1), tifffile (2021.4.8), matplotlib (3.7.5), seaborn (0.13.2), notebook (6.3.0). Moreover, our transformer-based foundation models were implemented based on public deep learning compute libraries, including CUDA 11.7, xformers (0.0.18), TorchVision (0.15.0), and PyTorch (2.0.0). The version of each library is provided in the parenthesis.

For manuscripts utilizing custom algorithms or software that are central to the research but not yet described in published literature, software must be made available to editors and reviewers. We strongly encourage code deposition in a community repository (e.g. GitHub). See the Nature Portfolio guidelines for submitting code & software for further information.

Data

Policy information about availability of data

All manuscripts must include a data availability statement. This statement should provide the following information, where applicable:

- Accession codes, unique identifiers, or web links for publicly available datasets
- A description of any restrictions on data availability
- For clinical datasets or third party data, please ensure that the statement adheres to our policy

All the datasets used in this work are publicly available:
- The principal JUMP-CP dataset of (cpg0016) [Chandrasekaran et al. BioRxiv, 2023] is available from the Cell Painting Gallery on the Registry of Open Data on AWS (https://registry.opendata.aws/cellpainting-gallery/). These data were downloaded to our company servers and accessed based on the available updates as of February 2023.
- The BBBC021 dataset is available from the Broad Bioimage Benchmark Collection (https://bbbc.broadinstitute.org/BBBC021).
- The RxRx1 dataset is available from Recursion (https://www.rxrx.ai/rxrx1)
- The EU-OPENSCREEN Bioactives dataset (cpg0036) is available by [Wolff et al. iScience 2025] and can be downloaded from the Cell Painting Gallery (accession cpg0036-EU-OS-bioactives).

Research involving human participants, their data, or biological material

Policy information about studies with human participants or human data. See also policy information about sex, gender (identity/presentation), and sexual orientation and race, ethnicity and racism.

Reporting on sex and gender	n/a
Reporting on race, ethnicity, or other socially relevant groupings	n/a
Population characteristics	n/a
Recruitment	n/a
Ethics oversight	n/a

Note that full information on the approval of the study protocol must also be provided in the manuscript.

Field-specific reporting

Please select the one below that is the best fit for your research. If you are not sure, read the appropriate sections before making your selection.

☒ Life sciences ☐ Behavioural & social sciences ☐ Ecological, evolutionary & environmental sciences

For a reference copy of the document with all sections, see nature.com/documents/nr-reporting-summary-flat.pdf

Life sciences study design

All studies must disclose on these points even when the disclosure is negative.

Sample size	All foundation models were pretrained using all JUMP unannotated perturbations (119,876 compounds totaling ~1.77 million microscopic images) sampled from all available sources, except for source6, which was held out entirely for evaluation purposes. Model evaluation was performed on the held-out test set (JUMP-Target2-Compounds), which includes a set of annotated perturbations (302 compounds annotated to 160 gene targets) acquired across all sources and experimental batches (8 sources and 98 batches).<br>Three additional independent datasets were used to evaluate model generalizability: BBBC021 (103 treatments; 38 compounds at 1-7 concentrations each), RxRx1 (1,138 treatments), EU-OPENSCREEN Bioactives (2,464 compounds)
Data exclusions	At the time of data access, JUMP imaging data were not available for two sources: source1 and source9. Therefore, we excluded these sources, as our foundation models operate on imaging data.
Replication	All experiments are computational and deterministic. Pretrained models, datasets, embeddings, and evaluation code are publicly released, enabling reproduction of all reported results. We evaluated robustness on two downstream tasks for JUMP-Target2-Compounds: reproducibility and biological relevance. The reproducibility task assesses the representation's ability to retrieve replicates of the same compound from different experimental batches. The biological relevance task evaluates the representation's ability to retrieve compounds targeting the same gene (not-same-compound-and-batch). These analyses were performed on data from 8 JUMP sources. We extended these evaluations to three additional independent datasets: BBBC021, RxRx1, and EU-OPENSCREEN Bioactives.
Randomization	For data randomization and related acquisition information (sample location in plates, compounds distribution, positive control, replicates, experimental batches ... etc.) we refer to the original publications of JUMP-CP [Chandrasekaran et al. BioRxiv, 2023], BBBC021 [Caie et al. Mol Cancer Ther, 2010], RxRx1 is provided by [Sypetkowski et al. CVPR ,2023], EU-OPENSCREEN Bioactives [Wolff et al. iScience, 2025]

Blinding	Blinding was not applicable because all analyses were performed using automated computational pipelines and predefined evaluation metrics, without subjective human assessment of outcomes

Reporting for specific materials, systems and methods

We require information from authors about some types of materials, experimental systems and methods used in many studies. Here, indicate whether each material, system or method listed is relevant to your study. If you are not sure if a list item applies to your research, read the appropriate section before selecting a response.

Materials & experimental systems

n/a	Involved in the study
☒	☐ Antibodies
☒	☐ Eukaryotic cell lines
☒	☐ Palaeontology and archaeology
☒	☐ Animals and other organisms
☒	☐ Clinical data
☒	☐ Dual use research of concern
☒	☐ Plants

Methods

n/a	Involved in the study
☒	☐ ChIP-seq
☒	☐ Flow cytometry
☒	☐ MRI-based neuroimaging

Plants

Seed stocks	n/a
Novel plant genotypes	n/a
Authentication	n/a
